# Mid-Gestational Gene Expression Profile in Placenta and Link to Pregnancy Complications

**DOI:** 10.1371/journal.pone.0049248

**Published:** 2012-11-07

**Authors:** Liis Uusküla, Jaana Männik, Kristiina Rull, Ave Minajeva, Sulev Kõks, Pille Vaas, Pille Teesalu, Jüri Reimand, Maris Laan

**Affiliations:** 1 Human Molecular Genetics Group, Institute of Molecular and Cell Biology, University of Tartu, Tartu, Estonia; 2 Department of Biochemistry, Cellular and Molecular Biology, University of Tennessee, Knoxville, Tennessee, United States of America; 3 Department of Obstetrics and Gynecology, Tartu University Hospital, Tartu, Estonia; 4 Department of Pathology, Tartu University Hospital, Tartu, Estonia; 5 Department of Physiology, Center of Translational Medicine, University of Tartu, Tartu, Estonia; 6 Donnelly Centre, University of Toronto, Toronto, Ontario, Canada; Otto-von-Guericke University Magdeburg, Germany

## Abstract

Despite the importance of placenta in mediating rapid physiological changes in pregnancy, data on temporal dynamics of placental gene expression are limited. We completed the first transcriptome profiling of human placental gene expression dynamics (GeneChips, Affymetrix®; ∼47,000 transcripts) from early to mid-gestation (*n* = 10; gestational weeks 5–18) and report 154 genes with significant transcriptional changes (ANOVA, FDR *P*<0.1). TaqMan RT-qPCR analysis (*n* = 43; gestational weeks 5–41) confirmed a significant (ANOVA and t-test, FDR *P*<0.05) mid-gestational peak of placental gene expression for *BMP5*, *CCNG2*, *CDH11*, *FST*, *GATM*, *GPR183*, *ITGBL1*, *PLAGL1*, *SLC16A10* and *STC1*, followed by sharp decrease in mRNA levels at term (t-test, FDR *P*<0.05). We hypothesized that normal course of late pregnancy may be affected when genes characteristic to mid-gestation placenta remain highly expressed until term, and analyzed their expression in term placentas from normal and complicated pregnancies [preeclampsia (PE), *n* = 12; gestational diabetes mellitus (GDM), *n* = 12; small- and large-for-gestational-age newborns (SGA, LGA), *n* = 12+12]. *STC1* (stanniocalcin 1) exhibited increased mRNA levels in all studied complications, with the most significant effect in PE- and SGA-groups (t-test, FDR *P*<0.05). In post-partum maternal plasma, the highest STC1 hormone levels (ELISA, *n* = 129) were found in women who had developed PE and delivered a SGA newborn (median 731 *vs* 418 pg/ml in controls; ANCOVA, *P* = 0.00048). Significantly higher expression (t-test, FDR *P*<0.05) of *CCNG2* and *LYPD6* accompanied with enhanced immunostaining of the protein was detected in placental sections of PE and GDM cases (*n* = 15). Our study demonstrates the importance of temporal dynamics of placental transcriptional regulation across three trimesters of gestation. Interestingly, many genes with high expression in mid-gestation placenta have also been implicated in adult complex disease, promoting the discussion on the role of placenta in developmental programming. The discovery of elevated maternal plasma STC1 in pregnancy complications warrants further investigations of its potential as a biomarker.

## Introduction

Placenta is a highly specialized temporary organ responsible for the normal progression of pregnancy in mammals. Defects in implantation, placental development and maturation lead to complications in pregnancy and newborns [1]. Aberrant placental expression of apoptosis and inflammation-related genes in early pregnancy was found in recurrent miscarriage [Kristiina Rull, unpublished data] and hydatidiform mole samples [2]. Altered placental transcription of metabolic regulatory genes has been associated to affected fetal growth and maternal pregnancy complications such as preeclampsia (PE) and gestational diabetes mellitus (GDM). Differential expression of some genes is common to several pregnancy complications, serving as biomarkers for malfunctioning placenta (e.g. *FLT1,* Fms-related tyrosine kinase 1; *LEP,* Leptin; *PIGF,* Placental-derived growth factor; *CRH,* Corticotropin-releasing hormone*; ENG,* Endoglin) [3]–[8].

Despite the great importance of placenta in mediating the rapid physiological changes in pregnancy, data on the temporal dynamics of human placental gene expression are limited. Large-scale differences in DNA methylation levels between first, second and third trimesters support the gestational-age dependent function of placental genes [9]. So far, only three published studies focus on gene expression in term placenta compared to either early [10] or mid-gestation pregnancy [11], or both [12]. All studies agree that global gene expression undergoes a profound transformation at the end of pregnancy, affecting up to 25% of placental transcriptome [10] and involving coordinated up- or down-regulation of functionally linked loci to achieve rapid changes in placental function [11]. This appears to support the preparation of the maternal organism for parturition and the fetal organism for postnatal life. Genes regulating cell cycle, differentiation and motility, macromolecule biosynthetic and metabolic process, and angiogenesis are up-regulated in early pregnancy, whereas genes involved in lipid and chemosensory metabolism, stress response, signal transduction and ion transport are highly expressed in term placenta [11].

Another critical time-point in human pregnancy and placental function is the switch from early to mid-gestation. In early pregnancy, normal trophoblast development is the key for successful implantation and formation of maternal-fetal interface that facilitates the dialogue between the two organisms. Mid-gestation placenta supports proportional fetal growth, organ development and fine-scale differentiation, as well as continuing maternal adaptation to pregnancy. Only one published study has compared gene expression differences between first (45–59 days) and second (109–115 days) trimester placentae and reported 61 differentially expressed loci that have been implicated in pregnancy, reproductive physiolology and interaction between organisms [12]. However, the authors were focused on microarray data analysis, which was not followed by any experimental confirmation or biomedical implication.

The present study had three main aims: (i) transcriptome profiling of genes with significant expressional changes in placenta in progression from early to mid-pregnancy, and experimental confirmation of mid-gestation specific gene expression using Taqman qPCR in an extended sample; (ii) investigation of the novel hypothesis that the normal course of late pregnancy may be affected when the genes characteristic to mid-gestation placental transcriptome remain highly expressed until term; (iii) exploring the protein expression of the most prominent identified genes in pregnancy complications, and pilot evaluation of their applicability as biomarkers.

The study reports 154 placental transcripts with significant change in expression levels from gestational weeks 5 to 18. The major advancement in the present study in contrast to earlier publications is the experimental validation of 24 loci from microarray analysis. Furthermore, we confirmed highly significant distinct mid-gestational peaks of gene expression for 10 genes in an extended sample-set of first, second and term placentae (*n* = 43). In support of our study hypothesis, several mid-gestation genes exhibited aberrant placental expression in complicated pregnancies at term, and alterations at the protein level were confirmed for CCNG2 (cyclin-G2), LYPD6 (LY6/PLAUR domain containing protein 6) and STC1 (stanniocalcin-1). Our findings have direct potential for biomedical implications as we demonstrate highly significant elevated concentration of circulating hormone STC1 in the maternal serum in PE patients and especially in the PE subgroup accompanied by affected fetal growth. This warrants further investigations of STC1 as a prognostic biomarker of pregnancy outcome. As an additional novel finding, most of the identified genes specifically up-regulated in mid-gestation placenta have also been implicated in adult complex diseases.

## Materials and Methods

### Ethics Statements

The study was approved by the Ethics Review Committee of Human Research of the University of Tartu, Estonia (permissions no 117/9, 16.06.2003; 146/18, 27.02.2006; 150/33, 18.06.2006; 158/80, 26.03.2007; 180/M-15, 23.03.2009). A written informed consent to participate in the study was obtained from each individual prior to recruitment. All study participants were recruited at the Women’s Clinic of Tartu University Hospital, Estonia in 2003–2011, and were of white European ancestry and living in Estonia.

### Early and Mid-pregnancy Study Group

Placental samples were obtained from females who underwent (a) elective (surgical) termination of pregnancy during first trimester (*n* = 23; gestational age 5–13 weeks, median 8 weeks, 6 days [38–91 days, median 60 days]; median maternal age 27 years, range 18–38 years) or (b) therapeutic medically induced abortion during second trimester due to maternal medical risks of pregnancy, where no fetal anomalies were detected (*n* = 8; gestational age 17–21 weeks, median 18 weeks, 5 days [120–147 days, median 131 days]; median maternal age 23 years, range 18–39 years).

### REPROMETA Sample Collection

REPROgrammed fetal and/or maternal METAbolism (REPROMETA) sample collection harbors clinical data and biological material from singleton pregnancies at term representing gestational weeks 36–42. Information about mother’s diseases, smoking, somatometric data, and childbirth history was obtained from medical records during the course of pregnancy and after birth. Fetal outcome data from delivery included weeks of gestation, birth weight, birth length, head and abdominal circumferences, and placental weight. Cases with documented fetal anomalies, chromosomal abnormalities, families with history of inherited diseases and patients with known pre-existing diabetes mellitus, chronic hypertension and chronic renal disease were excluded. Detailed characteristics of REPROMETA samples for RT-qPCR (*n* = 60) and ELISA (*n* = 129) experiments are given in **Table 1**. The description of the sub-sample used for immunohistochemistry experiments (*n* = 15) is provided in **Table S1**.

**Table 1:** Maternal and offspring characteristics of REPROMETA samples used in the study for RT-qPCR and ELISA experiments.

Mother and offspring characteristics	Control	SGA	LGA	PE	GDM
**A. Placental mRNA expression by RT-qPCR (** ***n*** ** = 60 samples)**					
No of women [nulliparity]	12 [9]	12 [7]	12 [5]	12 [8]	12 [4]
Maternal age (yr)	26.5 (21; 38)	26.5 (18; 34)	26.5 (20; 40)	27.5 (19; 39)	33 (22; 39)*
Maternal height (cm)	166 (152; 175)	167 (163; 171)	168 (156; 180)	170 (156; 178)	161 (150; 173)
Maternal pre-pregnancy weight (kg)	58 (48; 74)	56 (47; 72)	66.5 (55; 85)*	67 (57; 89)*	66.5 (49; 107)
Gestational weight gain (kg)	13 (9; 24)	13 (10; 22)	17 (9; 33)	13 (6; 21.5)	15 (3; 26)
Delivery mode (vaginal/c-section)	10/2	10/2	4/8*	3/9*	4/8*
Smokers during pregnancy (n)	0	1	0	1	0
Gestational age at birth (d)	277 (259; 291)	270.5 (253; 289)	290.5 (284; 292)*	264.5 (253; 287)*	274.5 (253; 293)
Baby’s birth-weight (g)	3574 (2890; 4220)	2580 (2177; 2870)*	4727 (4588; 5010)*	2853 (2178; 4250)*	4054 (3154; 5420)*
Baby’s birth length (cm)	51 (49; 55)	46.5 (42; 49)*	54 (51; 57)*	48 (45; 51)*	52 (48; 55)
Baby’s head circumference (cm)	35 (33; 36.5)	32 (29; 35)*	37 (35.5; 39)*	34 (31.5; 37)	36.5 (34; 38)*
Baby’s abdominal circumference (cm)	34 (33; 36)	30 (28.5; 34)*	37 (36; 40)*	32 (28.5; 37.5)*	36 (32; 40.5)*
Placental weight (g)	520 (390; 750)	452.5 (381; 600)	790 (640; 1050)*	450 (340; 770)	645 (525; 860)*
No of newborns born SGA/LGA	0/0	12/0	0/12	4/0	0/6
No of boys/girls	6/6	5/7	6/6	9/3	5/7
**B. Maternal blood serum analysis of STC1 by ELISA (** ***n*** ** = 129 samples)**					
No of women [nulliparity]	40 [20]	27 [18]	16 [8]	16 [11]	30 [13]
Maternal age (yr)	27 (18; 40)	25 (18; 40)	28 (20; 40)	26 (19; 39)	31 (21; 42)*
Maternal height (cm)	165.5 (152; 175)	166 (153; 173)	167.5 (156; 180)	170 (156; 178)*	165.5 (150; 178)
Maternal pre-pregnancy weight (kg)	58.5 (48; 78)	56 (47; 72)	68 (57; 94)*	68.75 (53; 98)*	69 (46; 122)*
Gestational weight gain (kg)	15 (5; 25)	12.5 (8.5; 22)	17 (9; 33)	14 (6; 26)	14.3 (3; 26)
Delivery mode (vaginal/c-section)	35/5	19/8*	6/10*	5/11*	16/14*
Smokers during pregnancy (n)	1	4	0	1	0
Gestational age at birth (d)	281 (259; 291)	271 (253; 289)*	285.5 (267; 292)*	265 (253; 287)*	276 (253; 293)
Baby’s birth-weight (g)	3626 (2722; 4270)	2580 (2004; 2992)*	4717 (4364; 5050)*	2888 (2170; 4250)*	4065 (2934; 4964)*
Baby’s birth length (cm)	51 (48; 55)	47 (42; 49)*	53.5 (51; 57)*	48 (45; 51)*	51 (48; 54)
Placental weight (g)	550 (370; 800)	440 (200; 650)*	780 (640; 970)*	450 (340; 770)	635 (410; 1060)
No of newborns born SGA/LGA	0/0	27/0	0/16	7/0	0/12
No of boys/girls	19/21	7/20	9/7	9/7	13/17

Data are given as medians with ranges, except where indicated differently.

Nulliparity  =  no previous childbirth.

* *P*<0.05 *vs*. control group, Mann-Whitney U or Fisher’s exact test.

SGA, small-for-gestational age; LGA, large-for-gestational age; PE, preeclampsia; GDM, gestational diabetes mellitus; yr, years; d, days.

The REPROMETA participants were stratified in clinical subgroups based on the birth weight of a newborn and the absence/presence of maternal pregnancy-specific complications. The control group comprised of uncomplicated pregnancies resulting in the birth of newborn with the weight appropriate-for-gestational age (AGA, birth-weight between 10–90 percentile; *n* = 40; median gestational age 281 days, range 259–291 days; median maternal age 27 years, range 18–40 years). Study groups of disturbed fetal growth comprised of newborns born as (i) small-for-gestational age (SGA, <10^th^ percentile; *n* = 27; median gestational age 271, range 253–289 days; median maternal age 25 years, range 18–40 years) and (ii) large-for-gestational age (LGA, >90^th^ percentile; *n* = 16; median gestational age 285.5, range 267–292 days; median maternal age 28 years, range 20–40 years). The weight percentiles for defining SGA and LGA were calculated on the basis of data from Estonian Medical Birth Registry [13].

Study groups of maternal pregnancy complications included maternal (i) preeclampsia (PE; *n* = 16; median gestational age 265, range 253–287 days; median maternal age 26 years, range 19–39 years) and (ii) gestational diabetes mellitus (GDM; *n* = 30; gestational age of median 276, range 253–293 days; median maternal age 31 years, range 21–42 years). Seven newborns in PE group were additionally classified as SGA and twelve newborns in GDM group as LGA. All PE cases represented the severe form of late-onset preeclamptic pregnancies and were defined as hypertensive (systolic blood pressure ≥160 mmHg and/or diastolic blood pressure ≥110 mmHg) and/or had proteinuria of ≥5 g in 24 hours. GDM was diagnosed when 75 g oral glucose tolerance test (OGTT) performed at 24–28 weeks of gestation revealed either a fasting venous plasma glucose level of >4.8 mmol/l, and/or at 1 h and 2 h plasma glucose level of >10 mmol/l and >8.7 mmol/l glucose, respectively.

### RNA Extraction

Full-thickness blocks of 1–3 cm were taken from a middle region of mid-gestation and term placenta within 2 h after medically induced abortion, caesarean section or vaginal delivery. The sampled full-thickness blocks involved all placental layers. First trimester samples were obtained after elective (surgical) termination of pregnancy. Collected tissue samples were snap-frozen in liquid nitrogen and stored at −80°C or placed immediately into RNAlater solution (Ambion Inc, Austin TX) and kept at −20°C until RNA isolation.

Total RNA was extracted from 200–300 mg of homogenized placental tissue containing various placental cell types using TRIzol reagent (Invitrogen, Carlsbad, CA) and purified with NucleoSpin® II Isolation Kit (Macherey-Nagel GmbH & Co. KG, Düren, Germany) according to the manufacturers’ protocols. Purity level and concentration of isolated total RNA was measured using NanoDrop® ND-1000 UV-Vis spectrophotometer (NanoDrop Technologies, Inc., Wilmington, DE, USA).

### Microarray Hybridization

One microgram of total RNA was reverse transcribed to cDNA (SuperScript™ III First Strand Synthesis SuperMix kit, Invitrogen, Carlsbad, CA) and subjected to microarray analysis. Expression of ∼47,000 transcripts in second (4 samples) and first trimester (6 samples) placentae was addressed using Affymetrix Human Genome U133 plus 2.0 GeneChip microarrays following manufacturer’s instructions. Briefly, complementary RNA (cRNA) was synthesized using cDNA templates by *in vitro* transcription reaction and labeled with biotin in the presence of T7 RNA Polymerase and biotinylated nucleotide/ribonucleotide mix. Biotin-labeled cRNA probes were purified, fragmented and hybridized to GeneChip expression arrays.

### Statistical Analysis of Microarray Data

Microarray data of analyzed placental samples is MIAME compliant and the raw datasets have been deposited to a MIAME compliant database, the Gene Expression Omnibus (GEO) data repository (early pregnancy, *n* = 6, accession no GSE22490; mid-pregnancy, *n* = 4, accession no GSE37901). Quality control (QC), pre-processing and linear modeling were performed using R-based Bioconductor packages *affy* and *limma* [14] (**Figure S1**). GeneChip CEL-files were imported to dChip [15] and analyzed using perfect match-mismatch (PM/MM) modeling and invariant set normalization. The genes with high signal intensity were filtered (dChip signal threshold = 100).

The expression value of each gene was modeled as the linear function of gestational age (duration of gestation) of associated samples (*n* = 10; gestational days 38, 55, 2×56, 81, 91, 120, 121, 126, 132). Statistical significance of the time-dependent gene expression model was evaluated with an ANOVA (analysis of variance) test that comparatively evaluated the variance in a null, time-independent model of gene expression. To lower the probability of apparently significant results arising from repeated statistical tests for thousands of genes, Benjamini-Hochberg False Discovery Rate (FDR) multiple testing corrections were used [16]. Additionally, empirical Bayes moderated t-test with FDR correction was used for group comparison between first (*n* = 6; gestational days 38, 55, 2×56, 81, 91) and second (*n* = 4; gestational days 120, 121, 126, 132) trimester samples (details are provided in **Text S1**).

Functional enrichment analysis of significantly differentially expressed genes was carried out with g:Profiler software [17], using default settings except for those described below (g:Profiler with data from Ensembl 65). Analysis was carried out separately for up-regulated genes and down-regulated genes. The background gene set for statistical testing included all genes on the Affymetrix Human Genome U133 plus 2.0 GeneChip platform. Benjamini-Hochberg (FDR) method was used for correcting enrichment *P*-values for multiple testing [16]. Computationally predicted functional evidence from Transfac, BioGrid and MirBase datasets was filtered from final results.

### Reverse Transcription Quantitative PCR (RT-qPCR)

Taqman RT-qPCR assays were performed for 14 genes with significant *P*-values (ANOVA, FDR-corrected *P*<0.05) from dynamic gestation-age dependent microarray analysis; for two genes (*GMPPB*, GDP-mannose pyrophosphorylase B; *LOC131185*, RAD23 homolog B (*S. cerevisiae*) pseudogene) TaqMan assays were not available (**Table S2**). Taqman RT-qPCR experiments were performed for additional ten genes with suggestive *P*-values in microarray dataset (ANOVA, FDR-corrected 0.05≤*P*<0.1): (i) previously described placental transcriptional dynamics (*CDH11*, cadherin 11; *STC1*; *CCNG2*; *NEDD9,* neural precursor cell expressed developmentally down-regulated 9; *ZFP36L1*, zinc finger protein 36, C3H type-like 1) [11], [12]; (ii) mammalian imprinted genes (*PLAGL1,* pleiomorphic adenoma gene-like 1; *MEG3,* maternally expressed 3; *GATM*, glycine amidinotransferase) [18]–[20]; (iii) lowest borderline *P*-value (*SNX18*, sorting nexin 18; *P* = 0.0502) or gene-probe representing highest expressional change (*BMP5*, bone morphogenetic protein 5; fold change 58.89) (**Table S2**). In total, mRNA level of 24 genes (**Table S3**) was assessed by Taqman RT-qPCR in samples from uncomplicated early (*n* = 23), mid- (*n* = 8) and term pregnancies (*n* = 12). Expression of the identified 16 mid-gestation specific loci was further analyzed in REPROMETA placental samples representing pregnancies with affected fetal growth (SGA, LGA) or maternal complications (PE and GDM).

Quantitative gene expression was assessed by biplex qPCR of target sequence and housekeeping gene *HPRT1* (hypoxanthine phosphoribosyltransferase 1) as a reference (assay ID: 4326321E, Applied Biosystems, Foster City, CA, USA). All qPCR reactions were performed in triplicate in 384 micro-well plates in ABI 7900HT Real-time PCR system (Applied Biosystems, Foster City, CA, USA) using HOT FIREPol® Probe qPCR Mix (Solis BioDyne, Tartu, Estonia) and commercially available premade TaqMan Gene Expression Assays (Applied biosystems, Foster City, CA, USA) for 24 tested and the one reference gene *HPRT1* (**Table S2**). Negative controls contained either RNA that was not reverse transcribed or lacked template inputs. RT-qPCR reactions were initially denaturated at 95°C for 15 min, followed by 40 cycles of 15 s at 95°C and 1 min at 60°C. Relative quantification was determined by using the standard curve method. In our experiments, the reference gene *HPRT1* was expressed at approximately the same level as the selected genes of this study.

### Statistical Analysis of RT-qPCR Data

Relative mRNA expression values were determined by comparative C_T_ method, that accounted for mean values of normalized expression calculated by averaging three independently measured normalized expression values of the triplicate [21]. Q-Gene software was used for calculations and efficiency corrections (BioTechniques, Carlsbad, CA, USA).

The statistical analyses were performed using statistical package R version 2.9.0. First, ANOVA F-test was used to estimate the linear function of gestational age from early to mid-gestation samples (*n* = 31; gestational days 38 to 147). Second, significance of RT-qPCR measurements among the placental samples representing first (*n* = 23), second (*n* = 8) and third trimester (*n* = 12) pregnancies was assessed by Student t-test (no adjustment with covariates). Genes with significant results in t-tests were further studied in covariate analysis. Analysis of covariance (ANCOVA) was applied to incorporate confounding effects between REPROMETA control group of uncomplicated pregnancies and the four study groups of pregnancy complications (SGA, LGA, PE, and GDM). First, individual confounding effects were discovered through statistical analysis of control and patient groups. Mann-Whitney U-test was used to compare continuous phenotypic covariates, and Fisher’s exact test was applied to compare categorical covariates. Second, significant confounding effects were studied with ANCOVA analysis independently for each complication group, by comparing null and alternative models of predicting gene expression values. The null model contained a linear combination of all selected confounding covariates as predictors, and the alternative model contained an additional factor term reflecting the disease states of the samples (normal vs. complicated). The difference in fits of null and alternative models was quantified by the F-test, reflecting the importance of disease states in association to gene expression and related confounding variables. ANCOVA tests for all groups were adjusted by gestation age, placenta weight, infant gender and type of delivery. Differential expression testing in GDM group was additionally adjusted by infant weight and maternal age, and in PE group with infant weight. FDR correction for multiple testing was used separately for each complication group to reduce the number of false positive associations. Results with *P*-values *P*≤0.05 were considered significant.

### Measurement of STC1 Protein Expression in Maternal Plasma by ELISA and Statistical Analysis

Plasma samples collected on the day of delivery were analyzed for STC1 protein levels in 83 REPROMETA study participants including the controls of uncomplicated pregnancies (*n* = 40) and the four patient groups with fetal (SGA, *n* = 27; LGA, *n* = 16) or maternal (PE, *n* = 16, GDM, *n* = 30) pregnancy complications (**Table 1**). STC1 protein expression was measured using DuoSet ELISA development kit (#DY2958, lot #1215530; R&D Systems Europe, Ltd., Abingdon, UK) following manufacturer’s instructions. Experimental details are given in **Text S2**.

Statistical analysis of STC1 protein expression levels was carried out similarly to RT-qPCR analysis described above, except that different sets of covariates were used in ANCOVA analysis. Tests for all complication groups were adjusted by the type of delivery and maternal weight. In the SGA and LGA study groups, tests addressing differential plasma levels of STC1 were additionally adjusted by gestational age. In the GDM study group, tests were corrected for the infant weight and maternal age, and in the PE group for infant weight, gestational age and maternal height. FDR multiple testing correction was used for all tests. Results with *P*-values *P*≤0.05 were considered significant.

### Immunohistochemical Localization of LYPD6 and CCNG2 Proteins

Immunohistochemical (IHC) staining was performed using 6 µm placental paraffin sections sampled from five control, five preeclamptic (PE) and five gestational diabetes mellitus (GDM) pregnancies at term (**Table S1**). Placental tissue samples used for IHC staining originated from the same tissue block as samples collected for RNA extractions. Anti-human LYPD6 antibody (1∶30, LS-C102542, LifeSpan BioSciences, Seattle, USA) and Dako REAL™ EnVision Detection System kit (DakoCytomation, Denmark) were used for LYPD6 IHC staining. Primary anti-human CCNG2 (Santa Cruz Biotechnology, sc-7266, 1∶100) and secondary biotinylated polyclonal rabbit anti-goat antibodies (DakoCytomation, Denmark, E 0466, 1∶400) and LSAB2 System-HRP kit (DakoCytomation, Denmark) were used for CCNG2 IHC staining. All IHC stainings were conducted following manufacturer’s instructions; details are provided in **Text S3**. Standard hematoxylin-eosin staining was used to describe histological features characteristic to normal, PE and GDM placentas at term. Imaging was performed with the Olympus BX60 microscope using Olympus DP71 digital camera and CellA imaging software (Olympus Optical). Microscope magnifications ×100 and ×400 were used. All measurements were acquired at the same light intensity and processed consistently. The IHC staining pattern of LYPD6 and CCNG2 proteins in the placental sections neither from control, PE nor GDM pregnancies depended on the mode of delivery.

### Accession Numbers for Microarray Data

[GSE22490, GSE37901].

## Results

### Temporal Dynamics of Gene Expression in Placenta from Early to Mid-pregnancy

**Figure 1:**
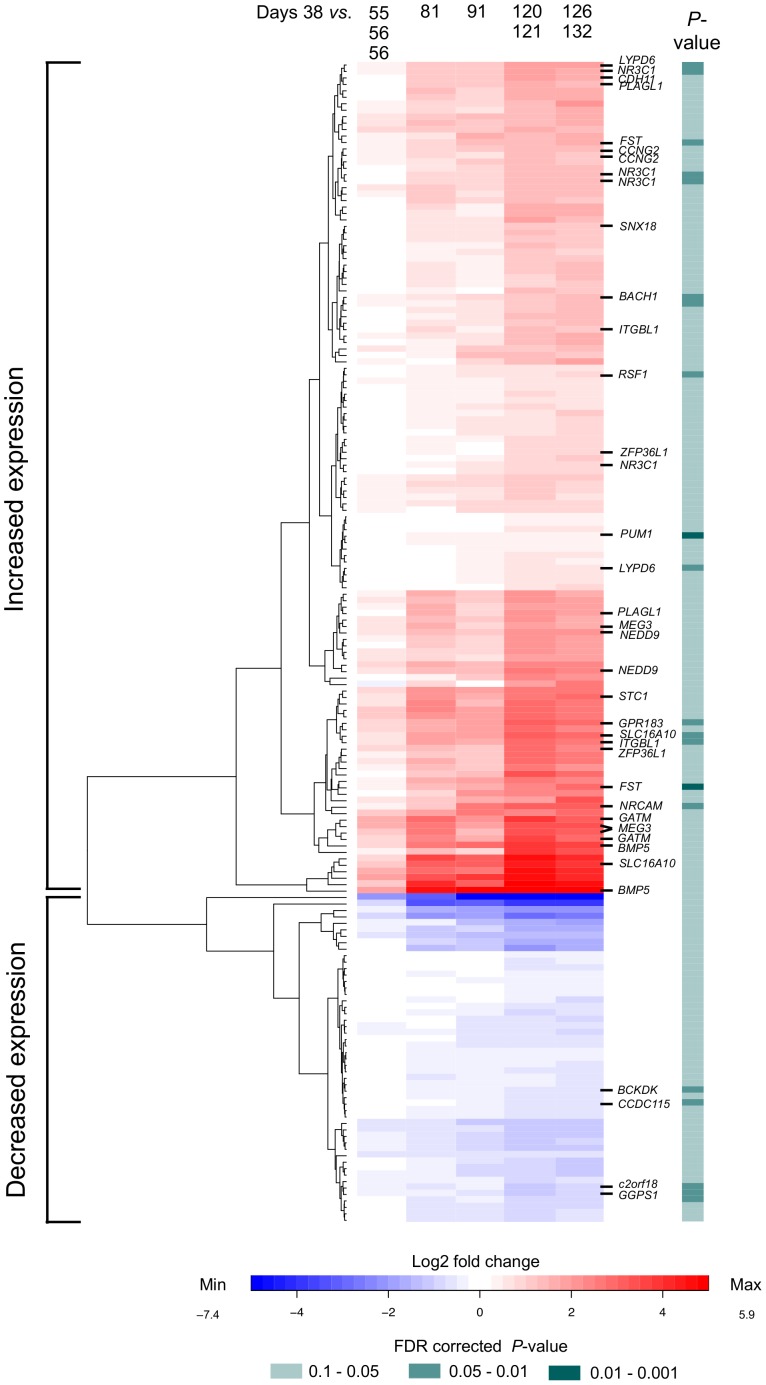
Placental genes with considerable expressional change in progression from early to mid-pregnancy. Ten placental tissue samples subjected to dynamic linear transcriptome expression profiling (Affymetrix HG-U133 plus 2.0 GeneChips) represented gestatational weeks of 5 (38 days), 8 (days 55, 56, 56), 11 (81 days), 13 (91 days), 17 (days 120, 121) and 18 (days 126, 132). The heatmap shows 154 genes (180 probe-sets) with significant gradual increase (red) or decrease (blue) in placental transcription in first and second trimester placentas (ANOVA, FDR corrected *P*<0.1). Color intensity reflects mean log2 fold change in gene expression with one first trimester sample (week 5; 38 gestational days) as reference. For the placental samples collected at close gestational age, log2 fold change calculations represent the median values of 2–3 samples (55/56/56; 120/121; 126/132 gestational days). Hierarchical clustering with Euclidean distance, visualized by the dendrogram on the left, clearly separated genes with increased and decreased expression. Colorstrip on the right of the heatmap highlights genes with strongest statistical significance of differential expression. A subset 24 genes selected for further experiments are labeled on the vertical axis.

Transcriptome profiling of ten placental tissue samples collected at gestational ages from week 5 to 18 [gestational days: 38, 55, 2×56, 81, 91, 120, 121, 126, 132] was conducted using GeneChip (Affymetrix®; ∼47,000 transcripts) expression arrays. Quantitative microarray analysis of gene expression dynamics across the studied gestation period identified 180 placental transcripts (representing 154 genes) with significant change in expression levels (ANOVA, FDR corrected *P*<0.1, *n* = 10 samples), including 105 genes with gradually increasing and 49 genes with decreasing transcript levels (**Figure 1** **,** **Table S2**).

**Figure 2:**
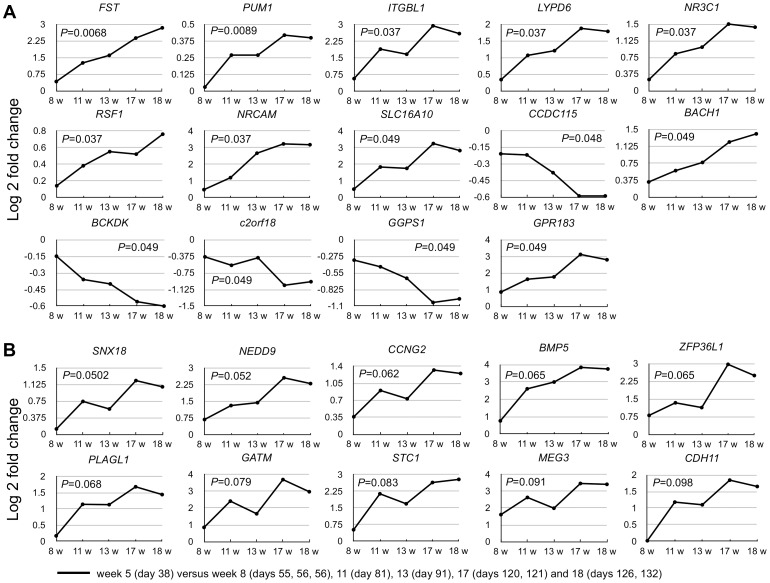
Temporal quantitative gene expression changes in discovery samples over gestational weeks 5–18. Log2 fold changes between Affymetrix GeneChip transcription values were calculated to estimate the direction of gene expression. Gene expression level at week 5 (gestational day 38) were used as a baseline. For the placental samples collected at close gestational age, fold change calculations represent the median values of 2–3 samples (week 8: 55/56/56 gestational days; week: 17; 120/121 gestational days; week 18: 126/132 gestational days). (A) 14 genes with significant change in transcription (ANOVA, FDR corrected *P*<0.05, *n* = 10) and (B) additional 10 genes with known effect on pregnancy that showed mildly significant differential expression (FDR corrected *P*<0.1) were selected for subsequent RT-qPCR analysis (**Table S2**). Genes are ordered by decreasing *P*-value.

The most significant increase in expression was detected for the *FST* (follistatin) gene (FDR *P* = 0.0068; fold change, fc = 7.16; **Figure 2A**). Significant (FDR *P*<0.05) over 6-fold increase in placental expression was also identified for *NRCAM* (neuronal cell adhesion molecule; FDR *P* = 0.037; fc = 9.06), *SLC16A10* (solute carrier family 16 member 10; FDR *P* = 0.049; fc = 7.11), *GPR183* (G protein-coupled receptor 183; FDR *P* = 0.049; fc = 6.96) and *ITGBL1* (integrin, beta-like 1; FDR *P* = 0.037; fc = 6.06). Among the genes with borderline statistical evidence (FDR 0.05≤*P*<0.1) for increasing placental expression and with known effect on fetal development, *BMP5* (two Affymetrix probesets: fc = 58.89; 13.36), *GATM* (fc = 9.32; 7.84), *STC1* (fc = 6.92; 2.57) and *MEG3* (three probesets: fc = 10.63; 8.28; 3.14) stood out with considerable change in transcript levels (**Figure 2B** **; Table S2**). The multiple Affymetrix probe sets targeting *BMP5* and *MEG3* hybridize to different splice variants of the gene (**Figure S2**). Alternative *MEG3*-transcribed non-coding RNA-s have been implicated in tissue-specific expression patterns [22]. The genes highlighted in the GeneChip analysis are also similar according to the hierarchical clustering of dynamic expression profiles across gestational weeks 5 to 18 (**Figure 1**). Full names and descriptions of all relevant genes are provided in **Table 2** and **Table S3**.

**Table 2:** Molecular functions of identified mid-gestation marker genes in placenta and their involvement in clinical conditions.

Short name	Full name	Biological function^a^	Related disease or clinical condition^a^
*BMP5*	*Bone morphogenetic* *protein 5*	Bone, cartilage and limb development,skeletal growth	Axial skeletal abnormalities, rheumatoid arthritis, osteoarthritis, hypertensive nephrosclerosis; heterotopic ossification; pancreatic, prostate and breast cancer
*CCNG2*	*Cyclin-G2*	Negative regulation of cell cycle, adipogenesis; proliferation and differentiation of uterinecells in implantation and decidualization	Thyroid carcinoma, gastric, oral, breast and ovarian cancer
*CDH11*	*Cadherin-11*	Cell adhesion, bone formation, growth,maintenance and morphology, tumor suppressor	Osteoarthritis, osteosarcoma, glioblastoma, retinoblastoma, metastasis of prostate and breast cancers, pulmonary fibrosis
*FST*	*Follistatin*	Inhibition of FSH release, folliculogenesis, bone mineralization, muscle growth	Polycystic ovary syndrome, fertility, recurrent miscarriage, metastasis of prostate cancer, osteoarthritis
*GATM*	*Glycine amidino-transferase*	Creatine biosynthesis, kidney function, nervous system development	Heart failure, chronic kidney disease, mental retardation
*GPR183*	*G-protein coupled* *receptor 183*	Humoral immunity	Type 1 diabetes
*ITGBL1*	*Integrin beta-like 1*	Cell adhesion	Growth hormone deficiency
*LYPD6*	*LY6/PLAUR domain* *containing 6*	Transcriptional regulation; inhibition of tumor promotion	Location within microduplication region linked to developmental delay and autistic features
*MEG3*	*Maternally expressed* *gene 3* [imprinted]	Non-coding RNA, negative regulation of cell proliferation, embryonic development, tumor suppressor	Type 1 diabetes, pituitary tumor, meningioma, acute myeloid leukemia; imprinting defects affect embryonic development
*NEDD9*	*Neural precursor cell* *expressed developmentally* *down-regulated 9*	Tyrosine-kinase-based signaling related to cell adhesion, proliferation	Alzheimer’s and Parkinson’s disease; melanoma, head and neck squamous cell carcinoma, glioblastoma, colon cancer
*NR3C1 (GR)*	*Nuclear receptor subfamily* *3 group C member 1*(glucocorticoid receptor)	Transcriptional regulation, chromatinremodeling, cellular proliferation anddifferentiation, inflammatory response	Generalized glucocorticoid resistance; coronary heart disease; rheumatic diseases; obesity; asthma, colorectal cancer, acute lymphoblastic leukemia, adrenocortical carcinoma
*NRCAM*	*Neuronal cell adhesion* *molecule*	Nervous system development, cell adhesion	Autism, addiction; melanoma, colon pancreatic and papillary thyroid cancer
*PLAGL1*	*Pleiomorphic adenoma* *gene-like 1* [imprinted,paternally expressed]	Transcription factor, apoptosis, embryonicdevelopment and growth; cell cycle arrest,cardiac morphogenesis, development of pancreas	Imprinting defects affect embryonic development; overexpression in fetal development leads to transient neonatal diabetes mellitus; ovarian, breast and gastric cancer, melanoma, astrocytoma, pancreatic adenocarcinoma, renal cell carcinomas, capillary hemangioblastoma, pituitary adenoma, B-cell non-Hodgkin's lymphomas
*SLC16A10*	*Solute carrier family 16 member 10*	Aromatic amino acids transport,thyroid hormone transport	Intrauterine growth restriction
*STC1*	*Stanniocalcin 1*	Renal and intestinal Ca2+/P homeostasis, boneand muscle development, kidney function,gestational and nursing state regulator	Chronic kidney disease, heart failure; colorectal, ovarian, hepatocellular and breast cancer, squamous cell carcinoma; reduced postnatal growth, affected female reproductive potential; overexpression in mice leads to dwarfism and increased metabolic rate
*ZFP36L1*	*Zinc finger protein 36* *C3H type-like 1 (butyrate* *response factor-1)*	regulation of translation; RNA metabolicprocess; regulation of mRNA stabilityand decay	Acute myelogenous leukemia, T-cell leukemia/lymphoma, breast cancer; lack of expression in midgestation results in abnormal placentation and fetal death

a References are listed in **Table S9**.

Alternatively, microarray data were analyzed in two groups of early (*n* = 6) and mid-gestation (*n* = 4) samples. As a result, 205 genes with significantly increased expression and 24 genes with significantly decreased expression levels were detected (empirical Bayes moderated t-test, FDR *P*<0.1; **Figure S3, Table S4**). The group-based analysis recovered 63 (60%) significantly up-regulated genes from the first ANOVA-based analysis of dynamic gene expression, confirming the validity of both statistical approaches and highlighting additional genes with alternative modes of expression. Detailed results of the group-based analysis are provided in **Text S1**, **Figure S3**, **Table S4** and **Table S5**.

Genes for the further experimental confirmation and replication by Taqman RT-qPCR were selected from top candidates of gestation-age-dependent dynamic analysis (ANOVA). Out of 24 genes selected for RT-qPCR validation, 17 genes were also significant in group-based analysis.

### Gene Ontology and Pathway Analysis of Differentially Expressed Genes

Functional enrichment analysis with g:Profiler software [17] revealed more than 200 Gene Ontology (GO) categories and pathways that are statistically over-represented in the list of genes with dynamically increasing transcript levels. Many broad biological processes (GO category type BP) are characteristic to evolving placenta in pregnancy progression such as ‘cell communication’ (*n* = 42 genes, FDR *P* = 1.26×10^−5^, hypergeometric test) and ‘multicellular organism development’ (*n* = 37 genes, FDR *P* = 1.7×10^−5^), ‘cellular response to stimulus’ (*n* = 43, FDR *P* = 1.9×10^−5^) and ‘cell surface receptor signaling’ (*n* = 26, FDR *P* = 6.5×10^−5^), ‘cell adhesion’ (*n* = 15, FDR *P* = 1.9×10^−4^) and ‘migration’ (*n* = 11, FDR *P* = 0.011), ‘anatomical structure development’ (*n = *33, FDR *P* = 4.3×10^−4^) and ‘growth’ (*n* = 12, FDR *P* = 2.4×10^−3^) (**Table S6**). The analysis also highlights several specific processes related to pregnancy development and maintenance, such as ‘blood vessel development’ (*n = *9, FDR *P* = 4.8×10^−3^), ‘VEGF receptor signaling’ (*n = *4, FDR *P* = 7.7×10^−4^), ‘gonadotropin secretion’ (*n* = 2, FDR *P* = 0.016) and ‘superoxide metabolic processes’ (*n = *3, FDR *P* = 0.014). In contrast to high number of processes and pathways enriched among the genes with dynamically increasing transcript levels from early to mid-pregnancy, substantially fewer biological processes represent the list of genes with decreasing expression. The most significant enrichment was detected for genes related to ‘transcription from RNA polymerase III promoter’ (FDR *P* = 1.7×10^−4^), although all four identified genes from this pathway exhibit modest decrease in transcript levels (fc = 0.7–0.75; **Table S2**).

### Identification of Mid-pregnancy Specific Placental Genes

In total, 24 genes were selected for RT-qPCR analysis of placental samples representing three gestational trimesters (**Figure 2**; **Table S3**). The analysis included 14 genes with highly significant gradual expressional change in placenta from early to mid-pregnancy (ANOVA analysis of microarray data, FDR *P*<0.05; **Table S2**). RT-qPCR experiments included additional ten genes with mildly significant evidence of transcriptional increase (ANOVA FDR 0.05≤*P*<0.098) in pregnancy progression towards second trimester (details in **Materials and Methods**). To confirm the differential expression between the first and the second trimester placentae of the selected genes based on microarray analysis, we used an expanded set of early (*n* = 23, median gestational age 8 weeks, 6 days) and mid-pregnancy samples (*n* = 8, median gestational age 18 weeks, 5 days; **Materials and Methods**). Expressional change was tested using both, dynamic (ANOVA) and group-based (Student t-test) statistical approaches. In order to follow the expressional dynamics of mid-gestation genes throughout the course of pregnancy, the expression levels were determined additionally in term placentas from uncomplicated pregnancies (Controls, *n* = 12; median gestational age 40 weeks, 1 day; **Table 1A**).

**Figure 3:**
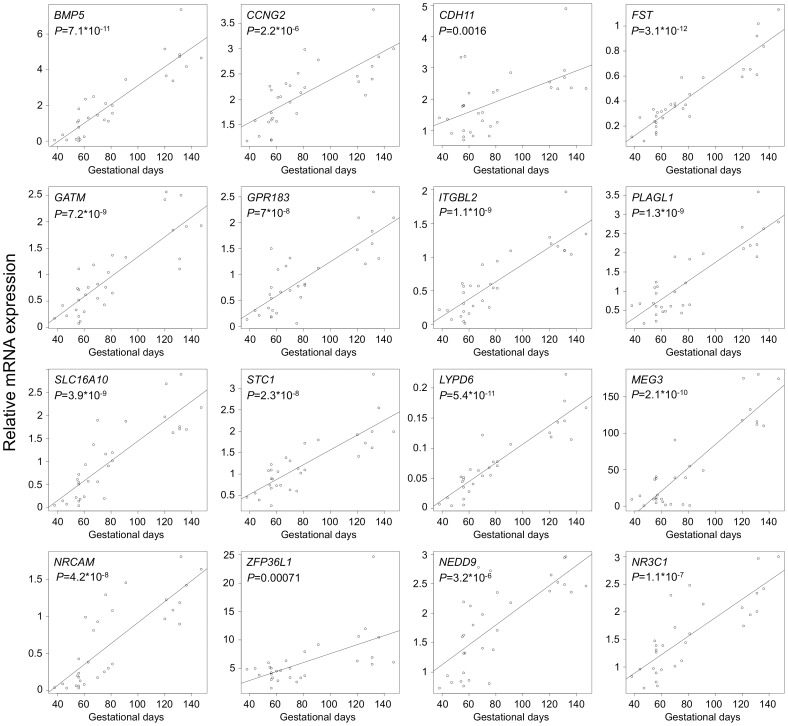
Genes with significant dynamic increase in expression during early and mid-gestation as experimentally confirmed by RT-qPCR. Relative mRNA expression levels in the extended sample set of first and second trimester placentas (*n* = 31; from gestational days 38 to 147) were determined by TaqMan assays. *P-*values were calculated by ANOVA and subjected to multiple testing correction (FDR). Genes below the significance threshold of *P*-value>0.02 are shown in **Figure S4**.

**Figure 4:**
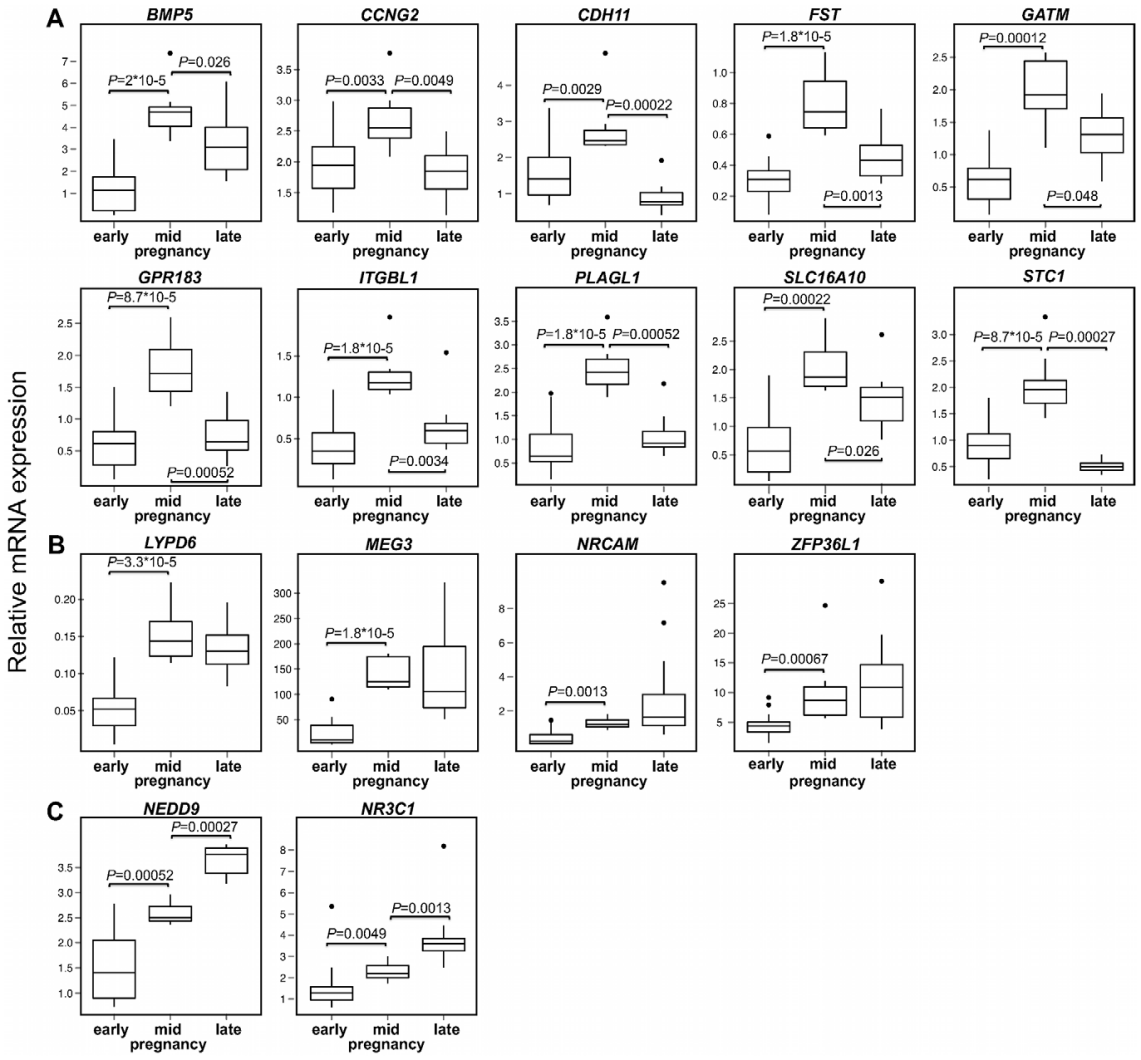
Expressional dynamics of identified mid-pregnancy specific genes in placenta from early to term gestation. Relative mRNA levels were determined by RT-qPCR TaqMan assays in placental tissues from early- (5–13 gestational weeks; *n* = 23), mid- (17–21 gestational weeks; *n* = 8) and term-gestation (36–41 gestational weeks; *n* = 12) samples of uncomplicated pregnancy cases. Boxplots show mid-gestation marker genes with (A) significantly increased mRNA expression compared to early- or late-gestation placental samples, (B) significantly increased expression levels compared to early gestation placental samples, and (C) gradual increase in expression during pregnancy. *P*-values were calculated by Student t-test and subjected to multiple testing correction (FDR) (**Table S7**).

Among the 24 tested genes, 16 were confirmed with significant differential expression between the first and the second trimester placenta (ANOVA and t-test, FDR *P*<0.005; **Figure 3**; **Figure 4**; **Table S7**). The most significant increase in gene expression in mid-gestation compared to early pregnancy was detected for *FST*, *MEG3*, *PLAGL1, ITGBL1, BMP5* and *STC1* known to contribute to fetal development and growth, as well as for humoral immunity related *GPR183* and transcriptional regulator *LYPD6* (ANOVA, FDR *P*≤7×10^−8^; t-test, FDR *P*≤8.7×10^−5^; fc = 2.21–6.36; **Figure 3**; **Figure 4**; **Table 2** **;** **Table S7**). Additionally, the analysis revealed a significant activation of *CDH11*, *SLC16A10, NRCAM* and *GATM* also regulating embryonic development and growth, as well as *ZFP36L1*, *CCNG2*, *NEDD9, NR3C1* (nuclear receptor subfamily 3, group C, member 1) involved in cell cycle regulation, proliferation and RNA metabolism, such as (ANOVA, FDR *P*≤0.0016; t-test, *P*<5×10^−3^; fc = 1.41–2.99) (**Figure 3**; **Figure 4**; **Table 2** **;** **Table S7**).

Ten genes (*BMP5*, *CCNG2*, *CDH11*, *FST*, *GATM*, *GPR183*, *ITGBL1*, *PLAGL1*, *SLC16A10*, *STC1*) showed a clear peak of placental gene expression at mid-gestation, followed by a 1.4 to 4.2-fold decrease in transcript levels at term (t-test, FDR *P*<0.05; **Figure 4A** **, Table S7**). The most drastic drop in gene expression characterizes *STC1* and *CDH11* (FDR *P*<0.0003). The expression of four genes (*LYPD6*, *MEG3*, *NRCAM*, *ZFP36L1*) was maintained at the mid-gestation level until delivery (**Figure 4B**). Transcript levels of *NEDD9* and *NR3C1* continued gradual increase until the end of gestation (FDR corrected *P*<0.002; fold changes 1.4 and 1.68, respectively; **Figure 4C**).

### Increased Placental Expression of Mid-gestation Genes in Pregnancy Complications

We hypothesized that the normal course of late pregnancy may be affected when the genes characteristic to mid-gestation placental transcriptome remain highly expressed until term. The hypothesis is based on the assumption that late pregnancy complications may be accompanied by either of the two scenarios: insufficient down-regulation of mid-gestation specific genes that would normally undergo rapid inhibition at term, or continued transcriptional up-regulation of genes that would normally reach their expressional plateau already in mid-pregnancy. This hypothesis was tested by comparing the gene expression of the identified mid-term specific genes in term placentas of normal and complicated pregnancies representing affected fetal growth (small- and large-for-gestational-age newborns; SGA, LGA) or maternal pregnancy disturbances (preeclampsia, PE; gestational diabetes mellitus, GDM) (**Table 1A** **,** **Table 2** **,** **Figure 3** **,** **Figure 4** **)**.

Among the tested genes, seven loci exhibited significantly higher placental expression in pregnancy complications compared to the control group (t-test; **Table 3**). Three genes (*STC1, CCNG2, LYPD6*) maintained significant differential expression in more than one pregnancy complications also after FDR correction and adjustment for multiple confounding factors (details of ANCOVA analysis are provided in **Materials and Methods**). *STC1* with the sharp placental expression peak in mid-gestation (**Figure 4A**) showed significantly higher expression in term placentae of all studied pregnancy complications compared to normal gestation. In the placentae of preeclamptic mothers (t-test, *P* = 2.6×10^−4^; fc = 1.51; **Table 3**) and in the SGA group (t-test *P* = 6.6×10^−4^; fc = 1.74) the transcript level of *STC1* compared to control placentae remained significantly increased after correction for multiple testing (FDR *P*<0.05). Increased placental expression of *STC1* was also significantly associated with the SGA group when all relevant confounding factors were considered (ANCOVA, FDR *P*<0.05; **Table 3**). *STC1* encodes a glycoprotein hormone regulating renal and bone development and metabolism [23]. Higher expression of *STC1* was also detected in the LGA (*P* = 0.0082; fc = 1.41, t-test) and the maternal GDM groups (*P* = 0.021; fc = 2.54), but these observations did not remain significant after correction for multiple testing and confounding effects (ANCOVA; **Table 3**). Cell cycle inhibitor *CCNG2* showed significantly increased expression in placentas obtained from pregnancies complicated by maternal PE (t-test *P* = 4.3×10^−4^; FDR *P*<0.05; fc = 1.49) or GDM (*P* = 5.7×10^−4^; FDR *P*<0.05; fc = 1.46), and also from deliveries resulted in the birth of LGA babies (*P* = 0.027, fc = 1.23). This observation was significant also when confounding effects were considered (ANCOVA, FDR *P*<0.05; **Table 3**). Significantly higher placental mRNA level of the transcription regulator *LYPD6* [24] was identified in all patient groups: GDM (t-test: *P* = 1.3×10^−5^; FDR *P*<0.05; fc = 1.79), PE (*P* = 0.0024; FDR *P*<0.05; fc = 1.38), LGA (*P* = 0.0095; fc = 1.5) and SGA (*P* = 0.014; fc = 1.57). In the SGA group, differential placental expression of LYPD6 maintained statistical significance after correcting for confounding effects (ANCOVA, FDR *P*<0.05; **Table 3**).

**Table 3:** Mid-gestation marker genes with increased mRNA expression levels in term placenta of pregnancy complications compared to placentas of uncomplicated gestations^a^.

		Differential placental mRNA expression				
		Student t-test^c^		ANCOVA^c^		
Gene	Pregnancy complication^b^	*P*-value	FDR-corrected*P*-value	*P* -value	FDR-corrected*P*-value	Fold change^d^
*STC1*	PE	**0.00026**	**0.014**	0.47	0.89	1.51
	GDM	**0.021**	0.12	**0.022**	0.06	2.54
	SGA	**0.00066**	**0.014**	**0.00077**	**0.0054**	1.74
	LGA	**0.0082**	0.087	0.053	0.34	1.41
*CCNG2*	PE	**0.00043**	**0.014**	**0.0019**	**0.013**	1.46
	GDM	**0.00057**	**0.014**	**0.00019**	**0.0013**	1.49
	SGA	0.1	0.28	0.081	0.12	1.21
	LGA	**0.027**	0.12	0.14	0.34	1.23
*LYPD6*	PE	**0.0024**	**0.035**	0.15	0.52	1.38
	GDM	**0.000013**	**0.0017**	**0.026**	0.06	1.79
	SGA	**0.014**	0.1	**0.0075**	**0.026**	1.57
	LGA	**0.0095**	0.087	0.41	0.48	1.5
*GATM*	PE	**0.025**	0.12	0.87	0.97	1.41
	GDM	**0.024**	0.12	**0.041**	0.072	2.08
	SGA	**0.022**	0.12	**0.043**	0.08	1.49
	LGA	**0.019**	0.11	0.14	0.34	1.44
*GPR183*	PE	0.15	0.36	0.77	0.97	1.29
	GDM	0.1	0.28	0.088	0.1	1.41
	SGA	**0.025**	0.12	0.057	0.08	1.52
	LGA	**0.029**	0.12	0.21	0.34	1.41
*MEG3*	PE	0.13	0.33	0.97	0.97	1.42
	GDM	**0.012**	0.1	0.26	0.26	2.13
	SGA	0.067	0.22	0.054	0.08	3.13
	LGA	0.096	0.28	0.24	0.34	1.54
*CDH11*	PE	0.14	0.34	0.51	0.89	1.23
	GDM	**0**.**026**	0.12	0.069	0.096	1.55
	SGA	0.26	0.51	0.18	0.18	1.18
	LGA	0.68	0.83	0.99	0.99	1.07

a Mid-gestation marker genes with statistically non-significant results are given in **Table S8**.

b Cases included maternal pregnancy complications (preeclampsia, PE, *n* = 12; gestational diabetes mellitus, GDM, *n* = 12), fetal pregnancy complications (small-for-gestational-age, SGA, *n* = 12; large-for-gestational-age, LGA, *n* = 12), as well as control samples comprised of uncomplicated pregnancies resulting in the birth of an appropriate-for-gestational-age newborn (AGA, *n* = 12).

c *P*-values from RT-qPCR data were estimated with Student t-test (no adjustment). For seven genes with significant t-test *P*-values, analysis of covariance (ANCOVA) adjusted by estimated confounder effects was used. All tests were adjusted by gestation age, placenta weight, infant gender and type of delivery. In case of GDM, tests were additionally adjusted by infant weight and maternal age, and with infant weight in case of PE. Bold letters highlight the genes with statistically significant values throughout analyses. T-test FDR correction considered 16 genes. ANCOVA FDR considered 7 genes.

d Fold change was calculated as the difference of mean relative expression values of each pregnancy complication vs control group of uncomplicated pregnancies.

Four further genes (*GATM*, *GPR183*, *MEG3*, *CDH11*) were identified with a considerable trend for aberrantly increased expression in placentas from complicated pregnancies, although statistical tests for group comparisons did not remain significant after correction for multiple testing (FDR *P*>0.05). Transcript level of *GATM* was higher in all studied pregnancy complications compared to normal term placentas (t-test; PE: *P* = 0.025, fc = 1.41; GDM: *P* = 0.024, fc = 2.08; SGA: *P* = 0.022, fc = 1.49; LGA: *P* = 0.019, fc = 1.44; **Table 3**). *GATM* encodes a mitochondrial enzyme involved in creatine biosynthesis (**Table 2**). *GPR183* contributing to humoral immunity showed an increased placental expression in affected fetal growth (SGA, *P* = 0.025, fc = 1.52; LGA, *P* = 0.029, fc = 1.41). The expression of the imprinted non-coding RNA encoding *MEG3* and cell-cell adhesion mediating *CDH11* were increased in GDM cases (*P* = 0.012, fc = 2.13 and *P* = 0.026, fc = 1.55, respectively).

Other tested mid-gestation genes (*BMP5, FST, ITGBL1, NEDD9, NR3C1, NRCAM, PLAGL1, SLC16A10, ZFP36L1*) showed no clear trend for the difference in expression levels between complicated pregnancies and the control group (**Table S8**).

The three genes (*STC1*, *CCNG2*, *LYPD6*) with the strongest association to increased transcription in term placentae of complicated pregnancies were selected for validation of protein expression, either in maternal plasma by ELISA (STC1) or in placenta tissue samples by immunohistochemistry (IHC) (CCNG2, LYPD6).

### Significantly Elevated Maternal Plasma STC1 Level in Pregnancy Complications

**Figure 5:**
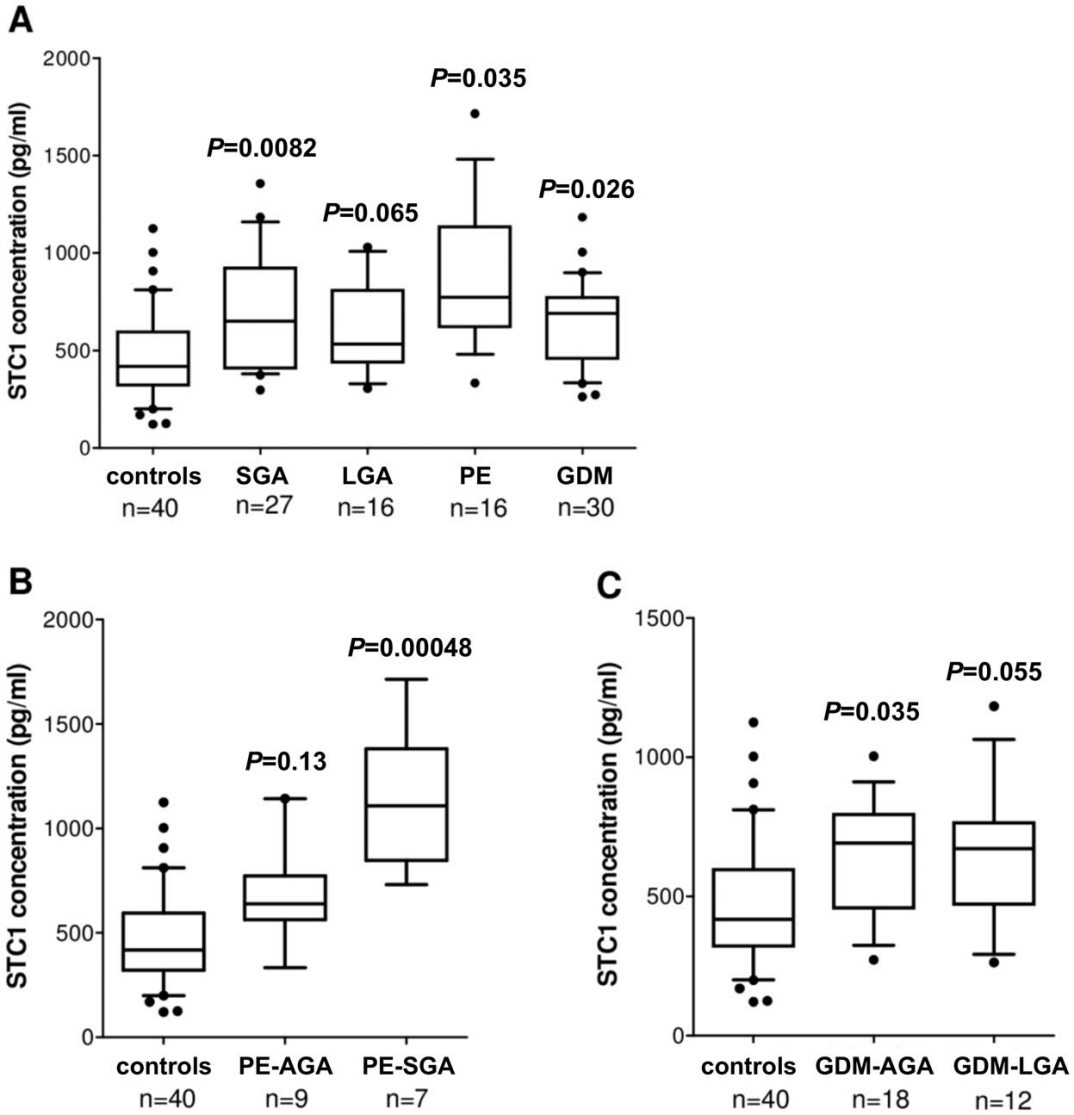
Glycoprotein hormone STC1 protein levels in maternal blood plasma. STC1 protein levels in maternal plasma from uncomplicated pregnancies (defined as controls) compared to (A) pregnancies resulting in the birth of small-for-gestational-age (SGA) and large-for-gestational-age (LGA) newborns, as well as pregnancies complicated with preeclampsa (PE) or gestational diabetes mellitus (GDM); (B) cases with PE and (C) GDM grouped by newborn birth weight. Median values are indicated by horizontal bars. Plotted values are represented with no adjustment for confounding effects. Statistical differences between controls and each of the patient groups were assessed by accounting for confounding factors with ANCOVA. Statistical tests were adjusted for newborn birth-weight (initial analyses of PE and GDM cases), gestational age (SGA, LGA, PE), mode of delivery and mother’s weight (all groups), height (PE, except when grouped by newborns birth weight) and age (GDM). The adjusted *P*-values are given above the data point of the respective study group.

Circulating STC1 is induced during gestation and lactation in mice, indicating its potential role as a regulator of metabolism in pregnancy [25]. In Human Protein Atlas (http://www.proteinatlas.org/), the placental expression of human STC1 is shown in cytoplasm and membranes of trophoblasts as well as in decidual cells [26]. Here we asked whether the increase in placental *STC1* mRNA in pregnancy complications correlates with the higher maternal circulating STC1 levels. The concentration of soluble secreted glycoprotein hormone STC1 [27] was measured by ELISA in post-partum maternal plasma samples of PE and GDM patients or mothers of newborns with affected intrauterine growth (SGA, LGA) (**Table 1B** **)**. Median concentration of STC1 protein in plasma in the GDM (median 690 pg/ml, range 263–1183; ANCOVA, *P* = 0.026) and the PE group (median 773 pg/ml, range 332–1715; ANCOVA, *P* = 0.035) was significantly elevated compared to control samples (median 418 pg/ml, range 124–1125) (**Figure 5A**). Significantly increased level of plasma STC1 was also detected in uncomplicated pregnancies resulting in the birth of a SGA, in comparison to controls (median 650 pg/ml, range 373–1355; ANCOVA, *P* = 0.0082). Mothers in the LGA group showed a nearly significant trend for the increased STC1 levels (median 532 pg/ml, range 305–1029; *P* = 0.065) (**Figure 5A**).

Preeclampsia and gestational diabetes mellitus may occur solely as a maternal disease or in combination with affected fetal growth (PE-SGA, GDM-LGA) [28], [29]. When PE cases were grouped based on the birth-weight of newborns, increase in maternal plasma concentrations of STC1 was strongly more pronounced in the PE-SGA group (median 731 pg/ml, range 1108–1715; ANCOVA, *P* = 0.00048) (**Figure 5B**). In contrast, when GDM patients were further classified based on the delivery of large (GDM-LGA) or normal-weight baby, the statistical significance of the higher maternal STC1 was enhanced in neither of the subgroups compared to the full GDM group analysis. (**Figure 5C**).

### Expression and Localization of LYPD6 and CCNG2 Proteins in Term Placental Tissue from Normal and Complicated Pregnancies

To determine protein expression levels of cytoplasmic molecules of LYPD6 [24] and CCNG2 [30], immunohistochemical (IHC) staining was performed in placental sections from control (*n* = 5), GDM (*n* = 5) and PE (*n* = 5) pregnancies. Characteristics of the IHC study groups are provided in **Table S1**.

**Figure 6:**
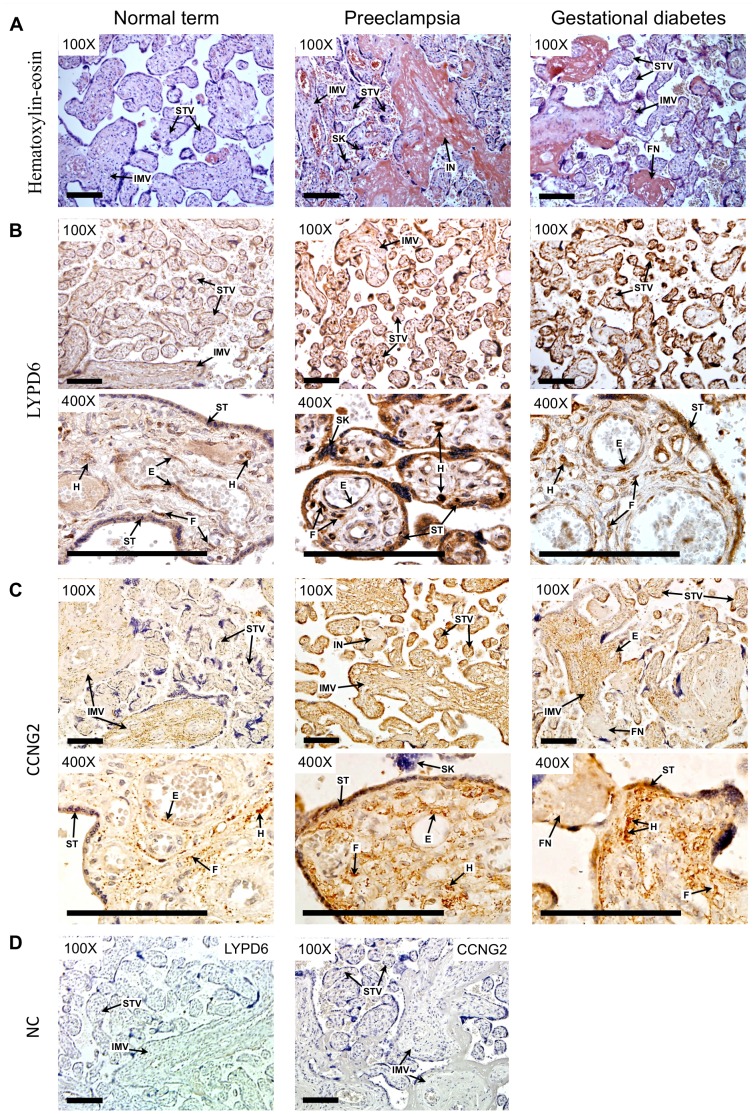
The immunostaining of CCNG2 and LYPD6 proteins was assessed in placental sections from term pregnancies with no complications (controls), with preeclampsia (PE) or with gestational diabetes mellitus (GDM). (A) Hematoxylin-eosin staining was used to describe histopathological findings in analyzed placental samples (100-fold microscope magnification). In term placentae the mature intermediate (IMV) and small terminal villi (STV) were seen. Characteristic to PE, villous agglutination and infarction (IN; intense eosinophilic staining) and increased number of syncytial knots (SK) were detected. GDM presented with degenerative placental lesions such as focal villous fibrinoid necrosis (FN). (B) Diffuse cytoplasmic staining of LYPD6 antibody was detected in syncytiotrophoblast (ST) cells in all villous types (IMV, STV). Additionally LYPD6 antibody strongly stained the cytoplasm and the nucleus of villous stroma Hoffbauer cells (H), fibroblasts (F) and endothelial cells (E) of villous vessels. No localization differences in LYPD6 antibody stain between the groups were found; however strong tendency to higher staining intensity was observed in PE and GDM placentas compared to normal term placenta. (C) CCNG2 antibody showed fine granular cytoplasmic staining of villous stromal Hoffbauer (H) and fibroblast (F) cells. In addition, weak cytoplasmic staining of syncytiotrophoblast (ST) and endothelial cells (E) of vessel wall was found. No localization differences in CCNG2 staining between the normal, PE and GDM groups were detected. Higher tendency to positivity was seen in PE placental sections. (D) Negative control (NC) staining was performed without primary antibody. Scale bar, 100 µm. Microscope magnifications ×100 and ×400 were used. Brown color indicates chromogen-labeled antibody and blue color indicates hematoxylin nuclear staining.

LYPD6 antibody showed a strong cytoplasmic and nuclear staining of placental villous Hoffbauer cells, fibroblasts and endothelial cells of villous vessels. Most importantly, all types of chorionic villi exhibited a diffuse cytoplasmic LYPD6 staining of syncytiotrophoblasts. Although the placental cellular localization of LYPD6 did not differ among the study groups, the staining intensity of the LYPD6 antibody was notably stronger in placental sections from all studied five PE and five GDM cases (**Figure 6B**; **Figure S5**). This observation is consistent with RT-qPCR results showing significant increase in *LYPD6* transcript levels in GDM and PE (**Table 3**).

The CCNG2 antibody staining revealed a fine granular cytoplasmic expression of CCNG2 in villous stromal Hoffbauer cells and fibroblast cells. Intermediate villi enriched in stromal cells exhibited stronger staining compared to small terminal villi. In addition, weak cytoplasmic staining of syncytiotrophoblast and endothelial cells of blood vessel walls was detected. The cellular localization of CCNG2 in placenta was similar in all study groups. The staining intensity, however, was stronger in all PE and GDM placental sections compared to control samples, consistent with the differential expression at the mRNA level (**Figure 6C**, **Table 3**, **Figure S6**).

## Discussion

Placenta is a temporary organ with rapid changes in structure and function across relatively short lifespan to support the establishment and the normal progression of pregnancy until delivery. However, information on the temporal dynamics of placental gene expression is scarce. Only three published studies have focused on differential expression at term compared to the first or the second trimester placenta [10]–[12], and there is only one published report addressing differential expression between the first and second trimester placentas with no attempt to provide experimental validation of microarray experiments [12].

This is the first study that reports quantitative profiling of placental transcriptome dynamics across three months from early to mid-pregnancy (10 samples; gestational weeks 5 to 18). The main strengths of the present analysis compared to the previous report [12] involve the experimental validation of selected top genes from microarray analysis, and further replication of mid-gestation expression in an extended sample-set representing first, second and term placentae (*n* = 43; gestational weeks 5 to 41). However, a number of limitations restrict the interpretation of our results. The current study covers only a relatively small number of mid-gestation placental samples (*n* = 8) as the acquisition of such rare human samples involves major clinical and ethical restrictions. In addition, trophoblast samples collected after early termination of pregnancy may include contamination of maternal cells. Unfortunately no generally agreed placental reference genes are currently available with known stable expression profile over all three trimesters of pregnancy. In Taqman RT-qPCR conformation experiments, we used *HPRT1* as a reference gene, assuming its constant expression in pregnancy. In fact, *HPRT1* has been shown to maintain a stable expression level during early pregnancy [31] and in term placenta when normal, PE and GDM samples were compared [32]. We have successfully applied *HPRT1* as a reference gene to study of the expression of selected placental genes in the first, second and third trimester [33].

Two alternative statistical approaches for gene expression microchip analyses detected in total 154 genes (ANOVA, FDR *P*<0.1; **Figure 1** **,** **Table S2**) and 229 genes (empirical Bayes moderated t-test, FDR-corrected *P*<0.1; **Figure S3**; **Table S4**) with significant change in gene expression from gestational week 5 to 18. The two analyses consistently demonstrated that the majority of genes showed increasing transcript levels. Ten of the 24 loci (**Figure 2** **, Table S3**) selected for confirmation experiments in an extended sample set showed highly significant expressional peak in mid-gestation placenta with a drop in transcript levels at term (**Figure 3** **;** **Figure 4**). Among these, *CDH11* and *STC1* [11], [12], *CCNG2* [11], *SLC16A10* and *FST* [12] have been reported previously to show significant reduction in placental expression at term compared to mid-pregnancy. Notably, several of the identified genes with specific expression in mid-gestation placenta have been reported to be involved in regulation of mammalian placental function and/or embryonic development (**Table 2** **,** extended references in **Table S9**). *STC1/Stc1* encoding stanniocalcin 1 contributes to implantation process in human [34], pigs [35] and sheep [36], and cyclin-G2 (gene *Ccng2*) in mice [37]. Cadherin 11 coded by *CDH11* functions in trophoblast cell differentiation [38] and a lack of *Zfp36L1* expression in murine midgestation results in abnormal placentation and fetal death [39]. Bone morphogenetic protein 5 (gene: *BMP5)* [40], cadherin 11 (*CDH11*) [41], follistatin (*FST*) [42], [43] and stanniocalcin 1 (*STC1*) [23] contribute to bone formation, mineralization and skeletal growth, as well as muscle development and growth. *STC1* and *GATM* (encoding glycine amidino transferase) are involved in kidney and nervous system development [23], [44], and paternally expressed *PLAGL1*/*Zac1* in cardiac morphogenesis and the development of pancreas [20], [45]–[47]. The correct imprinting of maternally expressed imprinted *MEG3* plays a general crucial role in normal embryonic development and growth [48], [49].

An additional innovation of our study is the testing of a novel hypothesis that late pregnancy complications may be accompanied by abnormal placental expression of mid-gestation genes in late pregnancy, such as insufficient down-regulation of genes that are normally inhibited at term, or further up-regulation of genes that normally reach a plateau of constant expression already in mid-pregnancy. Our study strongly supports the idea that profound changes are required in gene expression profile in normal term compared to early- and mid-pregnancy placenta. The present study investigated our postulated hypothesis in the context of maternal pregnancy complications of preeclampsia (PE) and gestational diabetes mellitus (GDM), as well as affected fetal growth (LGA, SGA). The gene *STC1* coding for stanniocalcin 1 (STC1) was identified with a sharp placental expressional peak in mid-gestation (**Figure 3** **;** **Figure 4**) and increased mRNA levels at term in all pregnancy complications compared to controls, with the most significant effect in PE and SGA groups (**Table 3**). Increased levels of placental *STC1* in affected fetal growth detected in the current study are consistent with the reduced fetal growth of transgenic mice over-expressing human *STC1* [50]. In agreement with placental mRNA data, STC1 protein levels were elevated in post-partum maternal plasma in PE, SGA and GDM pregnancy complications (**Figure 5**). The glycoprotein hormone STC1 functions mainly in an autocrine/paracrine manner in many organs and is implicated in regulation of calcium and phosphate homeostasis [23]. Induction of circulating STC1 in murine pregnancy has indicated its role in gestational metabolism [25]. Further underlining its importance in renal homeostasis [51], [52], we report significant increase in placental *STC1* expression and in maternal post-partum plasma STC1 in preeclampsia, known to be accompanied by impaired renal function. It remains to be studied whether elevated circulating STC1 in maternal plasma partially originates from placenta or reflects maternal internal response to PE pathophysiology. Although we conducted a small-scale study based on biological material collected post-partum, the observed significantly elevated maternal plasma levels of STC1 in pregnancy complications warrant further investigations of its potential as a prognostic biomarker of the pregnancy course. The only published study on human circulating STC1 level has reported significantly increased serum STC1 in ovarian cancer patients [53].

It is noteworthy that the highest plasma levels of STC1 were measured for the cases, who had developed simultaneously maternal PE and given birth to an SGA baby (median 731 pg/ml *vs* 418 pg/ml in controls; ANCOVA, *P* = 0.00048). This data refers to partially common pathologic origin for those conditions [6], [28]. A possible link to the up-regulation of *STC1* in both disturbed maternal and fetal metabolism may be stress conditions, such as low oxygen concentrations. *STC1* promoter harbors a binding site for *HIF-1* transcription factor regulating the genes involved in cellular responses to hypoxia [54]. An earlier study demonstrated the HIF-1 protein mediated activation of *STC1* expression in hypoxic human cancer cells [55].

In addition to *STC1*, PE as well as GDM placentae were characterized by significantly higher gene expression of *LYPD6* (encoding LY6/PLAUR domain containing 6) and *CCNG2* (Cyclin-G2) (**Table 3**), confirmed by the stronger staining intensity of LYPD6 and CCNG2 antibodies on PE and GDM placental sections in performed IHC experiments (**Figure 6** **, Figure S5, Figure S6**). LYPD6 was identified as a novel protein expressed in syncytiotrophoblast. The only published report on *LYPD6* showed that its over-expression suppresses activator protein 1 (AP-1) -mediated transcriptional activity [24]. In normal human placenta, AP-1 transcription factors are specifically expressed in intermediate extravillous trophoblasts to regulate their invasiveness [56], as well as proliferation and differentiation of cytotrophoblasts [57]. Cyclin G2 inhibits cell cycle in response to diverse growth inhibitory signals, such as heat shock, oxidative stress, hypoxia, DNA damage and differentiation [30], [58]. Increased expression of CCNG2 in placentas of pregnancy complications may reflect local cellular stress conditions.

A recent study on mice revealed a new, direct role of placental metabolic pathways in regulating fetal brain development by placenta-derived serotonin and provided experimental evidence for the role of maternal-placental-fetal interactions in developmental programming of mental health [59]. Interestingly, the majority of highly expressed genes in mid-gestation placenta identified in the current study have been also linked to human complex disease (**Table 2** **;** references in **Table S9**). These observations promote speculations on a possible wider effect of placental gene expression on the biological bases of developmental origins of human disease [60]. Among the genes with the peak expression in the mid-pregnancy followed by a drop at term, *BMP5* [61], *CDH11* [62] and *FST* [63] are implicated in bone and cartilage related disorders such as rheumatoid arthritis and osteoarthritis. *GATM* and *STC1* have been among the top loci in genome-wide association studies of chronic kidney disease [51], [52], and have also been implicated in heart failure [64], [65]. *MEG3* [66] and *GPR183* [67] were shown to be involved in the development of type 1 diabetes. Among the few genes which continued steady rise in placental expression until term, *NR3C1* coding for glucocorticoid receptor has been implicated in rheumatism [68] and the malfunction of *NRCAM* and *NEDD9* is related to brain disorders such as autism [69], Alzheimer’s and Parkinson’s disease [70], [71]. Interestingly, *LYPD6* is also located within a microduplication region linked to developmental delay [72]. In addition, the majority of midgestation-related genes are involved in various types of cancer (**Table 2** **,** references in **Table S9**).

### Conclusions

The study identified a pool of transcripts with a sharp expressional peak in mid-gestation pregnancy and demonstrated the importance of fine-scale tuning of the temporal dynamics of placental transcriptional regulation relevant to each gestational period. Interestingly, the majority of genes with high expression in mid-gestation placenta have also been implicated in adult complex disease, promoting the discussion on the role of placenta in developmental programming [73]. The discovery of elevated maternal plasma STC1 in pregnancy complications warrants further investigations of its potential as a biomarker.

## Supporting Information

Text S1 **Group-based microarray analysis comparing gene expression differences between early and mid-gestation discovery samples.**
(DOCX)

Text S2 **Experimental details of ELISA measurements of STC1 in maternal blood plasma.**
(DOCX)

Text S3 **Immunohistochemical characterisation of LYPD6 and CCNG2 expression in human placenta.**
(DOCX)

Figure S1 **QC analysis of GeneChip gene expression microarray data by exploring hybridization signal distribution.** Signal (log) intensities in arrays representing the analyzed placental samples from mid-pregnancy (*n* = 4) are illustrated by (A) boxplots of PM (perfect mismatch) intensities (median value was 7), and (B) plots of kernel density estimates of these intensities. QC included comparison of average intensity, correlation with median intensity of other GeneChips, *GAPDH* 3′–>5′ and *β-actin* 3′–>5′, scaling factor, percentage of presence calls, average background and intensities of positive and negative border elements.
(TIF)

Figure S2 **Alternative splice forms of top genes with significant expressional change from early to mid-gestation placenta as identified by Affymetrix HU133 Plus 2 microarray probesets.** Isoforms of 24 genes selected for Taqman RT-qPCR experiments were matched to alternatively spliced transcripts according to the Ensemble database version 68. Orange indicates transcripts matched by a given probeset; asterisk indicates probesets with a significant (FDR *P*<0.1 of ANOVA) change in expression from gestational week 5 to 18 (gestational days 38 to 132).
(TIF)

Figure S3 **Volcano plot for the ∼47,000 transcripts from the group-based comparison of early (** ***n*** **= 6) and mid-gestation (** ***n*** **= 4) samples.** The X-axis shows the log2 fold change (FC), while Y-axis represents FDR corrected *P*-value in –log_10_ scale, computed using empirical Bayes moderated t-test. Out of 24 genes selected for RT-qPCR validation based on the ANOVA analysis of microarray data, 18 genes (showed in volcano plot) were also significant in group-based analysis (FDR *P*<0.1).
(TIF)

Figure S4 **Genes with insignificant expressional change during early and mid-gestation as quantified by RT-qPCR.** Relative mRNA expression levels in extended sample set of first and second trimester placentas (*n* = 31; from gestational days 38 to 147) were determined by TaqMan assays. *P-*values were calculated by ANOVA and subjected to FDR correction.
(TIF)

Figure S5 **Immunostaining of LYPD6 protein was assessed in term placental sections from uncomplicated control, preeclampsia (PE) and gestational diabetes mellitus (GDM) pregnancies.** Diffuse cytoplasmic stain of LYPD6 antibody was detected in syncytiotrophoblast (ST) cells in intermediate (IMV) and small terminal (STV) villi. Additionally LYPD6 antibody strongly stains cytoplasma and nucleus of villous stroma Hoffbauer cells (H), fibroblasts (F) and endothelial cells (E) of villous vessels. Brown staining indicates to chromogen-labeled antibody and blue for hematoxylin nuclear stain. No difference in the localization of LYPD6 antibody stain between the groups was identified, but strong tendency to higher staining intensity in PE and GDM placentas compared to normal term placenta was observed. Scale bar, 50 µm. Microscope magnifications X100 and X400 were used. IN, infarction lesion; SK, syncytial knot.
(TIF)

Figure S6 **The immunostaining of CCNG2 protein was assessed in term placental sections from uncomplicated control, preeclampsia (PE) and gestational diabetes mellitus (GDM) pregnancies.** CCNG2 antibody has fine granular cytoplasmic staining of villous stromal Hoffbauer (H) and fibroblast (F) cells. Weak staining was detected in cytoplasm of syncytiotrophoblast (ST) and endothelial cells (E) of vessel wall in intermediate (IMV) and small terminal (STV) villi. No difference in the localization of CCNG2 antibody stain between the groups was identified, but strong tendency to higher staining intensity in PE and GDM placentas compared to normal term placenta was observed. Scale bar, 50 µm. Microscope magnifications X100 and X400 were used. IN, infarction lesion; FN, fibrinoid necrosis.
(TIF)

Table S1 **Maternal and offspring characteristics of REPROMETA samples used in the study for immunohistochemistry experiments.**
(DOCX)

Table S2 **Differentially expressed placental genes on Affymetrix® GeneChip.** 154 genes (180 probe sets) detected on Affymetrix® GeneChip exhibiting significant (ANOVA, FDR-corrected *P*-value<0.05) or suggestive (*P*-value<0.1) increased or decreased placental expression in the progress of pregnancy from 5^th^ to 18^th^ of gestational week. Fold change in gene expression level as estimated between weeks 5 and 18 of gestational age. For the genes selected for further experiments TaqMan probe sets used in RT-qPCR experiments are provided.
(DOCX)

Table S3 **Biological processes obtained from Gene Ontology Annotation Database (** www.ebi.ac.uk/GOA/ **) for genes exhibiting significantly increased or decreased placental expression in the progress of pregnancy from 5^th^ to 18^th^ of gestational week (ANOVA, FDR** ***P*** **<0.1) and selected for RT-qPCR confirmation.**
(DOCX)

Table S4 **Differentially expressed placental genes on Affymetrix® GeneChip detected by group-based microchip analysis comparing the first (** ***n*** **= 6; gestational days 38, 55, 2×56, 81, 91) and the second (** ***n*** **= 4; gestational days 120, 121, 126, 132) trimester discovery samples.**
(DOCX)

Table S5 **Functional enrichment analysis of up-regulated and down-regulated placental genes identified using group-based analysis (empirical Bayes moderated t-test) comparing early and mid-gestation discovery samples.** Gene Ontology terms and pathways with statistically significant over-representation (FDR p<0.05, hypergrometric test) were retrieved from g:Profiler, separately for genes with induced and inhibited expression patterns. BP, biological process; CC, cellular component; MF, molecular function; ke, KEGG pathway; re, Reactome pathway.
(XLS)

Table S6 **Functional enrichment analysis of up-regulated and down-regulated placental genes identified using dynamic gestation-age dependent statistical analysis (ANOVA).** Gene Ontology terms and pathways with statistically significant over-representation (FDR p<0.05, hypergrometric test) were retrieved from g:Profiler, separately for genes with induced and inhibited expression patterns. BP, biological process; CC, cellular component; MF, molecular function; ke, KEGG pathway; re, Reactome pathway.
(XLS)

Table S7 **TaqMan RTqPCR analysis of genes selected from placenta transcriptome micorarray anaysis.**
(DOCX)

Table S8 **The identified mid-gestation marker genes showing no statistically significant difference in gene expression between the term placental samples from normal pregnancies and the pregnancy complications in the REPROMETA sample collection.**
(DOCX)

Table S9 **Involvement of 16 detected mid-gestation marker genes in adult complex traits and diseases.**
(DOCX)
