# Supplementary material for: Mid-Gestational Gene Expression Profile in Placenta and Link to Pregnancy Complications
Source: PLoS One. 2012 Nov 7;7(11):e49248. doi: 10.1371/journal.pone.0049248 (PMC3492272; doi:10.1371/journal.pone.0049248)
Supplement: Text S1 — Group-based microarray analysis comparing gene expression differences between early and mid-gestation discovery samples. (DOCX) [file pone.0049248.s001.docx]

**Text S1. Group-based microarray analysis comparing gene expression differences between early and mid-gestation discovery samples.**

The alternative analysis of microarray data was based on comparison of gene expression profile differences between two groups of placental samples: early (*n*=6; gestational days 28, 55, 2x56, 81, 91) and mid-gestation (*n*=4; gestational days 120, 121, 126, 132). Differential expression between groups was calculated using empirical Bayes moderated t-statistics using the Limma package of R Bioconductor. Altogether 268 placental transcripts showed significant differential expression in comparison of first and second trimester plancental samples (FDR *P*<0.1; **Figure S3**; **Table S4**), including 205 genes with increased and 24 genes with decreased expression levels.

Functional enrichment analysis with g:Profiler software revealed more than 500 Gene Ontology (GO) categories and pathways that were statistically over-represented in the list of genes with significantly higher transcript levels in the second compared to the first trimester placentae (**Table S5**). The biological process (BP) categories included several development-related groups such as ‘multicellular organismal development’ (*n*=34 genes, FDR *P*=6.3x10^-6^, hypergeometric test), ‘angiogenesis’ (*n*=11 genes, FDR *P*=7.5x10^-6^), ‘cardiovascular system development’ (*n*=13 genes, FDR *P*=8.7x10^-5^), ‘circulatory system development’ (*n*=13 genes, FDR *P*=8.7x10^-5^), ‘anatomical structure development’ (*n*=32 genes, FDR *P*=1.8x10^-4^), ‘cell migration’ (*n*=17 genes, FDR *P*=1.2x10^-5^), ‘cell proliferation’ (*n*=20 genes, FDR *P*=2.6x10^-4^), ‘cell communication’ (*n*=63 genes, FDR *P*=1.6x10^-11^), ‘signal transduction’ (*n*=61 genes, FDR *P*=2.2x10^-12^), immune system process (*n*=28 genes, FDR *P*=9.4x10^-7^) and ‘epigenetic regulation of gene expression’ (*n*=7 genes, FDR *P*=1.2x10^-4^) (**Table S5**). Only 45 enriched GO categories were detected in the list of genes with decreasing expression.

The list of differentially expressed genes identified comparing gene expression between the early and the mid- pregnancy groups (empirical Bayes moderated t-statistics; **Table S4**) overlapped considerably with the list of genes identified in the quantitative microarray analysis of gene expression dynamics across the studied gestational age (gestational days 38 to 132; ANOVA**, Table S2**). For instance, 60% of genes with significantly increased expression levels from the dynamic gestation-age dependent analysis (ANOVA, FDR *P*=0.1, **Table S2**) were also represented as significant in the group-based analysis of first and second trimester samples (**Table S4**). Importantly, the common list of genes included 17 genes selected for further RT-qPCR validation (**Figure S3**). In addition, enriched GO categories for gene lists detected from both analyses reflected similar or identical biological processes characteristic to placental and/or fetal development (**Table S5**; **Table S6**).
