# Supplementary material for: Mid-Gestational Gene Expression Profile in Placenta and Link to Pregnancy Complications
Source: PLoS One. 2012 Nov 7;7(11):e49248. doi: 10.1371/journal.pone.0049248 (PMC3492272; doi:10.1371/journal.pone.0049248)
Supplement: Text S2 — Experimental details of ELISA measurements of STC1 in maternal blood plasma. (DOCX) [file pone.0049248.s002.docx]

**Text S2. Experimental details of ELISA measurements of STC1 in maternal blood plasma**

ELISA assays were conducted according to the manufacturer’s instructions and all the measurements were performed at least in duplicate.

In brief, high-binding, flat-bottom polypropylene microplates (Costar^®^, R&D Systems) were coated overnight at room temperature with 100 μL of goat anti-human STC1 antibody (1.6 μg/mL). The plate was washed three times with PBS containing 0.05% Tween-20 (PBST) and blocked with Reagent Diluent (PBS containing 1% bovine serum albumin, R&D Systems) for 1 hr. Either 100 μL of a sample or 100 μL of a diluted STC1 standard (47.3–3030 pg/mL; seven dilutions) were added to antigen-coated wells in duplicate. After 2 hours of incubation at room temperature and three washes with PBST, the plate was treated with a second biotinylated goat anti-human STC1 detection antibody (400 ng/mL in Reagen Diluent containing 2% heat inactivated normal goat serum) for 2 hours and after washes followed by a 1:175 dilution of Streptavidin-HRP conjugate for 20 min. Tetramethylbenzidine substrate (Sigma-Aldrich, Inc, Saint Louis, MO) was added for 20 min and the reaction was stopped by addition of equal volume of Stop Reagent (Sigma-Aldrich, Inc). The absorbance at 450 nm was determined for each well by using Tecan Sunrise Basic microplate reader with Magellan™ software (Tecan Austria GmbH).
