# Supplementary material for: Mid-Gestational Gene Expression Profile in Placenta and Link to Pregnancy Complications
Source: PLoS One. 2012 Nov 7;7(11):e49248. doi: 10.1371/journal.pone.0049248 (PMC3492272; doi:10.1371/journal.pone.0049248)
Supplement: Text S3 — Immunohistochemical characterisation of LYPD6 and CCNG2 expression in human placenta. (DOCX) [file pone.0049248.s003.docx]

**Text S3. Immunohistochemical characterisation of LYPD6 and CCNG2 expression in human placenta**

The removed specimens were immediately fixed in the buffered 10% formaline (pH 7.4) for 24 hours and subsequently embedded into paraffin wax as routinely performed. From the resulting tissue blocks, serial paraffin sections 6 μm were cut and placed on glass slides for hematoxylin-eosin (H&E) stain and immunohistochemistry (IHC). The standard H&E staining was used for the evaluation of histopathological changes.

To identify and localize the LYPD6 and CCNG2 proteins, five paraffin embedded placental tissue sections from each group (control, PE, GDM, **Table S1**) as well as negative control without the primary antibody were used for each assay. For immunostaining, the sections were deparaffinized and subsequently incubated for antibody retrieval in citrate buffer (pH 6.0) for 30 min, treated with hydrogen peroxide for 15 min and blocked with 4% non-fat milk for 30 min at room temperature. Subsequently, the tissue sections were incubated with the specific (i) anti-human LYPD6 antibody (1:30, LS-C102542, LifeSpan BioSciences, Seattle, USA) or (ii) anti-human CCNG2 (Santa Cruz Biotechnology, sc-7266, 1:100) and secondary biotinylated polyclonal rabbit anti-goat (DakoCytomation, Denmark, E 0466, 1:400) antibodies. Primary antibody reactions were performed overnight at 4°C under humid conditions and stainings were performed at room temperature. All solutions and buffers were provided by DAKO (Hamburg, Germany). After several washings, the antigen-antibody complex was visualized by using DAKO REAL^TM^ EnVision Detection System, Peroxidase/DAB+, Rabbit/Mouse. Slides were counterstained with hematoxyline, dehydrated and coverslipped with Permount (Fisher Scientific) for light microscopy. IHC stainings were carried out in duplicate.

Imagining was performed with the Olympus BX60 microscope using Olympus DP71 digital camera and CellA imaging software (Olympus Optical). Microscope magnifications ×100 and ×400 were used. All measurements were acquired at the same light intensity and processed by same way.
