# Supplementary figures and images for: Mid-Gestational Gene Expression Profile in Placenta and Link to Pregnancy Complications
Source: PLoS One. 2012 Nov 7;7(11):e49248. doi: 10.1371/journal.pone.0049248 (PMC3492272; doi:10.1371/journal.pone.0049248)

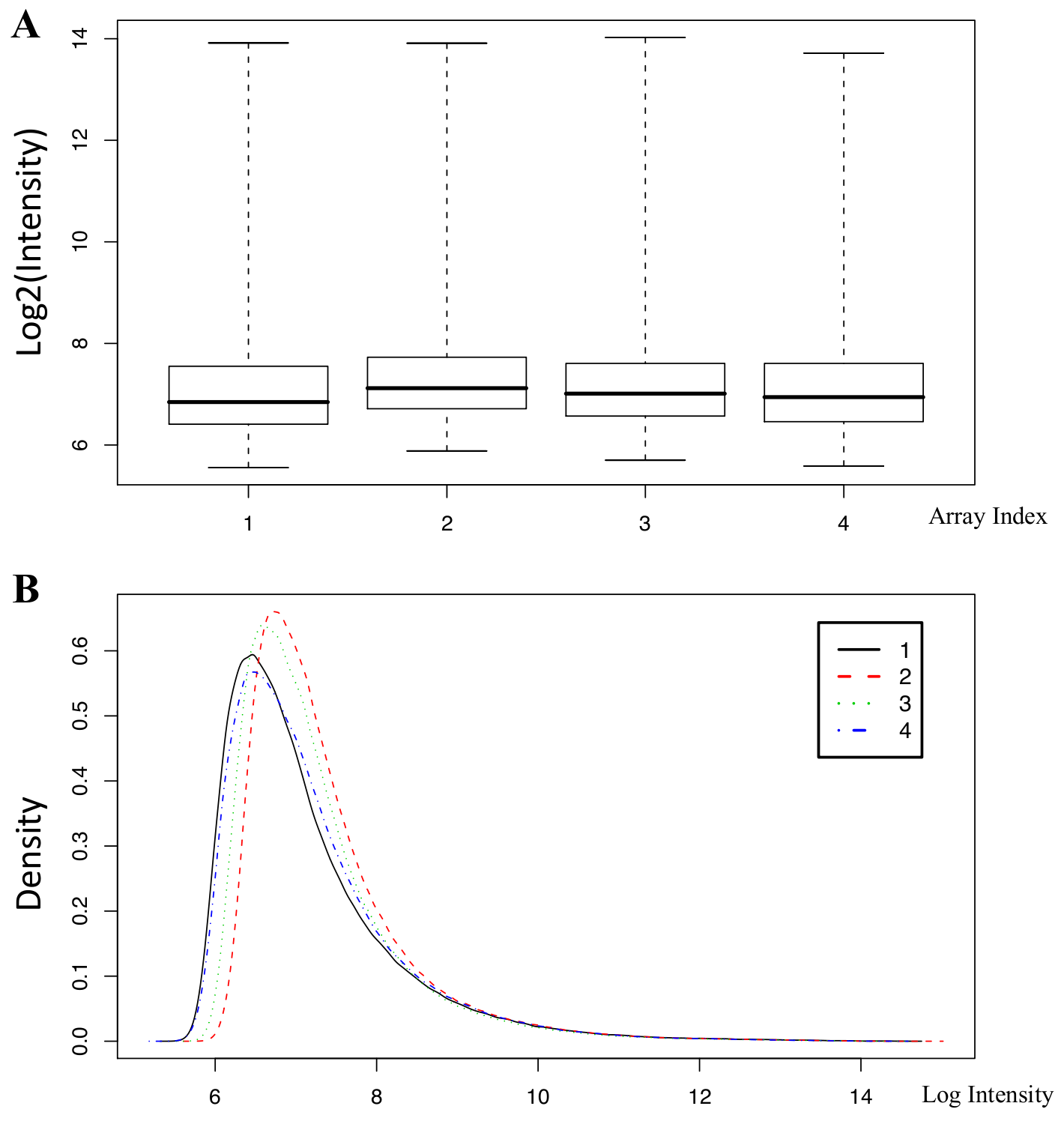

Supplement: Figure S1 — QC analysis of GeneChip gene expression microarray data by exploring hybridization signal distribution. Signal (log) intensities in arrays representing the analyzed placental samples from mid-pregnancy (n = 4) are illustrated by (A) boxplots of PM (perfect mismatch) intensities (median value was 7), and (B) plots of kernel density estimates of these intensities. QC included comparison of average intensity, correlation with median intensity of other GeneChips, GAPDH 3′–>5′ and β-actin 3′–>5′, scaling factor, percentage of presence calls, average background and intensities of positive and negative border elements. (TIF) [file pone.0049248.s004.tif]

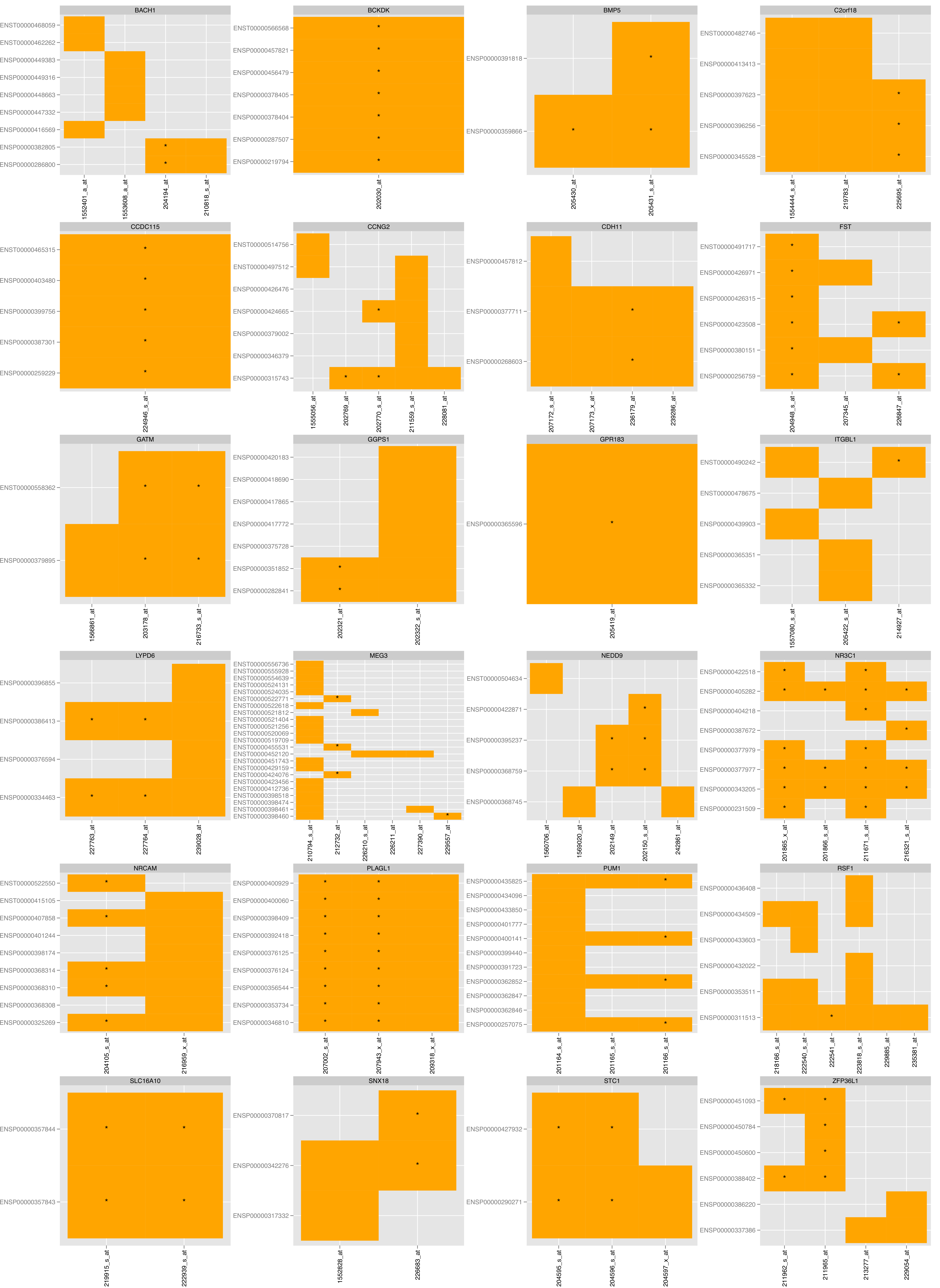

Supplement: Figure S2 — Alternative splice forms of top genes with significant expressional change from early to mid-gestation placenta as identified by Affymetrix HU133 Plus 2 microarray probesets. Isoforms of 24 genes selected for Taqman RT-qPCR experiments were matched to alternatively spliced transcripts according to the Ensemble database version 68. Orange indicates transcripts matched by a given probeset; asterisk indicates probesets with a significant (FDR P<0.1 of ANOVA) change in expression from gestational week 5 to 18 (gestational days 38 to 132). (TIF) [file pone.0049248.s005.tif]

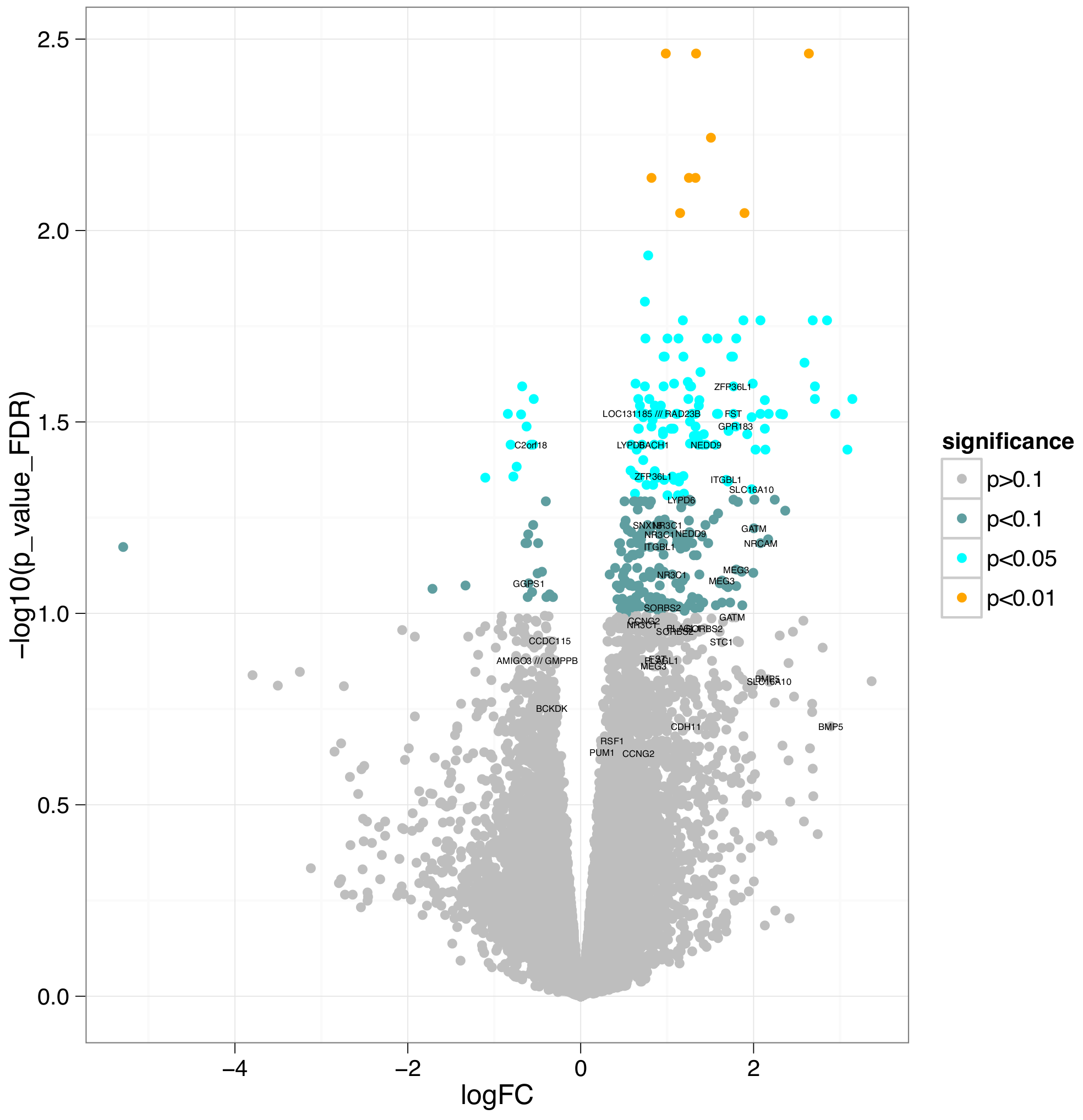

Supplement: Figure S3 — Volcano plot for the ∼47,000 transcripts from the group-based comparison of early ( n = 6) and mid-gestation ( n = 4) samples. The X-axis shows the log2 fold change (FC), while Y-axis represents FDR corrected P-value in –log10 scale, computed using empirical Bayes moderated t-test. Out of 24 genes selected for RT-qPCR validation based on the ANOVA analysis of microarray data, 18 genes (showed in volcano plot) were also significant in group-based analysis (FDR P<0.1). (TIF) [file pone.0049248.s006.tif]

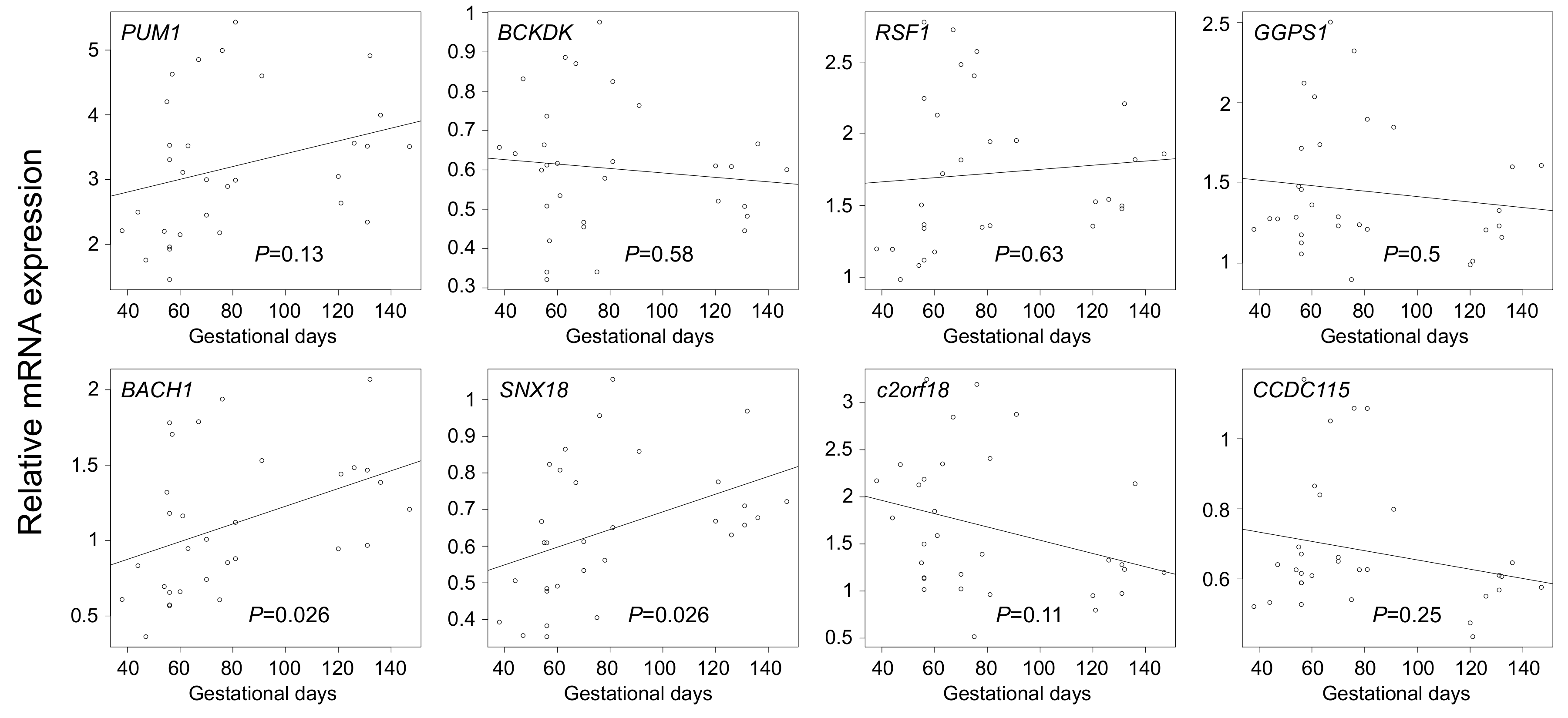

Supplement: Figure S4 — Genes with insignificant expressional change during early and mid-gestation as quantified by RT-qPCR. Relative mRNA expression levels in extended sample set of first and second trimester placentas (n = 31; from gestational days 38 to 147) were determined by TaqMan assays. P-values were calculated by ANOVA and subjected to FDR correction. (TIF) [file pone.0049248.s007.tif]

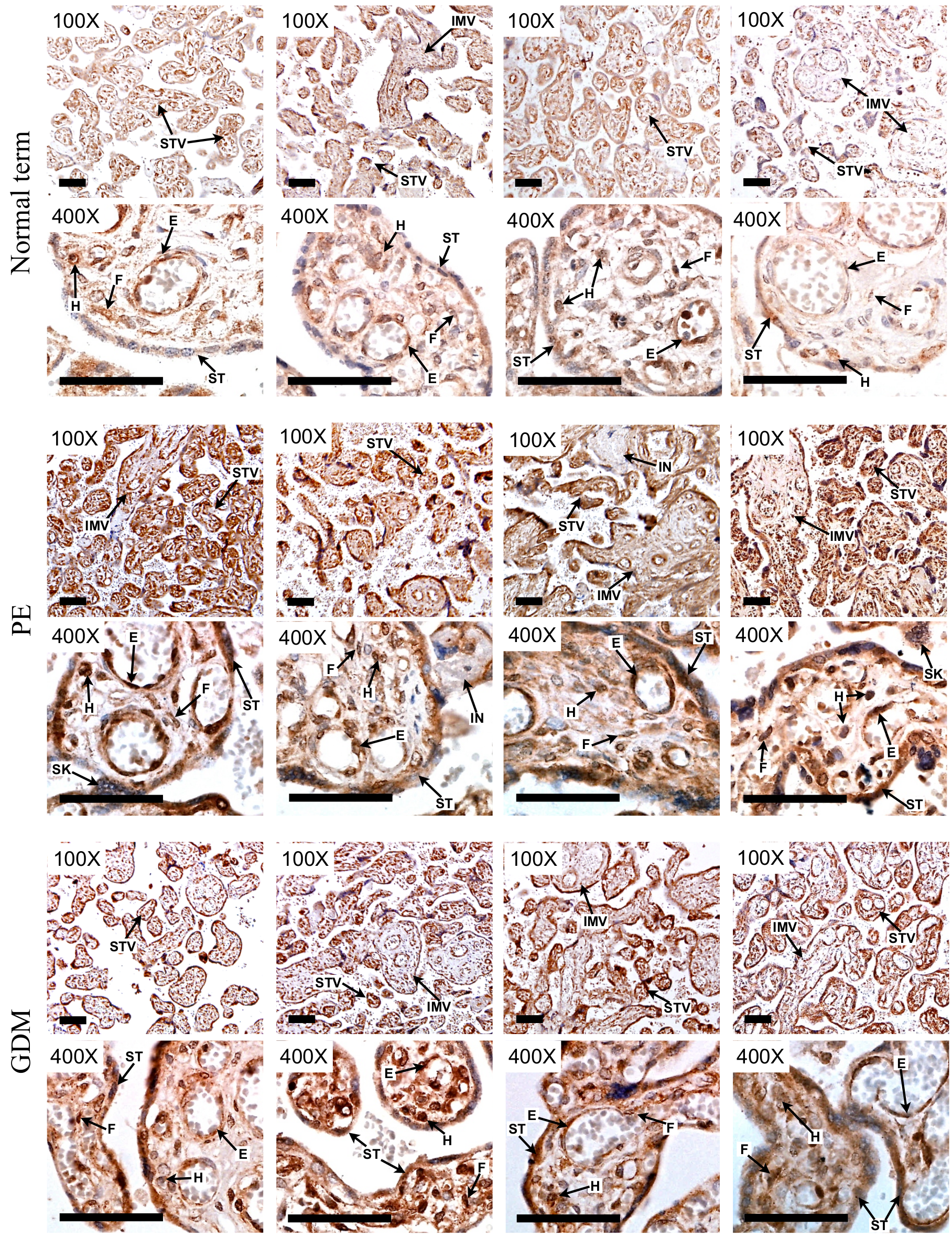

Supplement: Figure S5 — Immunostaining of LYPD6 protein was assessed in term placental sections from uncomplicated control, preeclampsia (PE) and gestational diabetes mellitus (GDM) pregnancies. Diffuse cytoplasmic stain of LYPD6 antibody was detected in syncytiotrophoblast (ST) cells in intermediate (IMV) and small terminal (STV) villi. Additionally LYPD6 antibody strongly stains cytoplasma and nucleus of villous stroma Hoffbauer cells (H), fibroblasts (F) and endothelial cells (E) of villous vessels. Brown staining indicates to chromogen-labeled antibody and blue for hematoxylin nuclear stain. No difference in the localization of LYPD6 antibody stain between the groups was identified, but strong tendency to higher staining intensity in PE and GDM placentas compared to normal term placenta was observed. Scale bar, 50 µm. Microscope magnifications X100 and X400 were used. IN, infarction lesion; SK, syncytial knot. (TIF) [file pone.0049248.s008.tif]

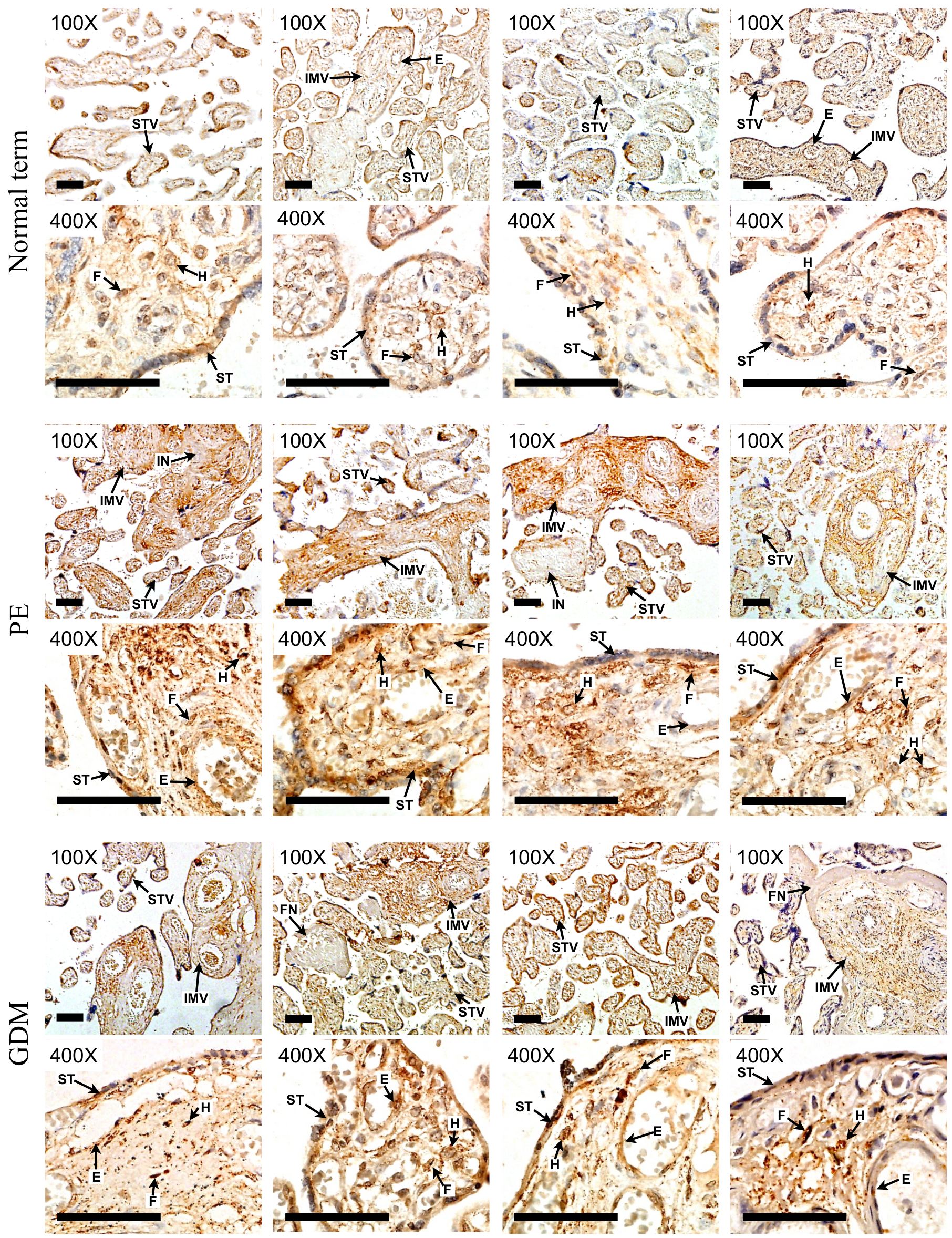

Supplement: Figure S6 — The immunostaining of CCNG2 protein was assessed in term placental sections from uncomplicated control, preeclampsia (PE) and gestational diabetes mellitus (GDM) pregnancies. CCNG2 antibody has fine granular cytoplasmic staining of villous stromal Hoffbauer (H) and fibroblast (F) cells. Weak staining was detected in cytoplasm of syncytiotrophoblast (ST) and endothelial cells (E) of vessel wall in intermediate (IMV) and small terminal (STV) villi. No difference in the localization of CCNG2 antibody stain between the groups was identified, but strong tendency to higher staining intensity in PE and GDM placentas compared to normal term placenta was observed. Scale bar, 50 µm. Microscope magnifications X100 and X400 were used. IN, infarction lesion; FN, fibrinoid necrosis. (TIF) [file pone.0049248.s009.tif]
