# Supplementary material for: Mid-Gestational Gene Expression Profile in Placenta and Link to Pregnancy Complications
Source: PLoS One. 2012 Nov 7;7(11):e49248. doi: 10.1371/journal.pone.0049248 (PMC3492272; doi:10.1371/journal.pone.0049248)
Supplement: Table S1 — Maternal and offspring characteristics of REPROMETA samples used in the study for immunohistochemistry experiments. (DOCX) [file pone.0049248.s010.docx]

**Table S1.** Maternal and offspring characteristics of REPROMETA samples used in the

study for immunohistochemistry experiments.

| Mother and offspring characteristics | Control | PE | GDM |
| --- | --- | --- | --- |
| No of women [nulliparity] | 5 [4] | 5 [5] | 5 [4] |
| Maternal age (yr) | 31 (23; 40) | 24 (23; 27) | 26 (21; 36) |
| Maternal height (cm) | 166 (161; 168) | 170.5 (156; 178) | 157 (150; 162)* |
| Maternal pre-pregnancy weight (kg) | 59 (53; 69) | 68.8 (50; 80) | 68 (46; 92) |
| Gestational weight gain (kg) | 12 (9; 25) | 18 (11.5; 21.5) | 13 (8; 17) |
| Delivery mode (vaginal/c-section) | 4/1 | 1/4 | 1/4 |
| Smokers during pregnancy (n) | 0 | 0 | 0 |
| Gestational age at birth (d) | 268 (259; 272) | 269 (263; 287) | 269 (266; 276) |
| Baby’s birth-weight (g) | 3450 (2890; 3730) | 2896 (2760; 4250) | 4030 (3168; 5420) |
| Baby’s birth length (cm) | 50 (49; 51) | 49 (48; 51)* | 51 (49; 55) |
| Baby’s head circumference (cm) | 35 (33; 36.5) | 35.5 (32.5; 37) | 36.5 (34.5; 38) |
| Baby’s abdominal circumference (cm) | 34 (33; 35.5) | 31.5 (30.5; 37.5) | 37 (34; 40.5) |
| Placental weight | 455 (410; 630) | 500 (440; 770) | 620 (410; 860) |
| No of newborns born SGA/LGA | 0/0 | 0/0 | 0/3 |
| No of boys/girls | 4/1 | 2/3 | 3/2 |

Data are given as medians with ranges, except where indicated differently.

**P<*0.05 *vs*. control group, Mann-Whitney U or Fisher’s Exact test .

PE, preeclampsia; GDM, gestational diabetes mellitus; yr, years; d, day.
