# Supplementary material for: Mid-Gestational Gene Expression Profile in Placenta and Link to Pregnancy Complications
Source: PLoS One. 2012 Nov 7;7(11):e49248. doi: 10.1371/journal.pone.0049248 (PMC3492272; doi:10.1371/journal.pone.0049248)
Supplement: Table S2 — Differentially expressed placental genes on Affymetrix® GeneChip. 154 genes (180 probe sets) detected on Affymetrix® GeneChip exhibiting significant (ANOVA, FDR-corrected P-value<0.05) or suggestive (P-value<0.1) increased or decreased placental expression in the progress of pregnancy from 5th to 18th of gestational week. Fold change in gene expression level as estimated between weeks 5 and 18 of gestational age. For the genes selected for further experiments TaqMan probe sets used in RT-qPCR experiments are provided. (DOCX) [file pone.0049248.s011.docx]

**Table S2**. Differentially expressed placental genes on Affymetrix® GeneChip. 154 genes (180 probe sets) detected on Affymetrix® GeneChip exhibiting significant (ANOVA, FDR-corrected *P*-value<0.05) or suggestive (*P*-value<0.1) increased or decreased placental expression in the progress of pregnancy from 5^th^ to 18^th^ of gestational week.

Fold change in gene expression level as estimated between weeks 5 and 18 of gestational age.

For the genes selected for further experiments TaqMan probe sets used in RT-qPCR experiments are provided.

| **Gene symbol** | **Gene name** | **Affymetrix probe** | **FDR-corrected**  ***P*-value** | **Fold change^a^ from 5 weeks to 18 weeks** | **TaqMan probe ID for genes entering RT-qPCR** |
| --- | --- | --- | --- | --- | --- |
| *FST* | Follistatin | 226847_AT | 0.00678 | 7.16 | Hs00246256_m1 |
| *PUM1* | Pumilio homolog 1 (Drosophila) | 201166_S_AT | 0.00891 | 1.32 | Hs00206469_m1 |
| *FST* | Follistatin | 204948_S_AT | 0.0330 | 3.25 | Hs00246256_m1 |
| *ITGBL1* | Integrin, beta-like 1 (with EGF-like repeat domains) | 214927_AT | 0.0366 | 6.06 | Hs00191224_m1 |
| *LYPD6* | LY6/PLAUR domain containing 6 | 227764_AT | 0.0366 | 3.46 | Hs00827109_m1 |
| *NR3C1* | Nuclear receptor subfamily 3, group C, member 1 (glucocorticoid receptor) | 201865_X_AT | 0.0366 | 2.69 | Hs00230818_m1 |
| *NR3C1* | Nuclear receptor subfamily 3, group C, member 1 (glucocorticoid receptor) | 211671_S_AT | 0.0366 | 2.68 | Hs00230818_m1 |
| *RSF1* | Remodeling and spacing factor 1 | 222541_AT | 0.0366 | 1.69 | Hs00213155_m1 |
| *NRCAM* | Neuronal cell adhesion molecule | 204105_S_AT | 0.0367 | 9.06 | Hs00170554_m1 |
| *CCDC115* | Coiled-coil domain containing 115 | 224946_S_AT | 0.0484 | 0.66 | Hs00260623_m1 |
| *SLC16A10* | Solute carrier family 16, member 10 (aromatic amino acid transporter) | 219915_S_AT | 0.0486 | 7.11 | Hs01039921_m1 |
| *GPR183*  *(EBI2)* | G protein-coupled receptor 183 / Epstein-Barr virus induced gene 2 | 205419_AT | 0.0486 | 6.96 | Hs00953886_m1 |
| *NR3C1* | Nuclear receptor subfamily 3, group C, member 1 (glucocorticoid receptor) | 216321_S_AT | 0.0486 | 3.32 | Hs00230818_m1 |
| *BACH1* | BTB and CNC homology 1, basic leucine zipper transcription factor 1 | 204194_AT | 0.0486 | 2.64 | Hs00895421_m1 |
| *LOC131185* | RAD23 homolog B (S. cerevisiae) pseudogene | 214422_AT | 0.0486 | 2.45 | N/A |
| *LYPD6* | LY6/PLAUR domain containing 6 | 227763_AT | 0.0486 | 1.67 | Hs00827109_m1 |
| *BCKDK* | Branched chain ketoacid dehydrogenase kinase | 202030_AT | 0.0486 | 0.66 | Hs00195380_m1 |
| *GMPPB* | GDP-mannose pyrophosphorylase B | 219920_S_AT | 0.0486 | 0.57 |  |
| *C2ORF18* | Chromosome 2 open reading frame 18 | 225695_AT | 0.0486 | 0.52 | Hs00215396_m1 |
| *GGPS1* | Geranylgeranyl diphosphate synthase 1 | 202321_AT | 0.0486 | 0.51 | Hs00191442_m1 |
| *SNX18* | Sorting nexin 18 | 226683_AT | 0.0502 | 2.1 | Hs00967869_m1 |
| *ATP8B4* | ATPase, class I, type 8B, member 4 | 220416_AT | 0.0521 | 4.69 |  |
| *SETD2* | SET domain containing 2 | 220946_S_AT | 0.0521 | 1.32 |  |
| *POLR3H* | Polymerase (RNA) III (DNA directed) polypeptide H (22.9kD) | 225682_S_AT | 0.0521 | 0.7 |  |
| *MAGEA10* | Melanoma antigen family A, 10 | 210295_AT | 0.0524 | 5.54 |  |
| *NEDD9* | Neural precursor cell expressed, developmentally down-regulated 9 | 202149_AT | 0.0524 | 4.92 | Hs00610590_m1 |
| *C7* | Complement component 7 | 202992_AT | 0.0524 | 4.92 |  |
| *BST2* | Bone marrow stromal cell antigen 2 | 201641_AT | 0.0524 | 3.41 |  |
| *HS3ST3A1* | Heparan sulfate (glucosamine) 3-O-sulfotransferase 3A1 | 219985_AT | 0.0524 | 3.29 |  |
| *PDE3B* | Phosphodiesterase 3B, cGMP-inhibited | 214582_AT | 0.0524 | 2.08 |  |
| *MORC3* | MORC family CW-type zinc finger 3 | 213000_AT | 0.0524 | 1.85 |  |
| *NUP153* | Nucleoporin 153kDa | 239948_AT | 0.0524 | 1.74 |  |
| *ZNF271* | Zinc finger protein 271 | 236231_AT | 0.0524 | 0.67 |  |
| *C9ORF80* | SOSS complex subunit C | 223558_AT | 0.0533 | 0.72 |  |
| *SLC25A15* | Solute carrier family 25 (mitochondrial carrier; ornithine transporter) member 15 | 218653_AT | 0.0533 | 0.22 |  |
| *C11ORF83* | UPF0723 protein C11orf83 Precursor | 229099_AT | 0.0543 | 0.64 |  |
| *AMOTL2* | Angiomotin like 2 | 203002_AT | 0.0548 | 2.43 |  |
| *CMKLR1* | Chemokine-like receptor 1 | 229121_AT | 0.0556 | 2.39 |  |
| *MTUS1* | Microtubule associated tumor suppressor 1 | 212096_S_AT | 0.0556 | 1.37 |  |
| *SORBS2* | Sorbin and SH3 domain containing 2 | 225728_AT | 0.0569 | 6.32 |  |
| *IRF2BP2* | Interferon regulatory factor 2 binding protein 2 | 224570_S_AT | 0.0569 | 1.95 |  |
| *RRAD* | Ras-related associated with diabetes | 204803_S_AT | 0.0575 | 4.23 |  |
| *NR3C1* | Nuclear receptor subfamily 3, group C, member 1 (glucocorticoid receptor) | 201866_S_AT | 0.0575 | 1.93 | Hs00230818_m1 |
| *C4ORF31* | Fibronectin type-III domain-containing protein C4orf31 Precursor | 219747_AT | 0.0624 | 6.02 |  |
| *TRIM16* | Tripartite motif-containing 16 | 204341_AT | 0.0624 | 3.23 |  |
| *NALCN* | Sodium leak channel, non-selective | 228608_AT | 0.0624 | 2.48 |  |
| *CCNG2* | Cyclin G2 | 202769_AT | 0.0624 | 2.38 | Hs00171119_m1 |
| *ECM1* | Extracellular matrix protein 1 | 209365_S_AT | 0.0624 | 1.83 |  |
| *BCKDHA* | Branched chain keto acid dehydrogenase E1, alpha polypeptide | 202331_AT | 0.0630 | 0.74 |  |
| *STUB1* | STIP1 homology and U-box containing protein 1 | 217934_X_AT | 0.0630 | 0.67 |  |
| *CDK17* | Cyclin-dependent kinase 17 | 221918_AT | 0.0635 | 2.13 |  |
| *ABCA1* | ATP-binding cassette, sub-family A (ABC1), member 1 | 203504_S_AT | 0.0635 | 2.1 |  |
| *HSD17B2* | Hydroxysteroid (17-beta) dehydrogenase 2 | 204818_AT | 0.0650 | 16.11 |  |
| *BMP5* | Bone morphogenetic protein 5 | 205431_S_AT | 0.0650 | 13.36 | Hs00234930_m1 |
| *PDLIM5* | PDZ and LIM domain 5 | 213684_S_AT | 0.0650 | 7.06 |  |
| *ZFP36L1* | Zinc finger protein 36, C3H type-like 1 | 211965_AT | 0.0650 | 5.62 | Hs00245183_m1 |
| *PLSCR4* | Phospholipid scramblase 4 | 218901_AT | 0.0650 | 3.32 |  |
| *NRP2* | Neuropilin 2 | 211844_S_AT | 0.0650 | 2.87 |  |
| *SRGAP2P1* | SLIT-ROBO Rho GTPase activating protein 2 pseudogene 1 | 229067_AT | 0.0650 | 2.06 |  |
| *PHF3* | PHD finger protein 3 | 217954_S_AT | 0.0650 | 2.06 |  |
| *FBXO8* | F-box protein 8 | 223240_AT | 0.0650 | 2.06 |  |
| *CLPX* | ClpX caseinolytic peptidase X homolog (E. coli) | 204809_AT | 0.0650 | 1.88 |  |
| *PDCD5* | Programmed cell death 5 | 227751_AT | 0.0650 | 1.6 |  |
| *CRCP* | CGRP receptor component | 203899_S_AT | 0.0650 | 0.71 |  |
| *EXOSC4* | Exosome component 4 | 58696_AT | 0.0650 | 0.69 |  |
| *HDLBP* | High density lipoprotein binding protein | 221767_X_AT | 0.0650 | 0.66 |  |
| *PAEP* | Progestagen-associated endometrial protein | 206859_S_AT | 0.0650 | 0.01 |  |
| *LIPT1* | Lipoyltransferase 1 | 205571_AT | 0.0665 | 0.33 |  |
| *PLAGL1* | Pleiomorphic adenoma gene-like 1 | 207943_X_AT | 0.0682 | 2.71 | Hs00414677_m1 |
| *SPRYD4* | SPRY domain containing 4 | 225616_AT | 0.0713 | 0.63 |  |
| *LILRB5* | Leukocyte immunoglobulin-like receptor, subfamily B (with TM and ITIM domains), member 5 | 206856_AT | 0.0714 | 3.01 |  |
| *KCNJ2* | Potassium inwardly-rectifying channel, subfamily J, member 2 | 206765_AT | 0.0719 | 10.41 |  |
| *ENPP2* | Ectonucleotide pyrophosphatase/phosphodiesterase 2 | 210839_S_AT | 0.0719 | 6.06 |  |
| *ANKRD50* | Ankyrin repeat domain 50 | 225731_AT | 0.0750 | 3.71 |  |
| *ANKS1A* | Ankyrin repeat and sterile alpha motif domain containing 1A | 212747_AT | 0.0750 | 3.36 |  |
| *SLC35B2* | Solute carrier family 35, member B2 | 224716_AT | 0.0750 | 0.63 |  |
| *UROS* | Uroporphyrinogen III synthase | 203031_S_AT | 0.0773 | 0.50 |  |
| *SLC16A10* | Solute carrier family 16, member 10 (aromatic amino acid transporter) | 222939_S_AT | 0.0785 | 15.78 | Hs01039921_m1 |
| *GATM* | Glycine amidinotransferase (L-arginine:glycine amidinotransferase) | 203178_AT | 0.0785 | 7.84 | Hs00155208_m1 |
| *TSHZ1* | Teashirt zinc finger homeobox 1 | 223282_AT | 0.0785 | 3.78 |  |
| *TTC9* | Tetratricopeptide repeat domain 9 | 213172_AT | 0.0785 | 0.63 |  |
| *BMP5* | Bone morphogenetic protein 5 | 205430_AT | 0.0786 | 58.89 | Hs00234930_m1 |
| *MS4A4A* | Membrane-spanning 4-domains, subfamily A, member 4 | 224357_S_AT | 0.0789 | 3.16 |  |
| *MAF1* | MAF1 homolog (S. cerevisiae) | 222998_AT | 0.0789 | 0.75 |  |
| *POLR2I* | Polymerase (RNA) II (DNA directed) polypeptide I, 14.5kDa | 212955_S_AT | 0.0789 | 0.47 |  |
| *ENPP2* | Ectonucleotide pyrophosphatase/phosphodiesterase 2 | 209392_AT | 0.0801 | 6.32 |  |
| *MLXIP* | MLX interacting protein | 211789_S_AT | 0.0801 | 0.77 |  |
| *N4BP1* | NEDD4 binding protein 1 | 32069_AT | 0.0801 | 0.71 |  |
| *CEP70* | Centrosomal protein 70kDa | 219036_AT | 0.0801 | 0.49 |  |
| *SRPRB* | Signal recognition particle receptor, B subunit | 218140_X_AT | 0.0822 | 0.48 |  |
| *CCNG2* | Cyclin G2 | 202770_S_AT | 0.0824 | 2.5 | Hs00171119_m1 |
| *TMEM154* | Transmembrane protein 154 | 238063_AT | 0.0824 | 0.43 |  |
| *LYVE1* | Lymphatic vessel endothelial hyaluronan receptor 1 | 220037_S_AT | 0.0833 | 25.99 |  |
| *STC1* | Stanniocalcin 1 | 204595_S_AT | 0.0833 | 6.92 | Hs00174970_m1 |
| *NEDD9* | Neural precursor cell expressed, developmentally down-regulated 9 | 202150_S_AT | 0.0833 | 4.38 | Hs00610590_m1 |
| *INMT* | Indolethylamine N-methyltransferase | 224061_AT | 0.0833 | 3.81 |  |
| *COLEC11* | Collectin sub-family member 11 | 219873_AT | 0.0833 | 0.25 |  |
| *RNF111* | Ring finger protein 111 | 218761_AT | 0.084 | 1.54 |  |
| *ANKRD50* | Ankyrin repeat domain 50 | 225735_AT | 0.0878 | 3.51 |  |
| *AHSA2* | AHA1, activator of heat shock 90kDa protein ATPase homolog 2 (yeast) | 230148_AT | 0.0878 | 1.53 |  |
| *IQGAP2* | IQ motif containing GTPase activating protein 2 | 203474_AT | 0.0880 | 6.96 |  |
| *SESN1* | Sestrin 1 | 218346_S_AT | 0.0880 | 3.34 |  |
| *FYB* | FYN binding protein (FYB-120/130) | 211794_AT | 0.0880 | 1.57 |  |
| *PKP1* | Plakophilin 1 (ectodermal dysplasia/skin fragility syndrome) | 221854_AT | 0.0880 | 1.52 |  |
| *PREX1* | Phosphatidylinositol-3,4,5-trisphosphate-dependent Rac exchange factor 1 | 224925_AT | 0.0880 | 1.46 |  |
| *HUNK* | Uormonally up-regulated Neu-associated kinase | 219535_AT | 0.0880 | 1.39 |  |
| *DNAJC16* | DnaJ (Hsp40) homolog, subfamily C, member 16 | 212908_AT | 0.0880 | 0.60 |  |
| *STUB1* | STIP1 homology and U-box containing protein 1 | 233049_X_AT | 0.0880 | 0.57 |  |
| *NF2* | Neurofibromin 2 (merlin) | 218915_AT | 0.0880 | 0.49 |  |
| *CLDN10* | Claudin 10 | 205328_AT | 0.0880 | 0.06 |  |
| *BAG1* | BCL2-associated athanogene | 202387_AT | 0.089 | 0.55 |  |
| *MEG3* | Maternally expressed 3 (non-protein coding) | 235077_AT | 0.0908 | 10.63 | Hs00292028_m1 |
| *CYTL1* | Cytokine-like 1 | 219837_S_AT | 0.0908 | 5.98 |  |
| *NFASC* | Neurofascin homolog (chicken) | 213438_AT | 0.0908 | 4.69 |  |
| *SNAP23* | Synaptosomal-associated protein, 23kDa | 229773_AT | 0.0908 | 3.76 |  |
| *SORBS2* | Sorbin and SH3 domain containing 2 | 204288_S_AT | 0.0908 | 3.66 |  |
| *STC1* | Stanniocalcin 1 | 204596_S_AT | 0.0908 | 2.57 | Hs00174970_m1 |
| *PECAM1* | Platelet/endothelial cell adhesion molecule | 208981_AT | 0.0908 | 2.53 |  |
| *ATP12A* | ATPase, H+/K+ transporting, nongastric, alpha polypeptide | 207367_AT | 0.0908 | 2.5 |  |
| *SORBS2* | Sorbin and SH3 domain containing 2 | 233720_AT | 0.0908 | 2.46 |  |
| *PDLIM5* | PDZ and LIM domain 5 | 216804_S_AT | 0.0908 | 2.39 |  |
| *GRINL1A* | Glutamate receptor, ionotropic, N-methyl D-aspartate-like 1A | 228568_AT | 0.0908 | 2.33 |  |
| *C7ORF30* | Chromosome 7 open reading frame 30 | 230516_AT | 0.0908 | 2.01 |  |
| *DRAM2* | DNA-damage regulated autophagy modulator 2 | 225230_AT | 0.0908 | 1.85 |  |
| *MAML2* | Mastermind-like 2 (Drosophila) | 235106_AT | 0.0908 | 1.85 |  |
| *FOXO3* | Forkhead box O3 | 224891_AT | 0.0908 | 1.57 |  |
| *BOD1L* | Biorientation of chromosomes in cell division 1-like | 225821_S_AT | 0.0908 | 1.33 |  |
| *GLRX3* | Glutaredoxin 3 | 214205_X_AT | 0.0908 | 0.75 |  |
| *MCCC2* | Methylcrotonoyl-Coenzyme A carboxylase 2 (beta) | 209623_AT | 0.0908 | 0.61 |  |
| *RP11-500G10.4* | N/A | 229323_AT | 0.0908 | 0.58 |  |
| *CD36* | CD36 molecule (thrombospondin receptor) | 206488_S_AT | 0.0924 | 17.51 |  |
| *SNORD113* | Small nucleolar RNA SNORD113/SNORD114 family | 232355_AT | 0.0924 | 6.45 |  |
| *PLAGL1* | Pleiomorphic adenoma gene-like 1 | 207002_S_AT | 0.0924 | 3.39 | Hs00414677_m1 |
| *CADM3* | Cell adhesion molecule 3 | 213948_X_AT | 0.0924 | 2.57 |  |
| *PDGFC* | Platelet derived growth factor C | 218718_AT | 0.0924 | 2.36 |  |
| *EED* | Embryonic ectoderm development | 210656_AT | 0.0934 | 2.19 |  |
| *AP3M2* | Adaptor-related protein complex 3, mu 2 subunit | 203410_AT | 0.0935 | 1.67 |  |
| *ARID2* | AT rich interactive domain 2 (ARID, RFX-like) | 225486_AT | 0.094 | 1.68 |  |
| *LRCH2* | Leucine-rich repeats and calponin homology (CH) domain containing 2 | 227688_AT | 0.0943 | 5.7 |  |
| *LYVE1* | Lymphatic vessel endothelial hyaluronan receptor 1 | 219059_S_AT | 0.0947 | 17.39 |  |
| *NCF2* | Neutrophil cytosolic factor 2 | 209949_AT | 0.0947 | 5.66 |  |
| *TTYH2* | Tweety homolog 2 (Drosophila) | 223741_S_AT | 0.0947 | 4.86 |  |
| *KDR* | Kinase insert domain receptor (a type III receptor tyrosine kinase) | 203934_AT | 0.0947 | 3.27 |  |
| *HYMAI* | Hydatidiform mole associated and imprinted (non-protein coding) | 215513_AT | 0.0947 | 3.25 |  |
| *MEG3* | Maternally expressed 3 (non-protein coding) | 229557_AT | 0.0947 | 3.14 | Hs00292028_m1 |
| *PPM1D* | Protein phosphatase 1D magnesium-dependent, delta isoform | 204566_AT | 0.0947 | 3.01 |  |
| *IRS1* | Insulin receptor substrate 1 | 204686_AT | 0.0947 | 2.95 |  |
| *MAML2* | Mastermind-like 2 (Drosophila) | 235457_AT | 0.0947 | 2.81 |  |
| *INSIG1* | Insulin induced gene 1 | 201625_S_AT | 0.0947 | 2.66 |  |
| *ITGBL1* | Integrin, beta-like 1 (with EGF-like repeat domains) | 231993_AT | 0.0947 | 2.23 | Hs00191224_m1 |
| *FCHSD2* | FCH and double SH3 domains 2 | 203620_S_AT | 0.0947 | 2.23 |  |
| *PIK3AP1* | Phosphoinositide-3-kinase adaptor protein 1 | 226459_AT | 0.0947 | 2.16 |  |
| *NINJ1* | Ninjurin 1 | 203045_AT | 0.0947 | 1.89 |  |
| *NFASC* | Neurofascin homolog (chicken) | 214799_AT | 0.0947 | 1.89 |  |
| *TBX5* | T-box 5 | 207155_AT | 0.0947 | 1.88 |  |
| *SLC15A3* | Solute carrier family 15, member 3 | 219593_AT | 0.0947 | 1.77 |  |
| *PRLR* | Prolactin receptor | 216638_S_AT | 0.0947 | 1.57 |  |
| *PDGFB* | Platelet-derived growth factor beta polypeptide (simian sarcoma viral (v-sis) oncogene homolog) | 216061_X_AT | 0.0947 | 1.55 |  |
| *TNKS* | Tankyrase, TRF1-interacting ankyrin-related ADP-ribose polymerase | 216695_S_AT | 0.0947 | 1.31 |  |
| *TMEM41A* | Transmembrane protein 41A | 235037_AT | 0.0947 | 0.75 |  |
| *POLR3C* | Polymerase (RNA) III (DNA directed) polypeptide C (62kD) | 210573_S_AT | 0.0947 | 0.72 |  |
| *NDUFB11* | NADH dehydrogenase (ubiquinone) 1 beta subcomplex, 11, 17.3kDa | 218320_S_AT | 0.0947 | 0.64 |  |
| *BCL7C* | microRNA 762 | 219072_AT | 0.0947 | 0.61 |  |
| *CMTM4* | CKLF-like MARVEL transmembrane domain containing 4 | 224998_AT | 0.0947 | 0.60 |  |
| *TAF10* | TAF10 RNA polymerase II, TATA box binding protein (TBP)-associated factor, 30kDa | 200055_AT | 0.0947 | 0.57 |  |
| *MSTO1* | Misato homolog 1 (Drosophila) | 218296_X_AT | 0.0947 | 0.50 |  |
| *PMS2L5* | Postmeiotic segregation increased 2 pseudogene 5 | 242201_AT | 0.0947 | 0.38 |  |
| *FKBP11* | FK506 binding protein 11, 19 kDa | 219118_AT | 0.0947 | 0.30 |  |
| *C20ORF112* | Uncharacterized protein C20orf112 | 225224_AT | 0.0952 | 2.64 |  |
| *TSTA3* | Tissue specific transplantation antigen P35B | 201644_AT | 0.0957 | 0.7 |  |
| *CDH11* | Cadherin 11, type 2, OB-cadherin (osteoblast) | 236179_AT | 0.0975 | 3.14 | Hs00156438_m1 |
| *GATM* | Glycine amidinotransferase (L-arginine:glycine amidinotransferase) | 216733_S_AT | 0.0976 | 9.32 | Hs00155208_m1 |
| *MEG3* | Maternally expressed 3 (non-protein coding) | 212732_AT | 0.0976 | 8.28 | Hs00292028_m1 |
| *ZFP36L1* | Zinc finger protein 36, C3H type-like 1 | 211962_S_AT | 0.0976 | 1.85 | Hs00245183_m1 |
| *YTHDC1* | YTH domain containing 1 | 214814_AT | 0.098 | 1.79 |  |
| *SERP1* | Stress-associated endoplasmic reticulum protein 1 | 200971_S_AT | 0.098 | 0.7 |  |
| *FKBP11* | FK506 binding protein 11 | 219117_S_AT | 098 | 0.34 |  |
| *LOC100130097* | Kinesin-like protein family member 6-like | 241376_AT | 0.0980 | 0.17 |  |
| *HSPA6* | Heat shock 70kDa protein 7 (HSP70B) | 213418_AT | 0.0983 | 9.71 |  |
| *GGA2* | Golgi associated, gamma adaptin ear containing, ARF binding protein 2 | 208914_AT | 0.0983 | 0.46 |  |
