# Supplementary material for: Mid-Gestational Gene Expression Profile in Placenta and Link to Pregnancy Complications
Source: PLoS One. 2012 Nov 7;7(11):e49248. doi: 10.1371/journal.pone.0049248 (PMC3492272; doi:10.1371/journal.pone.0049248)
Supplement: Table S3 — Biological processes obtained from Gene Ontology Annotation Database ( www.ebi.ac.uk/GOA/ ) for genes exhibiting significantly increased or decreased placental expression in the progress of pregnancy from 5th to 18th of gestational week (ANOVA, FDR P <0.1) and selected for RT-qPCR confirmation. (DOCX) [file pone.0049248.s012.docx]

**Table S3.** Biological processes obtained from Gene Ontology Annotation Database ([www.ebi.ac.uk/GOA/](http://www.ebi.ac.uk/GOA/)) for genes exhibiting significantly increased or decreased placental expression in the progress of pregnancy from 5^th^ to 18^th^ of gestational week (ANOVA, FDR *P*<0.1) and selected for RT-qPCR confirmation.

| **Gene ID** | **Gene name** | **Biological process** |
| --- | --- | --- |
| *BACH1* | *BTB and CNC homology 1, basic leucine zipper transcription factor 1* | DNA-dependent regulation of transcription |
| *BCKDK* | *Branched chain ketoacid dehydrogenase kinase* | protein phosphorylation; branched chain family amino acid catabolic process |
| *BMP5* | *Bone morphogenetic protein 5* | skeletal system development; ossification; pattern specification process; male genitalia development; growth; cartilage development |
| *C2orf18* | *Chromosome 2 open reading frame 18* | carbohydrate transport; |
| *CCDC115* | *Coiled-coil domain containing 115* | Uncharacterized |
| *CCNG2* | *Cyclin G2* | cell cycle regulation; mitosis |
| *CDH11* | *Cadherin 11* | Homophilic cell adhesion; cell adhesion |
| *FST* | *Follistatin* | gamete generation; negative regulation of follicle-stimulating hormone secretion; female gonad development; hair follicle development; keratinocyte proliferation; odontogenesis of dentine-containing tooth; BMP signaling pathway; activin receptor signaling pathway; hemopoietic progenitor cell differentiation; negative regulation of transcription from RNA polymerase II promoter; pattern specification process; |
| *GATM* | *Glycine amidinotransferase (L-arginine:glycine amidinotransferase)* | embryo development; creatine metabolic and biosynthetic process; tissue regeneration; response to oxidative stress, nutrient, organic substance, peptide hormone stimulus, mercury ion; cellular nitrogen compound metabolic process |
| *GGPS1* | *Geranylgeranyl diphosphate synthase 1* | isoprenoid biosynthetic process |
| *GPR183*/ *EBI2* | *G protein-coupled receptor 183 / Epstein-Barr virus induced gene 2* | humoral immune response; mature B cell differentiation; G-protein coupled purinergic nucleotide receptor signaling pathway |
| *ITGBL1* | *Integrin, beta-like 1 (with EGF-like repeat domains)* | cell adhesion; cell-matrix adhesion; integrin-mediated signaling pathway |
| *LYPD6* | *LY6/PLAUR domain containing 6* | uncharacterized |
| *MEG3* | *Maternally expressed 3 (non-protein coding)* | negative regulation of cell proliferation, angiogenesis and DNA biosynthesis; negative regulation of vascular endothelial growth factor receptor signaling and Notch signaling pathways; positive regulation of embryonic development, DNA-dependent transcription and skeletal muscle fiber development; axon guidance; cell differentiation; gonadotropin secretion; RNA folding; hypomethylation of CpG island |
| *NEDD9* | *Neural precursor cell expressed, developmentally down-regulated 9* | cytoskeleton organization; actin filament bundle assembly; cell cycle; mitosis; cell adhesion; integrin-mediated signaling pathway; regulation of growth |
| *NR3C1* | *Nuclear receptor subfamily 3, group C, member 1 (glucocorticoid receptor)* | DNA-dependent transcription and regulation of transcription; glucocorticoid receptor signaling pathway; glucocorticoid mediated signaling pathway |
| *NRCAM* | *Neuronal cell adhesion molecule* | central nervous system development; protein localization; cell-cell adhesion; neuronal action potential propagation; axon guidance |
| *PLAGL1* | *Pleiomorphic adenoma gene-like 1* | induction of apoptosis; cell cycle arrest; DNA-dependent regulation of transcription from RNA polymerase II promoter; |
| *PUM1* | *Pumilio homolog 1 (Drosophila)* | regulation of translation; post-Golgi vesicle-mediated transport; cellular membrane organization |
| *RSF1* | *Remodeling and spacing factor 1* | nucleosome assembly and positioning; CenH3-containing nucleosome assembly at centromere; chromatin modification; regulation of viral transcription; DNA-dependent transcription and transcription regulation; transcription initiation; negative regulation of DNA binding |
| *SLC16A10* | *Solute carrier family 16, member 10* (aromatic amino acid transporter) | transmembrane transport |
| *SNX18* | *Sorting nexin 18* | endocytosis; cell communication; protein transport; regulation of GTPase activity |
| *STC1* | *Stanniocalcin 1* | Ossification; embryo implantation; decidualization; cellular response to vitamin D, cAMP and hypoxia |
| *ZFP36L1* | *Zinc finger protein 36, C3H type-like 1* | vasculogenesis; T cell differentiation in thymus; nuclear-transcribed mRNA catabolic process, deadenylation-dependent decay; DNA-dependent transcription; regulation of translation; RNA metabolic process; regulation of mRNA stability |
