# Supplementary material for: Mid-Gestational Gene Expression Profile in Placenta and Link to Pregnancy Complications
Source: PLoS One. 2012 Nov 7;7(11):e49248. doi: 10.1371/journal.pone.0049248 (PMC3492272; doi:10.1371/journal.pone.0049248)
Supplement: Table S4 — Differentially expressed placental genes on Affymetrix® GeneChip detected by group-based microchip analysis comparing the first ( n = 6; gestational days 38, 55, 2×56, 81, 91) and the second ( n = 4; gestational days 120, 121, 126, 132) trimester discovery samples. (DOCX) [file pone.0049248.s013.docx]

**Table S4.** Differentially expressed placental genes on Affymetrix® GeneChip detected by group-based microchip analysis comparing the first (*n*=6; gestational days 38, 55, 2x56, 81, 91) and the second (*n*=4; gestational days 120, 121, 126, 132) trimester discovery samples.

229 genes (268 probe sets) identified on Affymetrics® GeneChip exhibiting statistically significant (empirical Bayes moderated t-test, FDR-corrected *P*-value<0.05) or suggestive (*P*-value<0.1) increased or decreased placental expression.

Fold change was calculated between median expression values of early and mid-gestation study groups. Bold letters indicate to genes that had been also identified with statistically significant change (FDR *P*<0.1; **Table S2**) in transcript levels in the ANOVA analysis of the microchip data. ANOVA was based on time-dependent model of gene expression across all the 10 discovery samples from gestational days 38 to 132.

| **Gene symbol** | **Affymetrix probe** | **FDR corrected *P*-value** | **Fold change** |
| --- | --- | --- | --- |
| C8orf4 | 218541_s_at | 0.0035 | 6.24 |
| LOC100130070 /// LOC100130775 /// LOC100131787 /// LOC100131905 /// LOC100132291 /// LOC100132488 /// RPS27 | 236621_at | 0.0035 | 1.97 |
| LRP6 | 225745_at | 0.0035 | 2.52 |
| ICAM2 | 213620_s_at | 0.0073 | 2.38 |
| **MORC3** | 213000_at | 0.012 | 1.71 |
| **C4orf31** | 219747_at | 0.017 | 4.23 |
| CYR61 | 201289_at | 0.017 | 6.42 |
| **EED** | 210656_at | 0.017 | 2.27 |
| PDLIM3 | 210170_at | 0.017 | 7.20 |
| ATF7IP | 216197_at | 0.019 | 1.68 |
| **CMKLR1** | 229121_at | 0.019 | 2.18 |
| ICAM2 | 204683_at | 0.019 | 2.00 |
| RAB3GAP2 | 243851_at | 0.021 | 1.94 |
| SOX7 | 224013_s_at | 0.021 | 3.34 |
| SPRY1 | 212558_at | 0.021 | 3.39 |
| TCF4 | 213891_s_at | 0.021 | 2.27 |
| CYR61 | 210764_s_at | 0.022 | 6.02 |
| **BST2** | 201641_at | 0.023 | 2.61 |
| **NUP153** | 239948_at | 0.025 | 1.55 |
| SETBP1 | 205933_at | 0.025 | 2.11 |
| **GCOM1 /// GRINL1A** | 228568_at | 0.026 | 1.94 |
| HEBP2 | 203430_at | 0.026 | 0.63 |
| NAMPT | 243296_at | 0.026 | 6.54 |
| NR2F1 | 209505_at | 0.026 | 2.43 |
| **ZFP36L1** | 211965_at | 0.026 | 3.39 |
| **ANKRD50** | 225735_at | 0.028 | 2.37 |
| CHMP4C | 226803_at | 0.028 | 0.69 |
| SOX7 | 228698_at | 0.028 | 6.54 |
| **CYTL1** | 219837_s_at | 0.028 | 4.38 |
| SDPR | 222717_at | 0.028 | 2.59 |
| **INMT** | 224061_at | 0.029 | 2.57 |
| SNRK | 209481_at | 0.029 | 1.81 |
| **PCTK2** | 221918_at | 0.029 | 1.90 |
| TJP1 | 202011_at | 0.029 | 1.61 |
| C20orf74 | 239660_at | 0.030 | 1.93 |
| CPNE8 | 228365_at | 0.030 | 1.95 |
| **FST** | 226847_at | 0.030 | 3.40 |
| **HS3ST3A1** | 219985_at | 0.030 | 2.15 |
| **HSPA6** | 213418_at | 0.030 | 7.68 |
| IFIT2 | 226757_at | 0.030 | 2.17 |
| **KDR** | 203934_at | 0.030 | 2.51 |
| **LOC131185 /// RAD23B** | 214422_at | 0.030 | 1.77 |
| **PDLIM5** | 213684_s_at | 0.030 | 4.96 |
| RCAN1 | 208370_s_at | 0.030 | 4.22 |
| SETD5 | 244691_at | 0.030 | 1.67 |
| SFRS15 | 222310_at | 0.030 | 1.60 |
| TCF4 | 203753_at | 0.030 | 2.45 |
| TNFSF13B | 223502_s_at | 0.030 | 2.98 |
| WRNIP1 | 230847_at | 0.030 | 1.87 |
| GADD45B | 207574_s_at | 0.030 | 5.06 |
| SAP30L | 232135_at | 0.030 | 0.62 |
| **AMOTL2** | 203002_at | 0.030 | 1.81 |
| **PECAM1** | 208982_at | 0.031 | 3.93 |
| GLS | 223079_s_at | 0.031 | 1.78 |
| CHI3L1 | 209396_s_at | 0.032 | 0.65 |
| **GPR183** | 205419_at | 0.032 | 3.46 |
| **CDH11** | 239769_at | 0.033 | 2.10 |
| CDS2 | 233630_at | 0.033 | 1.59 |
| GBP1 | 231578_at | 0.033 | 1.59 |
| TCF4 | 212385_at | 0.033 | 2.05 |
| **ATP8B4** | 220416_at | 0.033 | 3.27 |
| KIAA0485 | 214295_at | 0.033 | 1.94 |
| **PECAM1** | 208983_s_at | 0.034 | 2.68 |
| **SNORD114-3** | 232355_at | 0.034 | 3.80 |
| **ANKRD50** | 225731_at | 0.034 | 2.56 |
| **PECAM1** | 208981_at | 0.034 | 2.47 |
| **IRS1** | 204686_at | 0.035 | 2.56 |
| TCF4 | 222146_s_at | 0.036 | 2.40 |
| **BACH1** | 204194_at | 0.036 | 1.80 |
| **C11orf83** | 229099_at | 0.036 | 0.68 |
| **C2orf18** | 225695_at | 0.036 | 0.67 |
| CDH5 | 204677_at | 0.036 | 2.95 |
| **LYPD6** | 227763_at | 0.036 | 1.50 |
| MINPP1 | 209585_s_at | 0.036 | 1.90 |
| **NEDD9** | 202149_at | 0.036 | 2.73 |
| OTUB2 | 219369_s_at | 0.036 | 0.57 |
| PDPN | 221898_at | 0.036 | 2.56 |
| SETBP1 | 227478_at | 0.036 | 2.66 |
| **YTHDC1** | 214814_at | 0.036 | 1.64 |
| FOS | 209189_at | 0.037 | 8.48 |
| PHLDA1 | 217996_at | 0.037 | 4.05 |
| STOM | 201060_x_at | 0.037 | 1.57 |
| **SETD2** | 241458_at | 0.040 | 1.65 |
| VEZF1 | 202173_s_at | 0.042 | 1.49 |
| APAF1 | 204859_s_at | 0.043 | 1.54 |
| EXOC6 | 226259_at | 0.044 | 2.28 |
| **ZFP36L1** | 211962_s_at | 0.044 | 1.79 |
| CIRBP | 200811_at | 0.044 | 0.58 |
| SENP6 | 214790_at | 0.044 | 2.09 |
| IMPA2 | 203126_at | 0.044 | 0.46 |
| MOSC2 | 221636_s_at | 0.044 | 2.20 |
| PDGFA | 205463_s_at | 0.044 | 1.60 |
| **SRGAP2P1** | 229067_at | 0.044 | 1.82 |
| TRIB1 | 202241_at | 0.044 | 2.08 |
| ZNF333 | 231369_at | 0.044 | 1.59 |
| **ITGBL1** | 214927_at | 0.045 | 3.22 |
| TCF4 | 212386_at | 0.045 | 2.12 |
| TIE1 | 204468_s_at | 0.045 | 1.95 |
| FLJ43663 | 228702_at | 0.045 | 3.26 |
| PRKG1 | 228396_at | 0.046 | 1.70 |
| **SLC16A10** | 219915_s_at | 0.047 | 3.93 |
| CCL2 | 216598_s_at | 0.049 | 2.29 |
| PIK3CA | 204369_at | 0.049 | 1.54 |
| TCF4 | 212387_at | 0.049 | 2.01 |
| C9orf150 | 227443_at | 0.051 | 2.39 |
| JUN | 201464_x_at | 0.051 | 3.40 |
| **LYPD6** | 227764_at | 0.051 | 2.24 |
| RAPGEF5 | 204681_s_at | 0.051 | 4.03 |
| FAM133B /// LOC728153 /// LOC728408 | 235469_at | 0.051 | 1.54 |
| LRP6 | 205606_at | 0.051 | 1.67 |
| PELI1 | 232304_at | 0.051 | 2.41 |
| **POLR3H** | 225682_s_at | 0.051 | 0.75 |
| SAMD5 | 242626_at | 0.051 | 2.29 |
| SMNDC1 | 200071_at | 0.051 | 1.42 |
| TACC1 | 242290_at | 0.051 | 2.28 |
| FCGR2A | 203561_at | 0.051 | 3.52 |
| BAG2 | 209406_at | 0.052 | 1.73 |
| TMF1 | 242243_at | 0.054 | 1.58 |
| HSPA1A /// HSPA1B | 200799_at | 0.054 | 5.15 |
| CD28 | 206545_at | 0.057 | 2.90 |
| CREM | 209967_s_at | 0.057 | 2.38 |
| **PKP1** | 221854_at | 0.057 | 1.43 |
| EFNB2 | 202669_s_at | 0.057 | 1.95 |
| AP1S2 | 203300_x_at | 0.059 | 1.85 |
| C4orf32 | 227856_at | 0.059 | 2.72 |
| CREB1 | 225572_at | 0.059 | 1.42 |
| KANK3 | 213715_s_at | 0.059 | 2.05 |
| **SNX18** | 226683_at | 0.059 | 1.71 |
| **TRIM16** | 204341_at | 0.059 | 1.95 |
| UBE2D3 | 240383_at | 0.059 | 1.83 |
| **NR3C1** | 211671_s_at | 0.059 | 2.00 |
| **GATM** | 203178_at | 0.060 | 4.01 |
| **ANKS1A** | 212747_at | 0.060 | 1.98 |
| **ECM1** | 209365_s_at | 0.060 | 1.65 |
| PTK2 | 241453_at | 0.060 | 1.74 |
| HNRNPA1 /// LOC728844 | 222040_at | 0.061 | 2.08 |
| PHF10 | 225048_at | 0.061 | 1.65 |
| **NEDD9** | 202150_s_at | 0.062 | 2.41 |
| DYRK2 | 202968_s_at | 0.062 | 1.62 |
| ELAVL1 | 227746_at | 0.062 | 0.66 |
| **NR3C1** | 201865_x_at | 0.062 | 1.88 |
| **LILRB5** | 206856_at | 0.063 | 2.17 |
| MLL5 | 226100_at | 0.063 | 1.57 |
| C15orf5 | 208109_s_at | 0.064 | 2.23 |
| CASP1 | 211367_s_at | 0.064 | 2.21 |
| **HSD17B2** | 204818_at | 0.064 | 4.49 |
| SEPP1 | 229620_at | 0.064 | 2.30 |
| PRKAR1A | 242482_at | 0.064 | 2.17 |
| FBP1 | 209696_at | 0.066 | 0.64 |
| LRP6 | 34697_at | 0.066 | 1.50 |
| **NRCAM** | 204105_s_at | 0.066 | 4.23 |
| **PDE3B** | 214582_at | 0.066 | 1.58 |
| PTPN7 | 204852_s_at | 0.066 | 0.71 |
| SAMD5 | 228653_at | 0.066 | 1.94 |
| **SNAP23** | 229773_at | 0.066 | 2.45 |
| TLR8 | 229560_at | 0.066 | 2.77 |
| TPPP | 230104_s_at | 0.066 | 1.37 |
| ZBED1 | 203043_at | 0.066 | 0.65 |
| HLX | 214438_at | 0.066 | 2.16 |
| PDE3A | 206389_s_at | 0.066 | 1.36 |
| CXCL2 | 209774_x_at | 0.067 | 2.32 |
| **ITGBL1** | 231993_at | 0.067 | 1.88 |
| **PAEP** | 206859_s_at | 0.067 | 0.03 |
| PAPOLG | 222839_s_at | 0.067 | 1.75 |
| PCDH18 | 225977_at | 0.067 | 1.65 |
| IFIT3 | 229450_at | 0.068 | 2.23 |
| STK32B | 219686_at | 0.069 | 1.39 |
| PCDH12 | 219656_at | 0.070 | 1.60 |
| TACC1 | 217437_s_at | 0.070 | 1.51 |
| TAPT1 | 216373_at | 0.070 | 1.53 |
| **MS4A4A** | 224357_s_at | 0.071 | 2.47 |
| CREM | 214508_x_at | 0.071 | 2.53 |
| ELF2 | 210361_s_at | 0.071 | 1.53 |
| DDX26B | 227485_at | 0.072 | 1.46 |
| DPYSL3 | 201431_s_at | 0.076 | 1.87 |
| KIAA1033 | 212795_at | 0.076 | 1.31 |
| MYST3 | 226547_at | 0.076 | 1.44 |
| SPTBN1 | 226765_at | 0.076 | 1.57 |
| **MEG3** | 212732_at | 0.077 | 3.48 |
| AP1S2 | 230264_s_at | 0.078 | 1.95 |
| FAM53B | 203206_at | 0.078 | 0.73 |
| hCG_2004593 /// hCG_39912 /// LOC100129657 /// LOC100132742 /// LOC442232 /// LOC643863 /// LOC727984 /// LOC729340 /// LOC729349 /// RPL17 | 214291_at | 0.078 | 1.74 |
| LEPROTL1 | 202594_at | 0.078 | 1.94 |
| **NFASC** | 213438_at | 0.078 | 3.64 |
| **C7orf30** | 230516_at | 0.078 | 1.83 |
| ADORA3 | 223660_at | 0.078 | 3.99 |
| LOC646870 | 229364_at | 0.079 | 0.71 |
| CREM | 207630_s_at | 0.079 | 2.59 |
| hCG_2045899 | 238488_at | 0.079 | 1.92 |
| **IRF2BP2** | 224571_at | 0.079 | 1.74 |
| MGC15634 | 230534_at | 0.079 | 1.26 |
| **NR3C1** | 216321_s_at | 0.079 | 2.08 |
| PHLDA1 | 217997_at | 0.079 | 2.09 |
| SNX13 | 215820_x_at | 0.080 | 1.40 |
| PELI1 | 232213_at | 0.080 | 1.66 |
| **PLSCR4** | 218901_at | 0.080 | 2.31 |
| **MEG3** | 235077_at | 0.082 | 3.10 |
| **PRLR** | 216638_s_at | 0.082 | 1.42 |
| YPEL2 | 227020_at | 0.082 | 2.27 |
| CYSLTR2 | 220813_at | 0.084 | 1.48 |
| **GGPS1** | 202321_at | 0.084 | 0.66 |
| HGF | 209961_s_at | 0.084 | 2.15 |
| MAEL | 229475_at | 0.084 | 3.04 |
| hCG_2003663 | 233599_at | 0.085 | 1.89 |
| C12orf35 | 218614_at | 0.085 | 1.49 |
| GADD45B | 209304_x_at | 0.085 | 3.20 |
| **MTUS1** | 212096_s_at | 0.085 | 1.34 |
| **IQGAP2** | 241723_at | 0.085 | 1.52 |
| **ITGBL1** | 205422_s_at | 0.085 | 3.47 |
| RALGPS2 | 220338_at | 0.085 | 1.57 |
| CCL13 | 206407_s_at | 0.086 | 2.21 |
| HOOK3 | 236192_at | 0.086 | 1.37 |
| DKFZP564O0823 | 225809_at | 0.086 | 0.30 |
| GLTP | 226177_at | 0.088 | 0.68 |
| ITPR2 | 202661_at | 0.089 | 1.60 |
| **TMEM41A** | 235037_at | 0.089 | 0.78 |
| C17orf90 | 50374_at | 0.091 | 0.65 |
| HEY1 | 218839_at | 0.091 | 1.83 |
| HNRPLL | 225385_s_at | 0.091 | 1.56 |
| **MAF1** | 222998_at | 0.091 | 0.80 |
| PLAGL2 | 202925_s_at | 0.091 | 0.76 |
| UBR2 | 215558_at | 0.091 | 2.45 |
| AFF4 | 232864_s_at | 0.092 | 1.72 |
| EXOC6 | 232599_at | 0.092 | 2.56 |
| KIAA1712 | 228334_x_at | 0.092 | 1.34 |
| **PREX1** | 224909_s_at | 0.092 | 1.38 |
| PPIL4 | 226472_at | 0.093 | 1.49 |
| MAP3K1 | 225927_at | 0.093 | 1.40 |
| **RNF111** | 218761_at | 0.093 | 1.38 |
| **MAGEA10** | 210295_at | 0.094 | 3.31 |
| **MEG3** | 210794_s_at | 0.094 | 3.10 |
| SMAD1 | 208015_at | 0.094 | 2.42 |
| EFNB2 | 202668_at | 0.094 | 2.08 |
| JUN | 213281_at | 0.094 | 2.00 |
| ARHGAP5 | 235635_at | 0.094 | 1.53 |
| LHFP | 231411_at | 0.095 | 2.03 |
| **PDLIM5** | 203243_s_at | 0.095 | 2.10 |
| **C7** | 202992_at | 0.095 | 2.95 |
| DUSP1 | 201041_s_at | 0.095 | 3.66 |
| GLS | 221510_s_at | 0.095 | 1.75 |
| JUNB | 201473_at | 0.095 | 2.12 |
| LOC285986 | 235619_at | 0.095 | 1.74 |
| C12orf4 | 222613_at | 0.096 | 1.49 |
| ERG | 241926_s_at | 0.096 | 1.40 |
| HBEGF | 38037_at | 0.096 | 1.49 |
| **NALCN** | 228608_at | 0.096 | 1.83 |
| MEF2C | 209200_at | 0.096 | 2.11 |
| ANGPT1 | 205609_at | 0.096 | 1.92 |
| MEF2C | 209199_s_at | 0.096 | 2.20 |
| PHC3 | 215521_at | 0.096 | 1.49 |
| TM2D1 | 213882_at | 0.096 | 1.61 |
| CMAH | 229604_at | 0.096 | 1.72 |
| MCTP2 | 220603_s_at | 0.096 | 1.52 |
| **MTUS1** | 212093_s_at | 0.096 | 1.46 |
| PPP1R12B | 201957_at | 0.096 | 1.51 |
| SLC9A9 | 227791_at | 0.096 | 2.58 |
| CD47 | 227259_at | 0.096 | 2.10 |
| **PDCD5** | 227751_at | 0.096 | 1.39 |
| CD34 | 209543_s_at | 0.097 | 1.78 |
| AKT3 | 242876_at | 0.097 | 1.42 |
| CADM3 | 213948_x_at | 0.097 | 1.85 |
| NFIB | 211467_s_at | 0.097 | 1.39 |
| **SORBS2** | 233720_at | 0.097 | 1.92 |
| ZNF608 | 229817_at | 0.097 | 1.38 |
| **TTYH2** | 223741_s_at | 0.097 | 2.30 |
| F3 | 204363_at | 0.098 | 1.86 |
| GUCY1B3 | 203817_at | 0.098 | 2.30 |
| GNA13 | 224761_at | 0.100 | 1.44 |
