# Supplementary material for: Mid-Gestational Gene Expression Profile in Placenta and Link to Pregnancy Complications
Source: PLoS One. 2012 Nov 7;7(11):e49248. doi: 10.1371/journal.pone.0049248 (PMC3492272; doi:10.1371/journal.pone.0049248)
Supplement: Table S7 — TaqMan RTqPCR analysis of genes selected from placenta transcriptome micorarray anaysis. (DOCX) [file pone.0049248.s016.docx]

**Table S7. TaqMan RTqPCR analysis of genes selected from placenta transcriptome micorarray anaysis.**

Confirmation of micorarray results by RTqPCR was assessed in identical early and mid-gestation placental discovery samples followed by replication of the differential expression results in an independent non-overlapping placental sample set from early and mid-gestation. Finally, gene expression levels in mid-gestation were compared to gene expression at term placental samples from uncomplicated pregnancy.

Early-, mid- and term pregnancy placental tissues represented 5-13 weeks, 17-21 weeks and 37-41 weeks of gestational age, respectively.

Fold change was calculated as the difference of mean relative expression values. RT-qPCR data was analyzed using Student *t*-test and

*P*-values were corrected for multiple testing by estimated FDR.

| Early- *vs* mid-pregnancy | | | | | | | Mid- *vs* term-pregnancy | |
| --- | --- | --- | --- | --- | --- | --- | --- | --- |
|  | Discovery samples  n=6/4 | | Independent follow-up samples, n=17/4 | | Joint samples  n=23/8 | | n=8/12 | |
| Gene | FDR-corrected *P*-value | Fold change | FDR-corrected *P*-value | Fold change | FDR-corrected  *P*-value | Fold change | FDR-corrected  *P*-value | Fold change |
| *BACH1* | 0.19 | 1.46 | 0.27 | 1.23 | 0.096 | 1.34 | 0.76 | 1.2 |
| *BCKDK* | 0.025 | 0.82 | 0.9 | 0.92 | 0.46 | 0.89 | 0.76 | 0.99 |
| *BMP5* | 0.035 | 3.8 | 0.0017 | 4.29 | 0.00002 | 4.2 | 0.026 | 0.67 |
| *c2orf18* | 0.09 | 0.59 | 0.52 | 0.76 | 0.096 | 0.68 | 0.3 | 1.82 |
| *CCDC115* | 0.19 | 0.83 | 0.17 | 0.79 | 0.037 | 0.77 | 0.00052 | 1.6 |
| *CCNG2* | 0.27 | 1.35 | 0.0087 | 1.44 | 0.0033 | 1.41 | 0.0049 | 0.68 |
| *CDH11* | 0.09 | 1.53 | 0.028 | 1.73 | 0.0029 | 1.74 | 0.00022 | 0.31 |
| *FST* | 0.025 | 2.41 | 0.0017 | 2.89 | 0.000018 | 2.65 | 0.0013 | 0.55 |
| *GATM** | 0.025 | 2.84 | 0.0023 | 2.79 | 0.00012 | 3.1 | 0.048 | 0.66 |
| *GGPS1* | 0.035 | 0.78 | 0.9 | 0.96 | 0.23 | 0.84 | 0.09 | 1.7 |
| *GPR183*/*EBI2* | 0.025 | 3.07 | 0.0036 | 2.68 | 0.000087 | 2.83 | 0.00052 | 0.41 |
| *RSF1* | 0.27 | 1.17 | 0.82 | 0.89 | 0.92 | 0.95 | 0.0027 | 1.88 |
| *ITGBL1* | 0.025 | 2.32 | 0.0017 | 3.24 | 0.000018 | 3.04 | 0.0034 | 0.5 |
| *LYPD6* | 0.025 | 3.12 | 0.0025 | 2.96 | 0.000033 | 3 | 0.49 | 0.9 |
| *MEG3** | 0.025 | 4.57 | 0.0017 | 7.21 | 0.000018 | 6.36 | 0.7 | 1 |
| *NEDD9* | 0.025 | 1.83 | 0.017 | 1.64 | 0.00052 | 1.69 | 0.00027 | 1.4 |
| *NR3C1* | 0.09 | 1.57 | 0.033 | 1.61 | 0.0049 | 1.56 | 0.0013 | 1.68 |
| *NRCAM* | 0.09 | 3.27 | 0.012 | 2.99 | 0.0013 | 3.05 | 0.41 | 2.22 |
| *PLAGL1** | 0.025 | 2.21 | 0.0017 | 3.29 | 0.000018 | 2.97 | 0.00052 | 0.43 |
| *PUM1* | 0.61 | 1.09 | 0.53 | 1.08 | 0.44 | 1.1 | 0.79 | 1.6 |
| *SLC16A10* | 0.035 | 3.05 | 0.0056 | 2.91 | 0.00022 | 3.12 | 0.026 | 0.71 |
| *SNX18* | 0.14 | 1.3 | 0.53 | 1.1 | 0.16 | 1.17 | 0.0018 | 1.47 |
| *STC1* | 0.09 | 2.03 | 0.0017 | 2.27 | 0.000087 | 2.21 | 0.00027 | 0.24 |
| *ZFP36L1* | 0.035 | 2.5 | 0.019 | 1.74 | 0.00067 | 2.29 | 0.88 | 1.14 |

* *MEG3/meg3* imprinting has been described in human, mouse and sheep; *PLAGL1/plagl1 (ZAC/zac)* imprinting in human and mouse; and *gatm* is imprinted in mouse.
