# Supplementary material for: Mid-Gestational Gene Expression Profile in Placenta and Link to Pregnancy Complications
Source: PLoS One. 2012 Nov 7;7(11):e49248. doi: 10.1371/journal.pone.0049248 (PMC3492272; doi:10.1371/journal.pone.0049248)
Supplement: Table S8 — The identified mid-gestation marker genes showing no statistically significant difference in gene expression between the term placental samples from normal pregnancies and the pregnancy complications in the REPROMETA sample collection. (DOCX) [file pone.0049248.s017.docx]

**Table S8**. The identified mid-gestation marker genes showing no statistically significant difference in gene expression between the term placental samples from normal pregnancies and the pregnancy complications in the REPROMETA sample collection.

|  |  | Differential placental mRNA expression^b^ | |  |
| --- | --- | --- | --- | --- |
|  |  | Student t-test  (no adjustment) | |  |
| Gene | Pregnancy complication^a^ | *P*-value | FDR-corrected  *P*-value | Fold change^c^ |
| *BMP5* | PE | 0.11 | 0.28 | 0.76 |
|  | GDM | 0.39 | 0.6 | 1.23 |
|  | SGA | 0.75 | 0.86 | 0.94 |
|  | LGA | 0.063 | 0.21 | 0.74 |
| *FST* | PE | 0.51 | 0.67 | 0.89 |
|  | GDM | 0.17 | 0.38 | 1.26 |
|  | SGA | 0.94 | 0.96 | 0.99 |
|  | LGA | 0.73 | 0.85 | 1.05 |
| *ITGBL1* | PE | 0.5 | 0.67 | 1.14 |
|  | GDM | 0.46 | 0.63 | 0.88 |
|  | SGA | 0.054 | 0.2 | 0.69 |
|  | LGA | 0.052 | 0.2 | 0.68 |
| *NEDD9* | PE | 0.34 | 0.58 | 1.07 |
|  | GDM | 0.23 | 0.48 | 0.94 |
|  | SGA | 0.11 | 0.28 | 1.1 |
|  | LGA | 0.72 | 0.85 | 0.98 |
| *NR3C1* | PE | 0.94 | 0.96 | 0.99 |
|  | GDM | 0.3 | 0.56 | 1.12 |
|  | SGA | 1 | 1 | 1 |
|  | LGA | 0.86 | 0.92 | 1.02 |
| *NRCAM* | PE | 0.73 | 0.85 | 0.87 |
|  | GDM | 0.92 | 0.96 | 0.97 |
|  | SGA | 0.1 | 0.26 | 0.47 |
|  | LGA | 0.71 | 1 | 0.86 |
| *PLAGL1* | PE | 0.34 | 0.58 | 1.21 |
|  | GDM | 0.17 | 0.38 | 1.22 |
|  | SGA | 0.29 | 0.56 | 1.16 |
|  | LGA | 0.45 | 0.62 | 1.1 |
| *SLC16A10* | PE | 0.16 | 0.37 | 0.8 |
|  | GDM | 0.34 | 0.58 | 1.24 |
|  | SGA | 0.87 | 0.92 | 1.02 |
|  | LGA | 0.37 | 0.58 | 0.89 |
| *ZFP36L1* | PE | 0.73 | 0.85 | 1.09 |
|  | GDM | 0.93 | 0.96 | 0.98 |
|  | SGA | 0.25 | 0.5 | 0.72 |
|  | LGA | 0.59 | 0.74 | 0.88 |

^a^Cases included maternal (preeclampsia, PE, *n*=12; gestational diabetes mellitus, GDM, *n*=12) and fetal (small-for-gestational-age, SGA, *n*=12; large-for-gestational-age, LGA, *n*=12) pregnancy complications and the reference sample for gene expression comprised of normal uncomplicated pregnancies (*n*=12).

^b^*P*-values from RT-qPCR data were estimated using Student *t*-test (no adjustment). FDR correction considered 16 genes.

^c^Fold change was calculated as the difference of mean relative RT-qPCR expression values of each pregnancy complication vs control group of normal uncomplicated pregnancies.
