# Supplementary material for: Mid-Gestational Gene Expression Profile in Placenta and Link to Pregnancy Complications
Source: PLoS One. 2012 Nov 7;7(11):e49248. doi: 10.1371/journal.pone.0049248 (PMC3492272; doi:10.1371/journal.pone.0049248)
Supplement: Table S9 — Involvement of 16 detected mid-gestation marker genes in adult complex traits and diseases. (DOCX) [file pone.0049248.s018.docx]

**Table S9.** Involvement of 16 detected mid-gestation marker genes in adult complex traits and diseases.

| Gene ID | Gene function/Disease association | Reference |
| --- | --- | --- |
| *BMP5* | Bone and cartilage development | Sakou, T. (1998) Bone morphogenetic proteins: from basic studies to clinical approaches. *Bone*, **22**, 591-603.  Zoricic, S. *et al.* (2003) Expression of bone morphogenetic proteins and cartilage-derived morphogenetic proteins during osteophyte formation in humans. *J Anat*, **202**, 269-277.  Guenther, C. *et al.* (2008) Shaping skeletal growth by modular regulatory elements in the Bmp5 gene. *PLoS Genet*, **4**, e1000308. |
|  | Limb development | Zuzarte-Luis, V. *et al.* (2004) A new role for BMP5 during limb development acting through the synergic activation of Smad and MAPK pathways. *Dev Biol*, **272**, 39-52. |
|  | Axial skeletal abnormalities | Feldman, G.J. *et al.* (2007) Over-expression of BMP4 and BMP5 in a child with axial skeletal malformations and heterotopic ossification: a new syndrome. *Am J Med Genet A*, **143**, 699-706. |
|  | Osteoarthritis, rheumatoid arthriris | Bramlage, C.P. *et al.* (2006) Decrease in expression of bone morphogenetic proteins 4 and 5 in synovial tissue of patients with osteoarthritis and rheumatoid arthritis. *Arthritis Res Ther*, **8**, R58. |
|  | Hypertensive nephrosclerosis | Bramlage, C.P. *et al.* (2011) The role of bone morphogenetic protein-5 (BMP-5) in human nephrosclerosis. *J Nephrol*, **24**, 647-655. |
|  | Pancreatic cancer | Virtanen, S. *et al.* ()2011 Bone morphogenetic protein -4 and -5 in pancreatic cancer--novel bidirectional players. *Exp Cell Res*, **317**, 2136-2146. |
|  | Prostate and breast cancer | Bobinac, D., *et al.* (2005) Expression of bone morphogenetic proteins in human metastatic prostate and breast cancer. *Croat Med J*, **46**, 389-396. |
| *CCNG2* | Negative regulation of cell cycle progression | Bennin, D.A. *et al.* (2002) Cyclin G2 associates with protein phosphatase 2A catalytic and regulatory B' subunits in active complexes and induces nuclear aberrations and a G1/S phase cell cycle arrest. *J Biol Chem*, **277**, 27449-27467. |
|  | Adipogenesis | Aguilar, V. *et al*. (2010) Cyclin G2 regulates adipogenesis through PPAR gamma coactivation. *Endocrinology*, **151**, 5247-5254. |
|  | Implantation, decidualization | Yue, L. *et al.* (2005) Cyclin G1 and cyclin G2 are expressed in the periimplantation mouse uterus in a cell-specific and progesterone-dependent manner: evidence for aberrant regulation with Hoxa-10 deficiency. *Endocrinology*, **146**, 2424-2433. |
|  | Thyroid carcinoma | Ito, Y. *et al.* (2003) Decreased expression of cyclin G2 is significantly linked to the malignant transformation of papillary carcinoma of the thyroid. *Anticancer Res*, **23**, 2335-2338. |
|  | Gastric cancer | Choi, M.G. *et al.* (2009) Expression levels of cyclin G2, but not cyclin E, correlate with gastric cancer progression. *J Surg Res*, **157**, 168-174. |
|  | Oral cancer | Kim, Y. *et al.* (2004) Cyclin G2 dysregulation in human oral cancer. *Cancer Res*, **64**, 8980-8986. |
|  | Breast and ovarian cancer | Fu, G. and Peng, C. (2011) Nodal enhances the activity of FoxO3a and its synergistic interaction with Smads to regulate cyclin G2 transcription in ovarian cancer cells. *Oncogene*, **30**, 3953-3966. |
| *CDH11* | Cell adhesion, bone formation and morphology | Cheng, S.L. *et al.* (1998) Human osteoblasts express a repertoire of cadherins, which are critical for BMP-2-induced osteogenic differentiation. *J Bone Miner Res*, **13**, 633-644.  Farber, C.R. *et al*. (2011) Identification of quantitative trait loci influencing skeletal architecture in mice: emergence of Cdh11 as a primary candidate gene regulating femoral morphology. *J Bone Miner Res*, **26**, 2174-2183. |
|  | Tumor supressor | Li, L. *et al.* (2011) The human cadherin 11 is a pro-apoptotic tumor suppressor modulating cell stemness through Wnt/beta-catenin signaling and silenced in common carcinomas. *Oncogene*. 2011 Dec 5. doi: 10.1038/onc.2011.541. [Epub ahead of print] |
|  | Pulmonary fibrosis | Schneider, D.J. *et al.* (2012) Cadherin-11 contributes to pulmonary fibrosis: potential role in TGF-beta production and epithelial to mesenchymal transition. *FASEB J*, **26**, 503-512. |
|  | Osteoarthritis | Karlsson, C. *et al.* (2010) Genome-wide expression profiling reveals new candidate genes associated with osteoarthritis. *Osteoarthritis Cartilage*, **18**, 581-592. |
|  | Osteosarcoma | Nakajima, G. *et al.* (2008) CDH11 expression is associated with survival in patients with osteosarcoma. *Cancer Genomics Proteomics*, **5**, 37-42. |
|  | Glioblastoma | Delic, S. *et al.* (2012) Identification and functional validation of CDH11, PCSK6 and SH3GL3 as novel glioma invasion-associated candidate genes. *Neuropathol Appl Neurobiol*, **38**, 201-212. |
|  | Retinoblastoma | Marchong, M.N. *et al.* (2004) Minimal 16q genomic loss implicates cadherin-11 in retinoblastoma. *Mol Cancer Res*, **2**, 495-503. |
|  | Cancer metastasis | Chu, K. *et al.* (2008) Cadherin-11 promotes the metastasis of prostate cancer cells to bone. *Mol Cancer Res*, **6**, 1259-1267.  Li, Z. *et al.* (2008) Alterations in Cx43 and OB-cadherin affect breast cancer cell metastatic potential. *Clin Exp Metastasis*, **25**, 265-272. |
| *FST* | Folliculogenesis | Ueno, N. *et al*. (1987) Isolation and partial characterization of follistatin: a single-chain Mr 35,000 monomeric protein that inhibits the release of follicle-stimulating hormone. *Proc Natl Acad Sci U S A*, **84**, 8282-8286. |
|  | Muscle growth | Lee, S.J. *et al.* (2010) Regulation of muscle mass by follistatin and activins. *Mol Endocrinol*, **24**, 1998-2008. |
|  | Bone mineralization | Gajos-Michniewicz, A. *et al*. (2010) Follistatin as a potent regulator of bone metabolism. *Biomarkers*, **15**, 563-574. |
|  | Polycystic ovary syndrome | Eldar-Geva, T. *et al.* (2001) Follistatin and activin A serum concentrations in obese and non-obese patients with polycystic ovary syndrome. *Hum Reprod*, **16**, 2552-2556. |
|  | Recurrent miscarriage | Prakash, A. *et al.* (2006) A study of luteal phase expression of inhibin, activin, and follistatin subunits in the endometrium of women with recurrent miscarriage. *Fertil Steril*, **86**, 1723-1730. |
|  | Metastasis of prostate cancer | Tumminello, F.M. *et al.* (2010) Serum follistatin in patients with prostate cancer metastatic to the bone. *Clin Exp Metastasis*, **27**, 549-555. |
|  | Osteoarthritis | Tardif, G. *et al.* (2004) Differential gene expression and regulation of the bone morphogenetic protein antagonists follistatin and gremlin in normal and osteoarthritic human chondrocytes and synovial fibroblasts. *Arthritis Rheum*, **50**, 2521-2530. |
| *GATM /*  *AGAT* | Creatine biosynthesis, nervous system development | Wyss, M. and Kaddurah-Daouk, R. (2000) Creatine and creatinine metabolism. *Physiol Rev*, **80**, 1107-1213.  Braissant O., *et al.(*2005) Creatine synthesis and transport during rat embryogenesis: spatiotemporal expression of AGAT, GAMT and CT1. *BMC Dev Biol*, **5**:9.  Wang L*., et al.* (2007) Spatiotemporal expression of the creatine metabolism related genes agat, gamt and ct1 during zebrafish embryogenesis. *Int J Dev Biol,* **51**(3):247-53. |
|  | Heart failure | Cullen, M.E. *et al.* (2006) Myocardial expression of the arginine:glycine amidinotransferase gene is elevated in heart failure and normalized after recovery: potential implications for local creatine synthesis. *Circulation*, **114**, I16-20. |
|  | Chronic kidney disease | Kottgen, A. *et al.* (2009) Multiple loci associated with indices of renal function and chronic kidney disease. *Nat Genet*, **41**, 712-717. |
|  | Mental retardation | Battini, R. *et al.* (2006) Arginine:glycine amidinotransferase (AGAT) deficiency in a newborn: early treatment can prevent phenotypic expression of the disease. *J Pediatr*, **148**, 828-830. |
| *GPR183*/*EBI2* | Humoral immunity | Gatto, D. *et al.* (2009) Guidance of B cells by the orphan G protein-coupled receptor EBI2 shapes humoral immune responses. *Immunity*, **31**, 259-269. |
|  | Type 1 diabetes | Heinig, M. *et al.* (2010) A trans-acting locus regulates an anti-viral expression network and type 1 diabetes risk. *Nature*, **467**, 460-464. |
| *ITGBL1* | Cell adhesion | Berg, R.W. *et al*. (1999) Cloning and characterization of a novel beta integrin-related cDNA coding for the protein TIED ("ten beta integrin EGF-like repeat domains") that maps to chromosome band 13q33: A divergent stand-alone integrin stalk structure. *Genomics*, **56**, 169-178. |
|  | Growth hormone deficiency | Cody, J.D. *et al*. (2010) Identification of two novel chromosome regions associated with isolated growth hormone deficiency. *J Pediatr Endocrinol Metab*, **23**, 1159-1164. |
| *LYPD6* | Suppression of AP-1 mediated transcriptional activity | Zhang, Y. *et al.* (2010) Identification and characterization of human LYPD6, a new member of the Ly-6 superfamily. *Mol Biol Rep*, **37**, 2055-2062. |
|  | Located in genomic region implicated to developmental delay and autistic features | Chung, B.H. *et al.* (2012) Severe intellectual disability and autistic features associated with microduplication 2q23.1. *Eur J Hum Genet*, **20**, 398-403. |
| *MEG3 /*  *Gtl2*  *(Imprinted, maternally expressed)* | Negative regulation of cell proliferation | Zhou, Y. *et al*. (2007) Activation of p53 by MEG3 non-coding RNA. *J Biol Chem*, **282**, 24731-24742.  Zhou, Y. *et al.* (2012) MEG3 non-coding RNA: a tumor suppressor. *J Mol Endocrinol*. |
|  | Embryonic development | Azzi S*, et al.* (2009) Multilocus methylation analysis in a large cohort of 11p15-related foetal growth disorders (Russell Silver and Beckwith Wiedemann syndromes) reveals simultaneous loss of methylation at paternal and maternal imprinted loci. *Hum Mol Genet* **18**(24), 4724-33.  Zhou, Y., *et al.* Activation of paternally expressed genes and perinatal death caused by deletion of the Gtl2 gene. *Development*, **137**, 2643-2652. |
|  | Type 1 diabetes | Wallace, C. *et al*. (2010) The imprinted DLK1-MEG3 gene region on chromosome 14q32.2 alters susceptibility to type 1 diabetes. *Nat Genet*, **42**, 68-71. |
|  | Pituitary tumor | Zhao, J. *et al.* (2005) Hypermethylation of the promoter region is associated with the loss of MEG3 gene expression in human pituitary tumors. *J Clin Endocrinol Metab*, **90**, 2179-2186. |
|  | Meningioma | Zhang, X. *et al.* (2010) Maternally expressed gene 3, an imprinted noncoding RNA gene, is associated with meningioma pathogenesis and progression. *Cancer Res*, **70**, 2350-2358. |
|  | Acute myeloid leukemia | Benetatos, L. *et al.* (2010) CpG methylation analysis of the MEG3 and SNRPN imprinted genes in acute myeloid leukemia and myelodysplastic syndromes. *Leuk Res*, **34**, 148-153. |
| *NEDD9* | Cell adhesion, proliferation | Singh, M. *et al.* (2007) Molecular basis for HEF1/NEDD9/Cas-L action as a multifunctional co-ordinator of invasion, apoptosis and cell cycle. *Cell Biochem Biophys*, **48**, 54-72. |
|  | Alzheimer’s and Parkinson’s disease | Chapuis J., *et al.* (2008) Association study of the NEDD9 gene with the risk of developing Alzheimer's and Parkinson's disease. *Hum Mol Genet* **17**(18):2863-7.  Li Y., *et al.* (2008) Evidence that common variation in NEDD9 is associated with susceptibility to late-onset Alzheimer's and Parkinson's disease. *Hum Mol Genet* **17**(5):759-67.  Tedde, A. *et al.* (2010) Different implication of NEDD9 genetic variant in early and late-onset Alzheimer's disease. *Neurosci Lett*, **477**, 121-123. |
|  | Melanoma | Kim, M. *et al.* (2006) Comparative oncogenomics identifies NEDD9 as a melanoma metastasis gene. *Cell*, **125**, 1269-1281. |
|  | Head and neck squamous cell carcinoma | Lucas, J.T. *et al*. (2010) Regulation of invasive behavior by vascular endothelial growth factor is HEF1-dependent. *Oncogene*, **29**, 4449-4459. |
|  | Glioblastoma | Natarajan, M. iet al. (2006) HEF1 is a necessary and specific downstream effector of FAK that promotes the migration of glioblastoma cells. *Oncogene*, **25**, 1721-1732. |
|  | Colon cancer | Xia, D. *et al*. (2010) HEF1 is a crucial mediator of the proliferative effects of prostaglandin E(2) on colon cancer cells. *Cancer Res*, **70**, 824-831. |
| *NR3C1* | Transcriptional regulation, chromatin remodeling, inflammatory response, growth control | Kumar, R. and Thompson, E.B. (2005) Gene regulation by the glucocorticoid receptor: structure:function relationship. *J Steroid Biochem Mol Biol*, **94**, 383-394.  Barnes, P.J. (1998) Anti-inflammatory actions of glucocorticoids: molecular mechanisms. *Clin Sci (Lond)*, **94**, 557-572. |
|  | Glucocorticoid resistance | Charmandari, E. *et al.* (2008) Generalized glucocorticoid resistance: clinical aspects, molecular mechanisms, and implications of a rare genetic disorder. *J Clin Endocrinol Metab*, **93**, 1563-1572. |
|  | Coronary heart disease | van den Akker, E.L. *et al.* (2008) Glucocorticoid receptor gene and risk of cardiovascular disease. *Arch Intern Med*, **168**, 33-39. |
|  | Pathogenesis of rheumatic diseases | Kino, T. *et al.* (2011) Glucocorticoid receptor: implications for rheumatic diseases. *Clin Exp Rheumatol*, **29**, S32-41. |
|  | Colorectal cancer | Lind, G.E. *et al.* (2006) ADAMTS1, CRABP1, and NR3C1 identified as epigenetically deregulated genes in colorectal tumorigenesis. *Cell Oncol*, **28**, 259-272. |
|  | Acute lymphoblastic leukemia | Labuda, M. *et al*. (2010) Polymorphisms in glucocorticoid receptor gene and the outcome of childhood acute lymphoblastic leukemia (ALL). *Leuk Res*, **34**, 492-497. |
|  | Obesity | Lin, R.C. *et al*. (2003) Association of obesity, but not diabetes or hypertension, with glucocorticoid receptor N363S variant. *Obes Res*, **11**, 802-808. |
|  | Adrenocortical carcinoma | Tacon, L.J. *et al.* (2009) The glucocorticoid receptor is overexpressed in malignant adrenocortical tumors. *J Clin Endocrinol Metab*, **94**, 4591-4599. |
| *NRCAM* | Nervous system development, cell adhesion | Grumet, M. (1991) Cell adhesion molecules and their subgroups in the nervous system. *Curr Opin Neurobiol*, **1**, 370-376. |
|  | Autism | Marui, T. *et al.* (2009) Association of the neuronal cell adhesion molecule (NRCAM) gene variants with autism. *Int J Neuropsychopharmacol*, **12**, 1-10. |
|  | Addiction | Ishiguro, H. *et al*. (2006) NrCAM in addiction vulnerability: positional cloning, drug-regulation, haplotype-specific expression, and altered drug reward in knockout mice. *Neuropsychopharmacology* **31**, 572-584. |
|  | Melanoma and colon cancer | Conacci-Sorrell, M.E. *et al*. (2002) Nr-CAM is a target gene of the beta-catenin/LEF-1 pathway in melanoma and colon cancer and its expression enhances motility and confers tumorigenesis. *Genes Dev*, **16**, 2058-2072. |
|  | Pancreatic cancer | Dhodapkar, K.M. *et al*. (2001) Differential expression of the cell-adhesion molecule Nr-CAM in hyperplastic and neoplastic human pancreatic tissue. *Hum Pathol*, **32**, 396-400. |
|  | Papillary thyroid cancer | Gorka, B. *et al*. (2007) NrCAM, a neuronal system cell-adhesion molecule, is induced in papillary thyroid carcinomas. *Br J Cancer*, **97**, 531-538. |
| *PLAGL1/*  *ZAC*  *(Imprinted, paternally expressed)* | Embryonic growth | Varrault, A., *et al.* (2006) Zac1 regulates an imprinted gene network critically involved in the control of embryonic growth. *Dev Cell*, **11**, 711-722.  Valente, T., *et al*. (2005) Zac1 is expressed in progenitor/stem cells of the neuroectoderm and mesoderm during embryogenesis: differential phenotype of the Zac1-expressing cells during development. *Dev Dyn*, **233**, 667-679. |
|  | Ovarian, breast and gastric cancer, melanoma, astrocytoma, pancreatic adenocarcinoma, renal cell carcinomas | Varrault, A. *et al*. (1998) hZAC encodes a zinc finger protein with antiproliferative properties and maps to a chromosomal region frequently lost in cancer. *Proc Natl Acad Sci U S A*, **95**, 8835-8840. |
|  | Heart development | Yuasa, S. *et al.* (2010) Zac1 is an essential transcription factor for cardiac morphogenesis. *Circ Res*, **106**, 1083-1091. |
|  | Pancreas development | Du, X. *et al.* (2011) Differential expression pattern of ZAC in developing mouse and human pancreas. *J Mol Histol*, **42**, 129-136. |
|  | Pheochromocytoma | Jarmalaite, S. *et al.* (2011) Tumor suppressor gene ZAC/PLAGL1: altered expression and loss of the nonimprinted allele in pheochromocytomas. *Cancer Genet*, **204**, 398-404. |
|  | Capillary hemangioblastoma | Lemeta, S. *et al*. (2007) Preferential loss of the nonimprinted allele for the ZAC1 tumor suppressor gene in human capillary hemangioblastoma. *J Neuropathol Exp Neurol*, **66**, 860-867. |
|  | Pituitary adenoma | Pagotto, U. *et al*. (2000) The expression of the antiproliferative gene ZAC is lost or highly reduced in nonfunctioning pituitary adenomas. *Cancer Res*, **60**, 6794-6799. |
|  | B-cell non-Hodgkin's lymphomas | Zhang, Y. *et al*. (1997) Frequent deletions of 6q23-24 in B-cell non-Hodgkin's lymphomas detected by fluorescence in situ hybridization. *Genes Chromosomes Cancer*, **18**, 310-313. |
| *SLC16A10* | Aromatic amino acids transport | Kim, D.K. *et al.* (2002) The human T-type amino acid transporter-1: characterization, gene organization, and chromosomal location. *Genomics*, **79**, 95-103. |
|  | Thyroid hormone transport | Friesema, E.C. *et al.* (2008) Effective cellular uptake and efflux of thyroid hormone by human monocarboxylate transporter 10. *Mol Endocrinol*, **22**, 1357-1369. |
|  | Intrauterine growth restriction | Loubiere, L.S. *et al*. (2010) Expression of thyroid hormone transporters in the human placenta and changes associated with intrauterine growth restriction. *Placenta*, **31**, 295-304. |
| *STC1* | Renal and intestinal Ca2+/P homeostasis, angiogenesis, bone and muscle development | Yoshiko, Y. Aubin, J.E. (2004) Stanniocalcin 1 as a pleiotropic factor in mammals. *Peptides*, **25**, 1663-1669.  Yeung, B.H.Y., *et al.* (2012) Evolution and roles of stanniocalcin. *Mol Cell Endocrinol* **349,** 272–280. |
|  | Growth regulation, reproduction | Varghese, R. *et al.* (2002) Overexpression of human stanniocalcin affects growth and reproduction in transgenic mice. *Endocrinology*, **143**, 868-876.  Johnston, J., *et al.* (2010) Human stanniocalcin-1 or -2 expressed in mice reduces bone size and severely inhibits cranial intramembranous bone growth. *Transgenic Res*, **19**, 1017-1039. |
|  | Regulation of gestation and lactation | Deol, H.K. *et al.* (2000) Dynamic regulation of mouse ovarian stanniocalcin expression during gestation and lactation. *Endocrinology*, **141**, 3412-3421. |
|  | Renal glomerula filtration rate and chronic kidney disease | Kottgen, A. *et al.* (2009) Multiple loci associated with indices of renal function and chronic kidney disease. *Nat Genet*, **41**, 712-717. Böger CA, *et al*.; CKDGen Consortium (2011) Association of eGFR-Related Loci Identified by GWAS with Incident CKD and ESRD. *PLoS Genet* 7(9):e1002292. |
|  | Heart failure | Sheikh-Hamad, D. *et al.* (2003) Stanniocalcin-1 is a naturally occurring L-channel inhibitor in cardiomyocytes: relevance to human heart failure. *Am J Physiol Heart Circ Physiol*, **285**, H442-448. |
|  | Colorectal, ovarian, hepatocellular and breast cancer | Chang, A.C. *et al*. (2003) Mammalian stanniocalcins and cancer. *Endocr Relat Cancer*, **10**, 359-373. |
| *ZFP36L1* | Response to growth factors | Hacker, C. *et al.* (2010) ZFP36L1 is regulated by growth factors and cytokines in keratinocytes and influences their VEGF production. *Growth Factors*, **28**, 178-190. |
|  | Abnormal placentation and fetal death | Stumpo, D.J. *et al.* (2004) Chorioallantoic fusion defects and embryonic lethality resulting from disruption of Zfp36L1, a gene encoding a CCCH tandem zinc finger protein of the Tristetraprolin family. *Mol Cell Biol*, **24**, 6445-6455. |
|  | Acute myelogenous leukemia, T-cell leukemia/lymphoma | Hodson, D.J. *et al.* (2010) Deletion of the RNA-binding proteins ZFP36L1 and ZFP36L2 leads to perturbed thymic development and T lymphoblastic leukemia. *Nat Immunol*, **11**, 717-724. |
|  | Breast cancer | Abba, M.C. *et al.* (2007) Breast cancer molecular signatures as determined by SAGE: correlation with lymph node status. *Mol Cancer Res*, **5**, 881-890. |
